# Supplementary material for: Systematic review of the healthcare cost of bronchopulmonary dysplasia
Source: BMJ Open. 2021 Aug 10;11(8):e045729. doi: 10.1136/bmjopen-2020-045729 (PMC8356184; doi:10.1136/bmjopen-2020-045729)
Supplement: Supplementary data [file bmjopen-2020-045729supp001.pdf]

SUPPLEMENTAL MATERIAL

SYSTEMATIC REVIEW OF THE HEALTHCARE COST OF BRONCHOPULMONARY DYSPLASIA

AUTHORS

Jhangir Humayun[1,2], gushumjh@student.gu.se

Chatarina Löfqvist[1,2,3] chatarina.lofqvist@gu.se

David Ley[4], david.ley@med.lu.se

Ann Hellström[3], ann.hellstrom@medfak.gu.se

Hanna Gyllensten[1,2] hanna.gyllensten@gu.se

Author affiliations

[1] Institute of Health and Care Sciences, Sahlgrenska Academy, University of Gothenburg, Box 457, SE-405 30 Gothenburg, Sweden.

[2] Centre for Person-Centred Care (GPCC), University of Gothenburg, Box 457, SE-405 30 Gothenburg, Sweden.

[3] Department of Clinical Neuroscience, Institute of Neuroscience and Physiology, Sahlgrenska Academy, University of Gothenburg, Gothenburg, Sweden;

[4] Department of Pediatrics, Institute of Clinical Sciences Lund, Lund University and Skåne University Hospital, Lund, Sweden

Table S1: Search terms

Database	Search string
PubMed	((("Bronchopulmonary Dysplasia"[Title/Abstract]) OR "Bronchopulmonary Dysplasia"[Mesh])) AND (("Economics"[Mesh]) OR ((economic*[Title/Abstract] OR cost[Title/Abstract] OR costs[Title/Abstract] OR costly[Title/Abstract] OR costing[Title/Abstract] OR price[Title/Abstract] OR prices[Title/Abstract] OR pricing[Title/Abstract] OR pharmacoeconomic*[Title/Abstract])))
Scopus	(TITLE-ABS-KEY ("Bronchopulmonary Dysplasia")) AND (TITLE-ABS-KEY (economic* OR cost OR costs OR costly OR costing OR price OR prices OR pricing OR pharmacoeconomic*))

Table S2: Checklist for the quality appraisal of included papers (from [15])

Checklist items	24	33	32	29	31	30	28	27	26	25	23	22	21	Total*
Is the study population clearly described?	-	+	+	-	+	+	+	+	+	+	-	+	-	9
Are competing alternatives clearly described?	NA	NA	NA	NA	NA	+	NA	NA	+	+	NA	NA	NA	3
Is a well-defined research question posed in answerable form?	-	-	-	-	-	-	-	-	-	-	-	-	-	0
Is the economic study design appropriate to the stated objective?	+	+	+	+	+	+	+	+	+	+	+	+	+	13
Is the chosen time horizon appropriate in order to include relevant costs and consequences?	NA	NA	NA	NA	NA	NA	NA	NA	NA	NA	NA	NA	NA	0
Is the actual perspective chosen appropriate?	+	+	+	+	+	+	+	+	+	+	+	+	+	13
Are all important and relevant costs for each alternative identified?	+	+	+	+	+	+	-	+	+	+	+	+	+	12
Are all costs measured appropriately in physical units?	+	+	+	+	+	+	+	-	+	+	+	+	+	12
Are costs valued appropriately?	+	+	+	+	+	+	+	+	+	+	+	+	+	13
Are all important and relevant outcomes for each alternative identified?	NA	NA	NA	NA	NA	NA	NA	NA	NA	NA	NA	NA	NA	0
Are all outcomes measured appropriately?	NA	NA	NA	A	NA	NA	NA	NA	NA	NA	NA	NA	NA	0
Are outcomes valued appropriately?	NA	NA	NA	NA	NA	NA	NA	NA	NA	NA	NA	NA	NA	0
Is an incremental analysis of costs and outcomes of alternatives performed?	NA	NA	NA	NA	NA	NA	NA	NA	NA	NA	NA	NA	NA	0
Are all future costs and outcomes discounted appropriately?	NA	NA	NA	NA	+	NA	+	+	+	+	-	+	+	7
Are all important variables, whose values are uncertain, appropriately subjected to sensitivity analysis?	NA	-	-	-	+	-	-	-	-	-	-	-	-	1
Do the conclusions follow from the data reported?	-	+	+	+	+	+	+	+	+	+	+	+	+	12
Does the study discuss the generalizability of the results to other settings and patient/client groups?	-	+	+	+	+	+	+	+	+	+	+	+	+	12
Does the article indicate that there is no potential conflict of interest of study researcher(s) and funder(s)?	-	+	+	+	+	+	+	+	+	+	-	-	-	9
Are ethical and distributional issues discussed appropriately?	+	+	+	+	+	+	+	+	+	+	+	+	+	13
Number of checklist items fulfilled by each individual study	6	10	10	9	12	11	10	10	12	12	8	10	9	

+ indicates a fulfilled checklist item, - indicates that the item was either not stated or not fulfilled, NA indicates that the checklist item was not relevant for that study

* Number of studies that fulfilled each checklist item (not considering the applicability of the checklist item to the individual studies
