# Supplementary material for: Systematic review of the healthcare cost of bronchopulmonary dysplasia
Source: BMJ Open. 2021 Aug 10;11(8):e045729. doi: 10.1136/bmjopen-2020-045729 (PMC8356184; doi:10.1136/bmjopen-2020-045729)
Supplement: Author's manuscript [file bmjopen-2020-045729.draft_revisions.pdf]

BMJ Open is committed to open peer review. As part of this commitment we make the peer review history of every article we publish publicly available.

When an article is published we post the peer reviewers' comments and the authors' responses online. We also post the versions of the paper that were used during peer review. These are the versions that the peer review comments apply to.

The versions of the paper that follow are the versions that were submitted during the peer review process. They are not the versions of record or the final published versions. They should not be cited or distributed as the published version of this manuscript.

BMJ Open is an open access journal and the full, final, typeset and author-corrected version of record of the manuscript is available on our site with no access controls, subscription charges or pay-per-view fees (http://bmjopen.bmj.com).

If you have any questions on BMJ Open's open peer review process please email info.bmjopen@bmj.com

BMJ Open

SYSTEMATIC REVIEW OF THE HEALTHCARE COST OF BRONCHOPULMONARY DYSPLASIA

Journal:	*BMJ Open*
Manuscript ID	bmjopen-2020-045729
Article Type:	Original research
Date Submitted by the Author:	12-Oct-2020
Complete List of Authors:	Humayun, Jhangir; University of Gothenburg Institute of Health and Care Sciences; GPCC<br>Löfqvist, Chatarina; University of Gothenburg Institute of Health and Care Sciences, Sahlgrenska Academy; University of Gothenburg Institute of Neuroscience and Physiology, Department of Clinical Neuroscience<br>Ley, David; Lund University, Department of Pediatrics, Institute of Clinical Sciences and Skåne University Hospital<br>Hellstrom, Ann; University of Gothenburg Institute of Neuroscience and Physiology, Department of Clinical Neuroscience<br>Gyllensten, Hanna; University of Gothenburg Institute of Health and Care Sciences; GPCC,
Keywords:	HEALTH ECONOMICS, PAEDIATRICS, RESPIRATORY MEDICINE (see Thoracic Medicine), Chronic airways disease < THORACIC MEDICINE, Paediatric thoracic medicine < THORACIC MEDICINE

SCHOLARONE™
Manuscripts

I, the Submitting Author has the right to grant and does grant on behalf of all authors of the Work (as defined in the below author licence), an exclusive licence and/or a non-exclusive licence for contributions from authors who are: i) UK Crown employees; ii) where BMJ has agreed a CC-BY licence shall apply, and/or iii) in accordance with the terms applicable for US Federal Government officers or employees acting as part of their official duties; on a worldwide, perpetual, irrevocable, royalty-free basis to BMJ Publishing Group Ltd ("BMJ") its licensees and where the relevant Journal is co-owned by BMJ to the co-owners of the Journal, to publish the Work in this journal and any other BMJ products and to exploit all rights, as set out in our licence.

The Submitting Author accepts and understands that any supply made under these terms is made by BMJ to the Submitting Author unless you are acting as an employee on behalf of your employer or a postgraduate student of an affiliated institution which is paying any applicable article publishing charge ("APC") for Open Access articles. Where the Submitting Author wishes to make the Work available on an Open Access basis (and intends to pay the relevant APC), the terms of reuse of such Open Access shall be governed by a Creative Commons licence – details of these licences and which Creative Commons licence will apply to this Work are set out in our licence referred to above.

Other than as permitted in any relevant BMJ Author's Self Archiving Policies, I confirm this Work has not been accepted for publication elsewhere, is not being considered for publication elsewhere and does not duplicate material already published. I confirm all authors consent to publication of this Work and authorise the granting of this licence.

For peer review only

Systematic review of the healthcare cost of Bronchopulmonary Dysplasia

Authors

Jhangir Humayun[1,2], gushumjh@student.gu.se

Chatarina Löfqvist[1,2,3] chatarina.lofqvist@gu.se

David Ley[4], david.ley@med.lu.se

Ann Hellström[3], ann.hellstrom@medfak.gu.se

Hanna Gyllensten[1,2] hanna.gyllensten@gu.se

Author affiliations

[1] Institute of Health and Care Sciences, Sahlgrenska Academy, University of Gothenburg, Box 457, SE-405 30 Gothenburg, Sweden.

[2] Centre for Person-Centred Care (GPCC), University of Gothenburg, Box 457, SE-405 30 Gothenburg, Sweden.

[3] Department of Clinical Neuroscience, Institute of Neuroscience and Physiology, Sahlgrenska Academy, University of Gothenburg, Gothenburg, Sweden;

[4] Department of Pediatrics, Institute of Clinical Sciences Lund, Lund University and Skåne University Hospital, Lund, Sweden

Contact, corresponding author

Hanna Gyllensten, Institute of Health and Care Sciences, Sahlgrenska Academy, University of Gothenburg, Postal Address: Box 457, SE-405 30 Gothenburg, Sweden, E-mail: hanna.gyllensten@gu.se. Phone: +46-(0)70-748 24 12

Word count (excluding title page, abstract, references, figures and tables): 3725

For peer review only - http://bmjopen.bmj.com/site/about/guidelines.xhtml

ABSTRACT

Objectives

To determine the costs directly or indirectly related to bronchopulmonary dysplasia (BPD) in preterm infants. The secondary objective was to stratify the costs, based on gestational age and/or birth weight.

Design

Systematic literature review

Setting

PubMed and Scopus were searched on 3 Feb 2020.

Studies were selected based on eligibility criteria by two independent reviewers.

Data were extracted by two independent reviewers with a pre-specified data extraction sheet. The costs in the included studies are reported descriptively.

Primary outcome measure

Costs of BPD.

Results

The 13 included studies reported the total costs or marginal costs of BPD. A majority of studies reported costs during birth hospitalization and/or during the first year of life. One study reported costs during the first two years; two other studies reported costs later, during the pre-school period; and one study included a long-term follow up.

Conclusions

This study was the first systematic review of costs associated with BPD. We confirmed previous reports of high costs and described the long-term follow-up necessary for preterm infants with BPD, particularly infants of very low gestational age. Moreover, we identified a need for studies that estimate costs that occur outside of hospitals and after the first year of life.

Registration

The protocol is registered in PROSPERO (registration number CRD42020173234).

For peer review only

For peer review only - http://bmjopen.bmj.com/site/about/guidelines.xhtml

ARTICLE SUMMARY

Strengths and limitations of this study

- The main strength was that the systematic search, study selection, and data extraction were conducted by independent reviewers.
- The main limitations were the lack of search strategies for identifying grey literature and the lack of searches based on the references/citations of the included studies.

For peer review only

For peer review only - http://bmjopen.bmj.com/site/about/guidelines.xhtml

INTRODUCTION

Bronchopulmonary dysplasia (BPD) is a serious lung condition that can affect new-borns. It is particularly common in premature infants, particularly infants that require oxygen therapy. Infants born more than 10 weeks preterm are more susceptible to BPD, because they often require a long treatment period with different types of breathing support, such as nasal continuous positive airway pressure, ventilators, different bronchodilators, and steroids.[1]

The definition of BPD has varied considerably over time. The condition was first described by Northway et al., in 1967, as a radiographic pattern of lung injury observed in prematurely born infants after the use of extensive mechanical ventilation.[2] Considerable strides in care have been made for these infants over the past decades, including, but not limited to, the use of antenatal steroids, surfactant therapy, vitamin A supplementation, and caffeine treatment.[3-5] The administration of antenatal steroids and exogenous surfactant has greatly reduced the severity of respiratory distress syndrome, and consequently, increased the survival of preterm infants.[6] Prior to the widespread use of antenatal steroids and surfactant, BPD in preterm infants was mainly attributed to lung injury caused by barotrauma and oxygen toxicity.[6] The pathogenesis of the BPD observed currently, in the post surfactant era, is multifactorial; various causes have been found, including extreme prematurity, infection, nutritional deprivation, and the need for prolonged mechanical ventilation.[7 8] It is imperative to understand this new form of BPD and its effects during infancy and beyond, because the survival of preterm infants is increasing.[9]

Currently, BPD is commonly defined as a condition that requires oxygen administration beyond 36 weeks postmenstrual age (PMA). Depending on the required respiratory support and the concentration of inhaled oxygen, BPD severity is classified as light, moderate, or severe. Co-morbidities and maternal infections increase the risk of developing serious BPD after a preterm birth. Disease severity varies considerably; for example, in mild cases, infants might only present with an accelerated respiratory rate; but in severe cases, mechanical ventilation may be required for a prolonged period of time.[1] Indeed, serious conditions often require continuous oxygen therapy for several months.

The incidence of BPD seems to be inversely related to the gestational age (GA, reported in weeks) at birth. Studies that included infants born at low GAs have reported higher incidences of BPD.[10] The National Institute for Child Health and Human Development neonatal network periodically reports the incidence of BPD. They define BPD as a condition that requires oxygen administration at 36 weeks PMA. They reported BPD incidences of 52% in infants with birth weights of 501 - 750 g, 34% in infants with birth weights of 751-1000 g, and 15% in infants with birth weights of 1001-1200 g. Among infants that weighed 1201-1500 g, the incidence was as low as 7%.[11]

Foetal growth restriction is commonly defined as a low birth weight, corrected for GA. Foetal growth restriction has a clear impact on the incidence and severity of BPD. Studies have shown that, among infants born at a similar GA, infants that were small for their GA (SGA) had a higher BPD incidence than infants that had normal birth weights for their GA.[10] Globally, the survival rates of extremely preterm infants (GA <28 weeks) have varied considerably, according to the economic setting. Highly developed countries have reported extremely preterm infant survival rates as high as ~90%, but in low-income settings, it can be as low as ~10%.[12] Similarly, the incidence of BPD in preterm infants varies considerably with the degree of economic development in the country of birth.

The effects of BPD on infant health extend beyond the initial hospitalization. Infants with BPD are hospitalized to a much greater extent than infants without BPD.[13] Lung function abnormalities, such

For peer review only - http://bmjopen.bmj.com/site/about/guidelines.xhtml

as small airways, often persist for a long period of time. Long-term effects, such as reduced lung function and obstructive airway disease, are more prevalent among individuals that were born prematurely than among their peers that were born full-term. Among preterm infants, those with BPD exhibit a greater need for respiratory medications, compared to those without BPD.[13] Neurodevelopment can be severely affected by BPD. Infants with BPD have an increased rate of abnormal neurodevelopment, including cognitive impairments and cerebral palsy. In severe cases of BPD, movement morbidities can occur, like chorea and akathisia.[14]

An economic evaluation of healthcare costs is important for aiding decision-making bodies to prioritize healthcare funding effectively and understand the associated long-term costs. A review [14] was previously published on the healthcare and societal cost of BPD, but the search was not systematic; all the articles were handpicked by the authors. To our knowledge, no systematic review has been published that discussed the healthcare costs of BPD. Thus, there was a need to compile and present the available data on this subject in the form of a systematic review.

The present review aimed to present all available data that pertained to the costs related to BPD, directly or indirectly, in preterm infants. The secondary objective was to stratify those costs, based on the GA or birth weight of the infant.

METHODS

This study was conducted according to the Preferred Reporting Items for Systematic Review and Meta-Analysis (PRISMA) recommendations.[5] The protocol is registered in PROSPERO (registration number CRD42020173234).

Search strategy

In collaboration with personnel of the Medicine Library at the University of Gothenburg, the authors determined that the PubMed and Scopus databases were the best choice for this review. The search was conducted on 3 Feb 2020 in both databases. Search terms (Appendix, Table S1) were drafted and discussed individually for both databases. To ensure important data were extracted consistently from relevant articles, we created an extraction table. The search was performed, and articles were included according to the criteria listed below. No restrictions were applied for publication date, country of origin, or language.

Study selection

We included observational and interventional studies that investigated the costs associated with BPD. The study population comprised preterm human infants with BPD. Two reviewers (JH and HG) independently screened the titles and abstracts of identified studies to assess their eligibility for inclusion. After reviewing the titles and abstracts, duplicates were removed. Then, the reviewers obtained full texts of potentially eligible articles and assessed them independently to determine which articles met the predetermined inclusion criteria. When the reviewers were uncertain of the eligibility of a study, a third reviewer (CL) participated in the decision.

Data extraction

Data from eligible studies were independently extracted by two reviewers (JH and HG). Any differences were resolved by discussion and consultation with a third reviewer (CL). The predesigned

For peer review only - http://bmjopen.bmj.com/site/about/guidelines.xhtml

For peer review only

data extraction table included, but was not limited to the following data: the name of first author, year of publication, study design, location, sample size, healthcare costs, and description of the costs included. When available, healthcare costs were stratified according to the GA at birth or birth weight. The two authors independently assessed the quality of the selected studies, based on predetermined quality assessment criteria for health economic studies (the CHEC-list) described previously by Evers et al.[15]

Analyses

Costs were translated to USD, based on the purchasing power parities of each year.[16] Then, the costs were inflated to 2019 values with the Organisation for Economic Co-operation and Development (OECD) inflation factor.[17] Costs were analysed, based on the age of the participants included in each study and by their GA or birth weight.

Results

The initial search results yielded 274 articles. After removing duplicates (Figure 1), 262 unique articles were screened, and 195 were excluded based on the abstract or title. Among the 66 full articles screened, 13 [18-30] unique studies met the inclusion criteria. The included studies were conducted in 1981-2015 (Table 1), in the US (7 studies), UK (2 studies), Spain, Canada, Turkey, and France. Six studies defined BPD as oxygen dependence beyond 28 days after birth, and some of these studies also classified BPD as mild/moderate/severe, based on whether oxygen was administered at 36 weeks PMA. In four studies, the definition for BPD included an oxygen requirement beyond 36 weeks PMA, which, according to Jobe and Bancalari,[31] indicated moderate/severe BPD. Two additional studies defined BPD based only on the ICD.9 (770.7) code from a registry, without reference to GA or time since birth at diagnosis. In the oldest included study,[18] the definition was based on hospital data, but they did not state whether they used ICD codes or what time-period they used for the oxygen requirement. However, their definition was consistent with the classical definition of BPD,[2] which was based on clinical signs. In two studies, the definition of BPD required a chest x-ray consistent with BPD.[19 20]

For peer review only - http://bmjopen.bmj.com/site/about/guidelines.xhtml

Table 1: Overview of studies included in this review

#	First author (year)	Country (study period)	Study design	Sample size	Time horizon	Healthcare cost with BPD	Costs included
1	Lapcharoensap (2020)[30]	US (2008-2011)	Retrospective cohort	BPD: 2,696<br>Controls: 5,302	1 year after birth, including index hospitalization	$442,468	Direct hospital costs
2	Mowitz (2019)[29]	US (2009-2015)	Retrospective cohort	BPD: 4,904<br>Controls: 7,113	Index hospitalization	$225,204	Direct hospital costs
3	Álvarez-Fuente (2017)[26]	Spain (2013)	Model	BPD: 2,628	Index hospitalization + 2 year follow up	€45,049-118,760	Hospital costs and follow-up cost
4	Patel (2017)[27]	US (2009-2012)	Prospective cohort	BPD: 77<br>Controls: 177	Index hospitalization	$269,004 (Median)	Hospital costs, overhead, and physician payments
5	Stevens (2017)[28]	US (2008-2011)	Model, Retrospective cohort	Total: 2,021,013<br>BPD: 2189	Index hospitalization + 1 year after birth	$133,140<br>Cost increase = 22.4%	Commercial claims data
6	Johnson (2013)[25]	US (2005-2009)	Prospective cohort	BPD: 230<br>Controls: 195	Index hospitalization	$103,151	Hospital costs excluding physician fees
7	Landry (2012)[24]	Canada (1983-2008)	Retrospective cohort	BPD: 773<br>Controls: 2669	16 - 25 years follow up after initial hospitalization	C$13,472	Hospitalization follow-up cost and prescription drugs
8	Greenough (2011)[23]	UK (2002-2005)	Retrospective cohort	BPD: 160	2 years at 5-7 years of age	£523-705 (Median)	Hospital admission costs, follow-up cost, and prescription drugs
9	Greenough (2006)[22]	UK (1999-2002)	Retrospective cohort	BPD: 190	2 years at 2-4 years of age	£950-3,054 (Median)	Hospital admission costs, follow-up cost, and prescription drugs
10	Akman (2002)[21]	Turkey (1999-2000)	Retrospective cohort	BPD: 72	Index hospitalization	$14,810 (Marginal cost)	Hospital charges
11	Miller (1998)[20]	US (1990)	Retrospective cohort	BPD:89	4.5 years after initial hospitalization	$19,034.3*	Direct health costs, non-health costs, and indirect cost to families
12	McAleese (1993)[19]	US (1981-1989)	Retrospective cohort	BPD: 59	Index hospitalization + home care	Hospitalization = $197,668<br>Home care = $4,262-68,136 (Medians)	Hospital charges, follow-up cost, home care equipment, and indirect cost to families

For peer review only - http://bmjopen.bmj.com/site/about/guidelines.xhtml

For peer review only

13	Monset-Couchard (1984)[18]	France (1981)	Retrospective cohort	Not stated	Index hospitalization	₣336,545	Hospital charges

Unless otherwise stated, the costs are the mean costs reported in the original paper. Abbreviations: C= Controls; C$= Canadian dollar; ₣= French franc.

*Mean cost was computed based on the data available in the article

For peer review only

For peer review only - http://bmjopen.bmj.com/site/about/guidelines.xhtml

Risk of bias in included studies

Potential sources of bias included the lack of participant characteristics in some of the included studies. Additionally, some studies lacked information about potential conflicts of interests for the participating researchers (Supplemental table S2). Only one study conducted a sensitivity analysis of their results. None of the included papers clearly stated the research question, but it could be gleaned from the objective of each study. Few studies included comparisons between groups or long-term outcomes, and no study included an evaluation of health outcomes. Thus, some of the CHEC-list items were not suitable for evaluating these studies.

Total costs for BPD

A majority of studies (Table 2) reported costs during the birth hospitalization and/or the first year of life (8 studies). One study reported follow up costs during the first two years of life including birth hospitalisation. Two other studies reported two-year follow-up costs for age groups 2-4 and 5-7 years, respectively. Two studies included a long-term follow up, with an average follow-up of 4.5 years and 19.3 years, respectively (Figure 2).

Table 2: Costs for infants with BPD

**Reference** *subgroups reported in the paper*	**Total cost (Int$)**	**Cost of index Hospitalization (Int$)**	**Cost of other care (Int$)**	**Cost increase over controls due to BPD, Int$ (%)**
**Lapcharoensap (2020)**[30]	477,901	466,216	Rehospitalization: 46,948	246,284 (106%)
≤ 24 weeks	*507,638*			*118,808 (35%)*
25- 26 weeks	*453,633*			*129,609 (40%)*
27-28 weeks	*351,026*			*124,209 (55%)*
> 28 weeks	*313,223*			*151,211 (93%)*
**Mowitz (2019)**[29]	see index hospitalization	242,995		58,381 (31%)
≤ 24 weeks	see index hospitalization	*326,962*		*26,975 (9%)*
25- 26 weeks	see index hospitalization	*247,901*		*32,370 (15%)*
27-28 weeks	see index hospitalization	*190,983*		*36,686 (24%)*
> 28 weeks	-	-		-
**Álvarez-Fuente (2017)**[26]	74,618 - 196,713		2-year follow-up:	-
<750g	*196,713*	*182,627*	*14,084*	-
750- 999g	*149,071*	*134,986*	*14,084*	-

For peer review only - http://bmjopen.bmj.com/site/about/guidelines.xhtml

1000-1249g	*73,237*	*60,534*	*14,084*	-
> 1249 g	-	-	-	-
**Patel (2017)[27]**	see index hospitalization	290,546*<br>Marginal cost: 45,287*		164,092*
**Stevens (2017)[28]**	148,251	-	-	(22.4%)
**Johnson (2013)[25]**	see index hospitalization	122,983		69,969 (132%)
<750g				*51,639*
750- 999g				*41,196*
1000-1249g				*29,317*
> 1249 g				*23,810*
**Landry (2012)[24]**	12,973/year		Follow-up: 12,973/year	2,651/year (26%)
**Greenough (2011)[23]** (2 years at 5-7 years of age)	Home oxygen: 1,135*<br>No oxygen: 842*		Home oxygen: 1,135*<br>No oxygen: 842*	
**Greenough (2006)[22]** (2 years at 2-4 years of age**)**	Home oxygen: 5,556*<br>No oxygen: 1,728*		Home oxygen: 5,556*<br>No oxygen: 1,728*	
**Akman (2002)[21]**	see index hospitalization	21,392	-	15,116 (241%)
**Miller (1998)[20]**	34,692	25,268	5-year follow-up: 6,625<br>Indirect costs: 2800	
**McAleese (1993)[19]**	see index hospitalization	387,085	Home care, nurse: 133,427*<br>Home care, no nurse: 8,346*	
**Monset-Couchard (1984)[18]**	see index hospitalization	1,094,509		

Values are the mean costs, unless otherwise stated. *Median cost; Int$ = The cost after converting to purchasing power parities, then inflating to the 2019 value

Index hospitalization

The mean costs during the index hospitalization ranged from Int$21,392 to Int$1,094,509 (Figure 3).

For peer review only - http://bmjopen.bmj.com/site/about/guidelines.xhtml

For peer review only

Hospital readmissions in the first two years of life

One study analysed rehospitalizations among infants with BPD. They showed that rehospitalizations among infants with BPD occurred more than twice at frequently and incurred higher costs than rehospitalizations among their peers without BPD.[30] Rehospitalizations were required in 25.1% of infants with BPD, and the mean cost was estimated to be Int$46,948 during first year of life.[30] One study estimated that the cost associated with BPD for a mean follow-up of the first 2 years of life was Int$14,084.[26]

Follow-up costs and home care

Among children with BPD, the median costs during 2 years of follow-up for ages 2-4 were estimated at Int$5,556 for those that required home oxygen after the initial discharge, compared to Int$1,728 for those that did not require home oxygen after the initial discharge from the hospital.[22] Furthermore, the median costs during 2 years of follow-up for ages 5-7 were Int$1,135 for those that required home oxygen, compared to Int$842 for those that did not require home oxygen after the initial discharge.[23] Another study reported that the median follow-up cost of healthcare for children with BPD 4.5 years after initial hospitalisation was Int$6,625. Moreover, one study reported that the median cost of home healthcare was Int$133,427 with a nurse and Int$8,346 without a nurse.[20] One study analysed the cost of healthcare for children with BPD during a follow up for ages 16-25 years; they reported that the annual cost was Int$12,973 per person.[24]

Increase in costs due to BPD

In four of the included studies, BPD incurred, on average, 24 – 71% extra expenses in healthcare. One study from Canada reported that the mean follow-up cost increased by Int$2,651/year, which was a 24% increase over the cost for preterm peers without BPD.[24]

In four studies that reported costs according to GA categories, the cost ranges were: Int$196,713 – 507,638 for those born at or before GA 24 weeks/<750 g; Int$149,071 – 453,633 for those born at GA 25-26 weeks/750-999 g; and Int$73,237 – 351,027 for those born at GA 27-28 weeks/1000-1249 g. One study reported a cost of Int$313,223 for those born at GA 29 weeks or later/>1249 g (Figure 3). Higher cost increases were reported for infants with BPD born at higher GAs compared to those born at lower GAs. For example, studies reported that the mean costs for infants with BPD increased by 9 – 35% for those born at or before GA 24 weeks; by 15 – 35% for those born at GA 25-26 weeks; and by 19 – 35% for those born at GA 27-28 weeks. One study reported a 93% cost increase for infants born at GA 29 weeks or later. The mean total cost increase for infants with BPD, stratified by GA, are shown in Figure 4.

DISCUSSION

The included studies reported high costs for preterm infants with BPD during the initial hospitalization after birth (range: Int$21,392–1,094,509 per child, equivalent to €19,103–977,397, in 2019). Moreover, the studies reported a high use of resources associated with BPD during the following years, up to school age. The highest mean costs were associated with infants born at extremely low GAs.

The main strengths of this study were the systematic nature of the search and the assessments for eligibility by independent researchers. Several prior reviews and modelling studies in this field [14 32] have reported costs, but they based study inclusions on expert opinions and targeted searches. Therefore, the present study provided an overview of all the evidence available on costs for BPD. The main limitation of this study was the lack of searches for identifying grey literature, and the lack of a formal search, based on the references/citations in the included studies. However, we did perform an initial search based on references identified in, e.g., modelling studies and reviews, when we designed the search strategy, to ensure that we had identified all the relevant publications with our search strategy. We found several studies that addressed costs of interventions, such as immunization or infection treatment for infants with BPD, but in those cases, the costs were not included for BPD *per se*. Moreover, we initially intended to conduct a meta-analysis of results, but we found that, in the separate papers, the costs reported

For peer review only - http://bmjopen.bmj.com/site/about/guidelines.xhtml

differed too extensively in content and time-horizons to support that type of analysis. For the same reason, we could not conduct a formalized synthesis of the results. Moreover, the recently published guidelines for a data synthesis without a meta-analysis [33] were not suitable for this study; instead they were more suited to the synthesis of effect estimates for intervention studies. Consequently, the analysis herein was descriptive.

Another limitation that made a review difficult was that, in the BPD literature, the operational definition of BPD has varied since the condition was first discovered.[34] Thus, the extent of variability we encountered in the included papers (detailed in the Results section) might have affected the selection of infants, which might have influenced the healthcare costs. It is also important to note that the costs of BPD reported in the selected studies was based on the costs incurred by oxygen administration. However, the full economic impact of BPD should also include the costs associated with preventing BPD development in infants at risk. This same limitation applies to all retrospective studies that based the definition of BPD on ICD codes and clinical assessments, because infants that successfully avoided BPD development would not be classified as BPD, either with the codes or in clinical assessments.

Our search was tailored specifically to identify articles on BPD; thus, we excluded articles that used an alternate term, like chronic lung disease (CLD). We identified very few articles that were relevant, considering the great need of quality long-term healthcare for preterm infants with BPD. The majority of the included articles focused on the initial hospitalization. However, after the initial hospitalization, a considerable number of infants with BPD require rehospitalization during the first years of life, due to respiratory illnesses.[30] Infants born prematurely often display symptoms and signs of obstructive airway disease that persist for prolonged time periods; thus, it is important to implement standardized, regular follow-up schedules.[35]

Our findings showed how the cost estimates for BPD varied across different countries and during different time periods. Table 2 shows a large cost discrepancy among different countries, e.g., Turkey and the USA, which might be explained by several factors. First, BPD treatment programs might differ with different healthcare providers, during different years, and in different countries.[36] For example, articles that investigated the costs of BPD in the USA reported much higher costs than those reported in other countries; this discrepancy might partly be explained by higher cost of healthcare, in general, in the USA.[37] Second, in addition to the potential effects of different BPD definitions, infants with BPD frequently have other comorbidities, such as brain injury and retinopathy of prematurity (ROP), which can lead to functional impairments that clearly impact the cost of healthcare.[38] The cost of BPD without a comorbidity can be difficult to differentiate from the total cost including comorbidities. For example, the presence of comorbidities, like pulmonary hypertension (PH), in infants with BPD can significantly increase the healthcare costs, due to longer hospitalizations and a higher likelihood of requiring home respiratory support.[39] Third, the quality of healthcare can differ between different healthcare providers, which can also result in cost differences. Fourth, some of the included articles omitted data for some costs; e.g., the cost of physicians.[25] Finally, as previously discussed, an SGA condition can exacerbate the severity of BPD; this condition was not taken into account in the cost calculations, because the included articles did not report the combined effects of GA and BW.

Two studies compared healthcare costs between preterm infants with BPD and their preterm peers without BPD. Those data enabled a calculation of the cost increase due to BPD. The results clearly indicated that the cost differences were higher among infants born at lower GAs. This finding was consistent with cost estimates for other diagnoses in the same patient groups, such as infants born to mothers with preeclampsia.[28] Among our included studies, the study by Johnson et al [25] estimated marginal costs in infants with BPD compared to infants of the same weight without any of four major comorbidities (sepsis, brain injury, necrotizing enterocolitis [NEC], or BPD). They found that the cost increase was largest among the smallest infants (<750 g). Lapcharoensap et al [30] compared infants with BPD to all other infants of the same age (no limitation on comorbidities). They reported the opposite relationship, where larger cost differences were observed among those born at higher GAs. This relationship might be expected, because, in the reference groups, infants born at higher GAs can be expected to have fewer of the major comorbidities than infants born at lower GAs; thus, the high GA group would show a larger cost difference between infants with and without BPD. This effect was also reported by Mowitz et al.,[29] although the detailed cost data were not available. They reported that, among all children (regardless of comorbidity), the cost increase due to BPD was largest for infants born at a higher GA; however, when infants with BPD were compared to those without BPD, ROP, or intraventricular haemorrhage (IVH), the cost increase was similar independent of GA. In contrast, the

For peer review only - http://bmjopen.bmj.com/site/about/guidelines.xhtml

study by Johnson et al. showed a clear trend towards a higher cost difference due to BPD among the smallest infants. The differences between the two latter studies might be explained by the fact that the comorbidities excluded in the study by Johnson et al [25] (the comparison group had no sepsis, brain injury, NEC, or BPD) were different from those excluded in the study by Mowitz et al [29] (the comparison group had no ROP, IVH, or BPD).

It is clear from the included studies that infants and children with BPD often required long-term follow-ups. This requirement affects the life of these infants in various direct and indirect ways, which have not been properly investigated. A recent modelling study,[32] based on a targeted literature search, estimated the life-time burden of BPD for infants born ≤28 weeks GA. That study explored how BPD severity and major complications affected other aspects of life, like clinic visits for wheezing episodes, asthma exacerbations, psychiatric illnesses, et cetera. As expected, the model found that BPD had a large impact on healthcare use, quality-of-life, and survival, but it also showed that the degree of impact was largely dependent on BPD severity. Accordingly, improvements in BPD therapy might substantially reduce the impact of BPD.[32] However, as those authors acknowledged, the model could only use the available data, and thus, it was limited by, e.g., data on each complication separately (not accounting for concomitant diagnoses in one patient potentially increasing costs more than expected from single diagnosis studies). That study also reported that the healthcare cost of BPD for the first year of life was C$197,100, which was consistent with the estimates of more recent studies included in this review.

The findings from both our systematic review and the previous modelling study [32] suggested that there is a need for more research on the long-term economic impact of BPD. Preferably, those studies should also include costs that occur outside the healthcare system, such as costs due to changes in parent productivity and costs for schooling and day care. Moreover, studies should consider the costs related to the combined impact of complications. It has been argued that studies with a societal perspective can better identify potential inequities and inefficient use of society's resources.[40] One can speculate that one reason for the paucity of cost studies on BPD is that new drug developments have slowed after the success of surfactant therapy, which was introduced in the early 1990s.[41] Although several advances in the care of preterm infants and BPD have improved survival and symptom reduction,[42] these care applications and treatments have not, to the same extent, been subjected to economic and cost assessments.[43] To minimize the growing burden of BPD, it is vital to prevent prematurity, in the first place, and in the second place, to develop new treatments for prematurity-related diseases.[44]

This systematic review confirmed previous reports that BPD in preterm infants was associated with high costs and required long-term follow-ups. Our major finding was that the highest costs occurred among infants born at lower GAs, and that this relationship was difficult to detect, unless an appropriate control group was used. Moreover, we identified a shortage of studies that estimated, in particular, costs that occurred outside hospitals and after the first year of life. Further research on BPD is required to gain a proper understanding of the long-term consequences regarding healthcare costs and the social aspects of BPD.

For peer review only

For peer review only - http://bmjopen.bmj.com/site/about/guidelines.xhtml

FUNDING STATEMENT

JH was financed by the University of Gothenburg Centre for Person-Centred Care (GPCC) teaching assistant program, and the Mary von Sydow Foundation. CL was financed by the University of Gothenburg Centre for Person-Centred Care (GPCC). DL was financed by the Swedish Research Council (#2018-00770). AH was supported by the Swedish Research Council (#2016-01131), the Gothenburg Medical Society, De Blindas Vänner, and Government grants, under the ALF agreements (ALFGBG-717971 and ALFGBG-812951), and The Wallenberg Clinical Scholars. HG was financed by the Swedish Research Council (#2016-01131) and the Swedish Research Council (#2018-00770). The funders played no role in the design of the study or writing the protocol.

COMPETING INTERESTS STATEMENT

JH report no competing interests. CL hold stocks in Premalux AB. DL and AH hold stock/stock options in Premalux AB, and have received consulting fees from Takeda Inc. HG is employed part-time by Statfinn and EPID Research (part of IQVIA), which is a contract research organisation that performs commissioned pharmacoepidemiological studies and thus its employees have been and currently are working in collaboration with several pharmaceutical companies.

CONTRIBUTIONS

JH, CL, and HG designed the database search and data extraction methods. DL and AH contributed clinical expertise on preterm infants and morbidity. JH drafted the protocol. All authors contributed to the protocol and read and approved the final protocol.

ACKNOWLEDGEMENTS

This systematic review was part of the Less is More project (Swedish Research Council grant number #2018-00770), a longitudinal study of clinical and economic outcomes in children born at very low gestational age. This study thus contributes with an understanding of the current knowledge and important gaps to the Less is More study design for long-term follow-up and economic evaluation.

PATIENT AND PUBLIC INVOLVEMENT STATEMENT

This project did not include patient or public involvement in developing the research questions, design, conduct, choice of outcome measures or recruitment.

For peer review only - http://bmjopen.bmj.com/site/about/guidelines.xhtml

References

1. Hwang JS, Rehan VK. Recent Advances in Bronchopulmonary Dysplasia: Pathophysiology, Prevention, and Treatment. *Lung* 2018;**196**(2):129-38.
2. Northway WH, Jr., Rosan RC, Porter DY. Pulmonary disease following respirator therapy of hyaline-membrane disease. Bronchopulmonary dysplasia. *N Engl J Med* 1967;**276**(7):357-68.
3. Yeh TF, Lin HC, Chang CH, et al. Early intratracheal instillation of budesonide using surfactant as a vehicle to prevent chronic lung disease in preterm infants: a pilot study. *Pediatrics* 2008;**121**(5):e1310-8.
4. Tyson JE, Wright LL, Oh W, et al. Vitamin A supplementation for extremely-low-birth-weight infants. National Institute of Child Health and Human Development Neonatal Research Network. *N Engl J Med* 1999;**340**(25):1962-8.
5. Schmidt B, Roberts RS, Davis P, et al. Caffeine therapy for apnea of prematurity. *N Engl J Med* 2006;**354**(20):2112-21.
6. Bancalari E, del Moral T. Bronchopulmonary dysplasia and surfactant. *Biol Neonate* 2001;**80 Suppl 1**:7-13.
7. Rojas MA, Gonzalez A, Bancalari E, et al. Changing trends in the epidemiology and pathogenesis of neonatal chronic lung disease. *J Pediatr* 1995;**126**(4):605-10.
8. Coalson JJ. Pathology of new bronchopulmonary dysplasia. *Semin Neonatol* 2003;**8**(1):73-81.
9. Norman M, Hallberg B, Abrahamsson T, et al. Association Between Year of Birth and 1-Year Survival Among Extremely Preterm Infants in Sweden During 2004-2007 and 2014-2016. *JAMA* 2019;**321**(12):1188-99.
10. Qiu X, Lodha A, Shah PS, et al. Neonatal outcomes of small for gestational age preterm infants in Canada. *Am J Perinatol* 2012;**29**(2):87-94.
11. Ehrenkranz RA, Walsh MC, Vohr BR, et al. Validation of the National Institutes of Health consensus definition of bronchopulmonary dysplasia. *Pediatrics* 2005;**116**(6):1353-60.
12. Blencowe H, Cousens S, Oestergaard MZ, et al. National, regional, and worldwide estimates of preterm birth rates in the year 2010 with time trends since 1990 for selected countries: a systematic analysis and implications. *Lancet* 2012;**379**(9832):2162-72.
13. Bhandari A, McGrath-Morrow S. Long-term pulmonary outcomes of patients with bronchopulmonary dysplasia. *Semin Perinatol* 2013;**37**(2):132-7.
14. Lapcharoensap W, Lee H, Nyberg A, et al. Health Care and Societal Costs of Bronchopulmonary Dysplasia. *NeoReviews* 2018;**19**:e211-e23.
15. Evers S, Goossens M, de Vet H, et al. Criteria list for assessment of methodological quality of economic evaluations: Consensus on Health Economic Criteria. *Int J Technol Assess Health Care* 2005;**21**(2):240-5.
16. OECD. Purchasing power parities (PPP) (indicator). Secondary Purchasing power parities (PPP) (indicator) 2020. https://data.oecd.org/conversion/purchasing-power-parities-ppp.htm.
17. OECD. Inflation (CPI) (Indicator). Secondary Inflation (CPI) (Indicator) 2020. https://data.oecd.org/price/inflation-cpi.htm.
18. Monset-Couchard M, Jaspar ML, de Bethmann O, et al. [Costs of the initial care of children with birth weight less than or equal to 1500 g in 1981]. *Arch Fr Pediatr* 1984;**41**(8):579-85.
19. McAleese KA, Knapp MA, Rhodes TT. Financial and emotional cost of bronchopulmonary dysplasia. *Clin Pediatr (Phila)* 1993;**32**(7):393-400.
20. Miller VL, Rice JC, DeVoe M, et al. An analysis of program and family costs of case managed care for technology-dependent infants with bronchopulmonary dysplasia. *J Pediatr Nurs* 1998;**13**(4):244-51.
21. Akman Y, Cebecý D, Özek E, et al. Effects of perinatal factors on the duration and cost of hospitalisation for preterm infants in a neonatal intensive care unit in Istanbul. *Turkish Journal of Medical Sciences* 2002;**32**:159-63.
22. Greenough A, Alexander J, Burgess S, et al. Preschool healthcare utilisation related to home oxygen status. *Arch Dis Child Fetal Neonatal Ed* 2006;**91**(5):F337-41.
23. Greenough A, Alexander J, Boorman J, et al. Respiratory morbidity, healthcare utilisation and cost of care at school age related to home oxygen status. *Eur J Pediatr* 2011;**170**(8):969-75.
24. Landry JS, Croitoru D, Jin Y, et al. Health care utilization by preterm infants with respiratory complications in Quebec. *Can Respir J* 2012;**19**(4):255-60.
25. Johnson TJ, Patel AL, Jegier BJ, et al. Cost of morbidities in very low birth weight infants. *J Pediatr* 2013;**162**(2):243-49 e1.
26. Alvarez-Fuente M, Arruza L, Muro M, et al. The economic impact of prematurity and bronchopulmonary dysplasia. *Eur J Pediatr* 2017;**176**(12):1587-93.

For peer review only - http://bmjopen.bmj.com/site/about/guidelines.xhtml

27. Patel AL, Johnson TJ, Robin B, et al. Influence of own mother's milk on bronchopulmonary dysplasia and costs. *Arch Dis Child Fetal Neonatal Ed* 2017;**102**(3):F256-F61.
28. Stevens W, Shih T, Incerti D, et al. Short-term costs of preeclampsia to the United States health care system. *Am J Obstet Gynecol* 2017;**217**(3):237-48 e16.
29. Mowitz ME, Ayyagari R, Gao W, et al. Health Care Burden of Bronchopulmonary Dysplasia Among Extremely Preterm Infants. *Front Pediatr* 2019;**7**:510.
30. Lapcharoensap W, Bennett MV, Xu X, et al. Hospitalization costs associated with bronchopulmonary dysplasia in the first year of life. *J Perinatol* 2020;**40**(1):130-37.
31. Jobe AH, Bancalari E. Bronchopulmonary dysplasia. *Am J Respir Crit Care Med* 2001;**163**(7):1723-9.
32. van Katwyk S, Augustine S, Thebaud B, et al. Lifetime patient outcomes and healthcare utilization for Bronchopulmonary dysplasia (BPD) and extreme preterm infants: a microsimulation study. *BMC Pediatr* 2020;**20**(1):136.
33. Campbell M, McKenzie JE, Sowden A, et al. Synthesis without meta-analysis (SWiM) in systematic reviews: reporting guideline. *BMJ* 2020;**368**:l6890.
34. Thebaud B, Goss KN, Laughon M, et al. Bronchopulmonary dysplasia. *Nat Rev Dis Primers* 2019;**5**(1):78.
35. Landry JS, Chan T, Lands L, et al. Long-term impact of bronchopulmonary dysplasia on pulmonary function. *Can Respir J* 2011;**18**(5):265-70.
36. Williams I, Brown H, Healy P. Contextual Factors Influencing Cost and Quality Decisions in Health and Care: A Structured Evidence Review and Narrative Synthesis. *Int J Health Policy Manag* 2018;**7**(8):683-95.
37. Ridic G, Gleason S, Ridic O. Comparisons of health care systems in the United States, Germany and Canada. *Mater Sociomed* 2012;**24**(2):112-20.
38. Schmidt B, Roberts RS, Davis PG, et al. Prediction of Late Death or Disability at Age 5 Years Using a Count of 3 Neonatal Morbidities in Very Low Birth Weight Infants. *J Pediatr* 2015;**167**(5):982-6 e2.
39. Stuart BD, Sekar P, Coulson JD, et al. Health-care utilization and respiratory morbidities in preterm infants with pulmonary hypertension. *J Perinatol* 2013;**33**(7):543-7.
40. Johannesson M, Jonsson, B., Jonsson, L., Kobelt, G., & Zethraeus, N. Why Should Economic Evaluations of Medical Innovations Have a Societal Perspective? *Office of health economics (OHE)* 2009(51, October 2009).
41. Curstedt T, Halliday HL, Speer CP. A unique story in neonatal research: the development of a porcine surfactant. *Neonatology* 2015;**107**(4):321-9.
42. Principi N, Di Pietro GM, Esposito S. Bronchopulmonary dysplasia: clinical aspects and preventive and therapeutic strategies. *J Transl Med* 2018;**16**(1):36.
43. Zupancic JAF. A Systematic Review of Costs Associated with Preterm Birth. In: and CoUPB, Outcomes AH, eds. Preterm Birth: Causes, Consequences, and

Prevention National Academies Press (US), 2007:688 - 724.
44. Davidson LM, Berkelhamer SK. Bronchopulmonary Dysplasia: Chronic Lung Disease of Infancy and Long-Term Pulmonary Outcomes. *J Clin Med* 2017;**6**(1).

For peer review only

For peer review only - http://bmjopen.bmj.com/site/about/guidelines.xhtml

FIGURE LEGENDS

Figure 1: Flow diagram shows the study selection process, in accordance with the PRISMA guidelines. Abbreviations: BPD = Bronchopulmonary Dysplasia

Figure 2: Overview of the costs of BPD and the study period included in each article. The numbers on the Y-axis refer to the numbers assigned to each study (Table 1).

Figure 3: Costs of the index hospitalizations for preterm infants with BPD in studies conducted between 1981 and 2015. All costs were converted to Int$, based on purchasing power parities, then the values were inflated to 2019 values. Abbreviations: BPD = Bronchopulmonary Dysplasia; control infants = preterm infants without BPD; mInt$ = millions of Int$.

Figure 4: Mean cost increase for infants with BPD, stratified by gestational age (GA) at birth. The numbers on the x-axis refer to the numbers assigned to studies in Table 1. Abbreviations: BPD = Bronchopulmonary dysplasia; Int$ = The cost after converting to purchasing power parities, then inflating to the 2019 value; mInt$ = millions of Int$, w = weeks

For peer review only

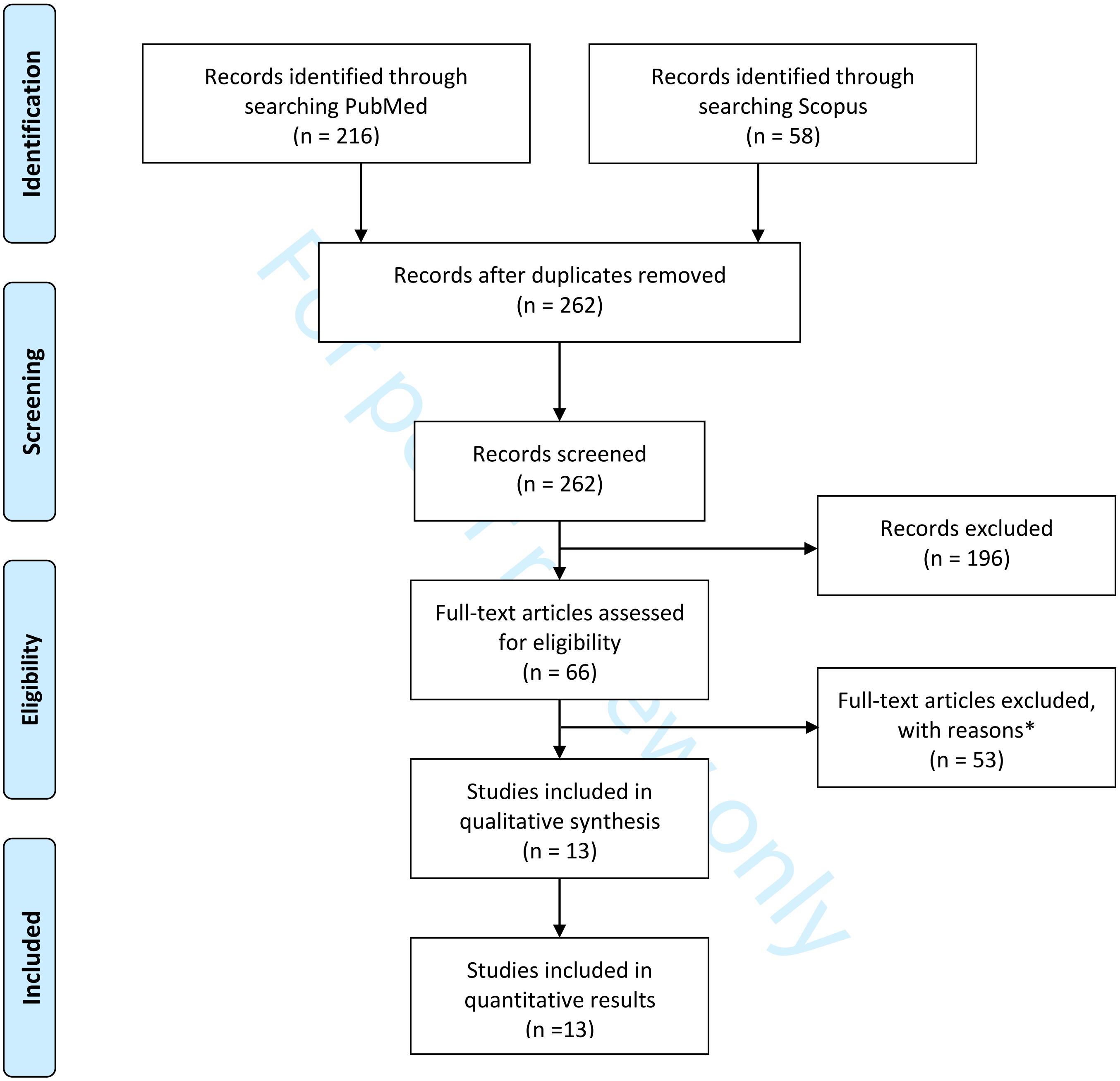

* Reasons for exclusion after full text assessment:

- Cost data not specific to BPD (n = 26)
- Only billed charges, no detailed cost estimates (n = 2)
- No cost analysis with original data (n = 25)

For peer review only - http://bmjopen.bmj.com/site/about/guidelines.xhtml

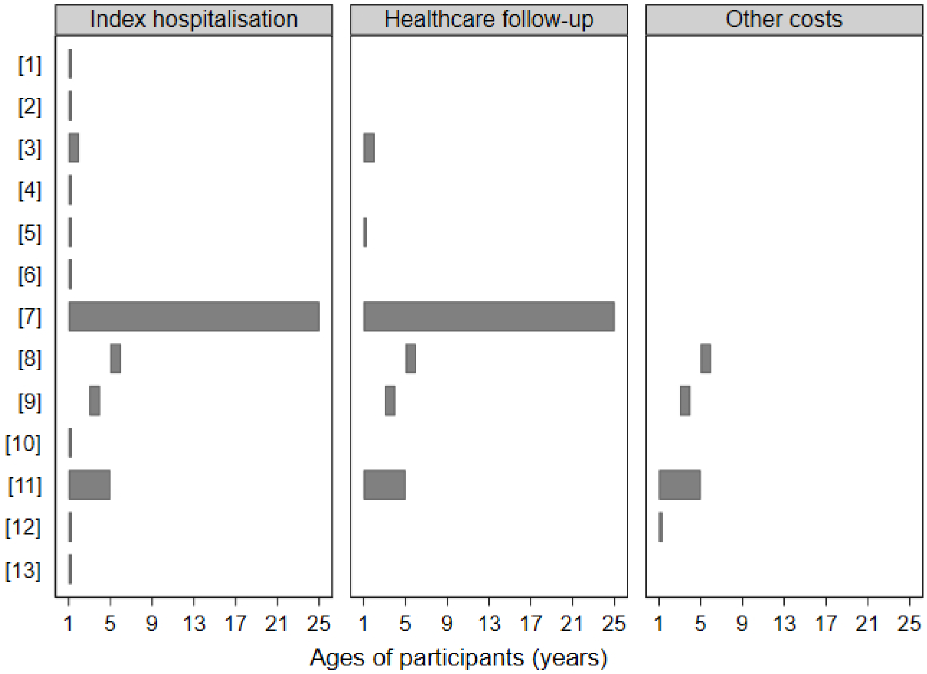

Figure 2
Overview of the costs of BPD and the study period included in each article. The numbers on the Y-axis refer to the numbers assigned to each study (Table 1).

244x177mm (72 x 72 DPI)

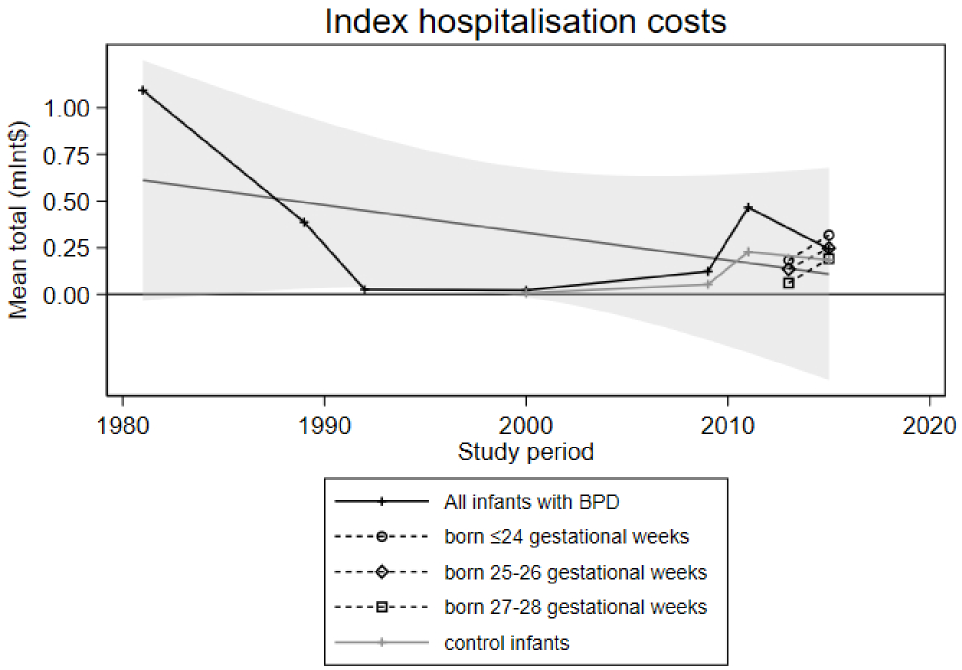

Figure 3
Costs of the index hospitalizations for preterm infants with BPD in studies conducted between 1981 and 2015. All costs were converted to Int$, based on purchasing power parities, then the values were inflated to 2019 values. Abbreviations: BPD = Bronchopulmonary Dysplasia; control infants = preterm infants without BPD; mInt$ = millions of Int$.

244x177mm (72 x 72 DPI)

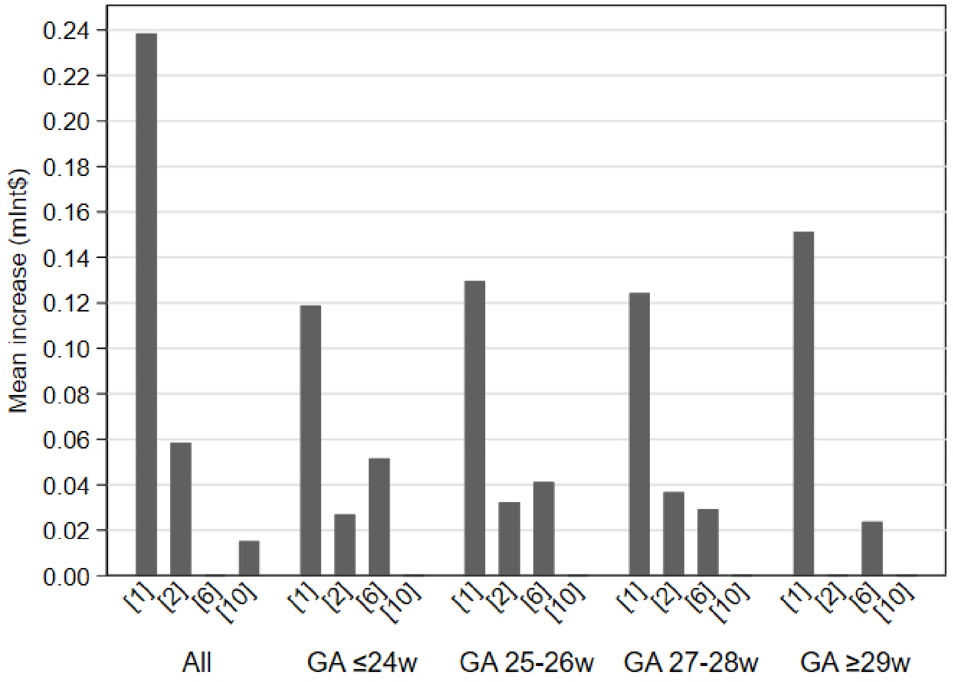

Figure 4
Mean cost increase for infants with BPD, stratified by gestational age (GA) at birth. The numbers on the x-axis refer to the numbers assigned to studies in Table 1. Abbreviations: BPD = Bronchopulmonary dysplasia; Int$ = The cost after converting to purchasing power parities, then inflating to the 2019 value; mInt$ = millions of Int$, w = weeks

244x177mm (72 x 72 DPI)

SUPPLEMENTAL MATERIAL

SYSTEMATIC REVIEW OF THE HEALTHCARE COST OF BRONCHOPULMONARY DYSPLASIA

AUTHORS

Jhangir Humayun[1,2], gushumjh@student.gu.se

Chatarina Löfqvist[1,2,3] chatarina.lofqvist@gu.se

David Ley[4], david.ley@med.lu.se

Ann Hellström[3], ann.hellstrom@medfak.gu.se

Hanna Gyllensten[1,2] hanna.gyllensten@gu.se

Author affiliations

[1] Institute of Health and Care Sciences, Sahlgrenska Academy, University of Gothenburg, Box 457, SE-405 30 Gothenburg, Sweden.

[2] Centre for Person-Centred Care (GPCC), University of Gothenburg, Box 457, SE-405 30 Gothenburg, Sweden.

[3] Department of Clinical Neuroscience, Institute of Neuroscience and Physiology, Sahlgrenska Academy, University of Gothenburg, Gothenburg, Sweden;

[4] Department of Pediatrics, Institute of Clinical Sciences Lund, Lund University and Skåne University Hospital, Lund, Sweden

Table S1: Search terms

Database	Search string
PubMed	((("Bronchopulmonary Dysplasia"[Title/Abstract]) OR "Bronchopulmonary Dysplasia"[Mesh])) AND (("Economics"[Mesh]) OR ((economic*[Title/Abstract] OR cost[Title/Abstract] OR costs[Title/Abstract] OR costly[Title/Abstract] OR costing[Title/Abstract] OR price[Title/Abstract] OR prices[Title/Abstract] OR pricing[Title/Abstract] OR pharmacoeconomic*[Title/Abstract])))
Scopus	(TITLE-ABS-KEY ("Bronchopulmonary Dysplasia")) AND (TITLE-ABS-KEY (economic* OR cost OR costs OR costly OR costing OR price OR prices OR pricing OR pharmacoeconomic*))

For peer review only

Table S2: Checklist for the quality appraisal of included papers (from [15])

Checklist items	21	30	29	26	28	27	25	24	23	22	20	19	18
Is the study population clearly described?	-	+	+	-	+	+	+	+	+	+	-	+	-
Are competing alternatives clearly described?	NA	NA	NA	NA	NA	+	NA	NA	+	+	NA	NA	NA
Is a well-defined research question posed in answerable form?	-	-	-	-	-	-	-	-	-	-	-	-	-
Is the economic study design appropriate to the stated objective?	+	+	+	+	+	+	+	+	+	+	+	+	+
Is the chosen time horizon appropriate in order to include relevant costs and consequences?	NA	NA	NA	NA	NA	NA	NA	NA	NA	NA	NA	NA	NA
Is the actual perspective chosen appropriate?	+	+	+	+	+	+	+	+	+	+	+	+	+
Are all important and relevant costs for each alternative identified?	+	+	+	+	+	+	-	+	+	+	+	+	+
Are all costs measured appropriately in physical units?	+	+	+	+	+	+	+	-	+	+	+	+	+
Are costs valued appropriately?	+	+	+	+	+	+	+	+	+	+	+	+	+
Are all important and relevant outcomes for each alternative identified?	NA	NA	NA	NA	NA	NA	NA	NA	NA	NA	NA	NA	NA
Are all outcomes measured appropriately?	NA	NA	NA	A	NA	NA	NA	NA	NA	NA	NA	NA	NA
Are outcomes valued appropriately?	NA	NA	NA	NA	NA	NA	NA	NA	NA	NA	NA	NA	NA
Is an incremental analysis of costs and outcomes of alternatives performed?	NA	NA	NA	NA	NA	NA	NA	NA	NA	NA	NA	NA	NA
Are all future costs and outcomes discounted appropriately?	NA	NA	NA	NA	+	NA	+	+	+	+	-	+	+
Are all important variables, whose values are uncertain, appropriately subjected to sensitivity analysis?	NA	-	-	-	+	-	-	-	-	-	-	-	-
Do the conclusions follow from the data reported?	-	+	+	+	+	+	+	+	+	+	+	+	+
Does the study discuss the generalizability of the results to other settings and patient/client groups?	-	+	+	+	+	+	+	+	+	+	+	+	+
Does the article indicate that there is no potential conflict of interest of study researcher(s) and funder(s)?	-	+	+	+	+	+	+	+	+	+	-	-	-
Are ethical and distributional issues discussed appropriately?	+	+	+	+	+	+	+	+	+	+	+	+	+
Number of checklist items fulfilled by each individual study	6	10	10	9	12	11	10	10	12	12	8	10	9

+ indicates a fulfilled checklist item, - indicates that the item was either not stated or not fulfilled, NA indicates that the checklist item was not relevant for that study

* Number of studies that fulfilled each checklist item (not considering the applicability of the checklist item to the individual studies

For peer review only

Section/topic	#	PRISMA checklist item	Reported on page #
**TITLE**			
Title	1	Identify the report as a systematic review, meta-analysis, or both.	1
**ABSTRACT**			
Structured summary	2	Provide a structured summary including, as applicable: background; objectives; data sources; study eligibility criteria, participants, and interventions; study appraisal and synthesis methods; results; limitations; conclusions and implications of key findings; systematic review registration number.	2
**INTRODUCTION**			
Rationale	3	Describe the rationale for the review in the context of what is already known.	4
Objectives	4	Provide an explicit statement of questions being addressed with reference to participants, interventions, comparisons, outcomes, and study design (PICOS).	5
**METHODS**			
Protocol and registration	5	Indicate if a review protocol exists, if and where it can be accessed (e.g., Web address), and, if available, provide registration information including registration number.	6
Eligibility criteria	6	Specify study characteristics (e.g., PICOS, length of follow-up) and report characteristics (e.g., years considered, language, publication status) used as criteria for eligibility, giving rationale.	6
Information sources	7	Describe all information sources (e.g., databases with dates of coverage, contact with study authors to identify additional studies) in the search and date last searched.	6
Search	8	Present full electronic search strategy for at least one database, including any limits used, such that it could be repeated.	Table S1
Study selection	9	State the process for selecting studies (i.e., screening, eligibility, included in systematic review, and, if applicable, included in the meta-analysis).	6
Data collection process	10	Describe method of data extraction from reports (e.g., piloted forms, independently, in duplicate) and any processes for obtaining and confirming data from investigators.	6-7
Data items	11	List and define all variables for which data were sought (e.g., PICOS, funding sources) and any assumptions and simplifications made.	7
Risk of bias in individual studies	12	Describe methods used for assessing risk of bias of individual studies (including specification of whether this was done at the study or outcome level), and how this information is to be used in any data synthesis.	7
Summary measures	13	State the principal summary measures (e.g., risk ratio, difference in means).	7
Synthesis of results	14	Describe the methods of handling data and combining results of studies, if done, including measures of consistency (e.g., I^2) for each meta-analysis.	7

For peer review only

Section/topic	#	PRISMA checklist item	Reported on page #
Risk of bias across studies	15	Specify any assessment of risk of bias that may affect the cumulative evidence (e.g., publication bias, selective reporting within studies).	NA
Additional analyses	16	Describe methods of additional analyses (e.g., sensitivity or subgroup analyses, meta-regression), if done, indicating which were pre-specified.	7
**RESULTS**			
Study selection	17	Give numbers of studies screened, assessed for eligibility, and included in the review, with reasons for exclusions at each stage, ideally with a flow diagram.	7<br>Figure 1
Study characteristics	18	For each study, present characteristics for which data were extracted (e.g., study size, PICOS, follow-up period) and provide the citations.	Table 1
Risk of bias within studies	19	Present data on risk of bias of each study and, if available, any outcome level assessment (see item 12).	10<br>Table S2
Results of individual studies	20	For all outcomes considered (benefits or harms), present, for each study: (a) simple summary data for each intervention group (b) effect estimates and confidence intervals, ideally with a forest plot.	Table 2
Synthesis of results	21	Present results of each meta-analysis done, including confidence intervals and measures of consistency.	NA
Risk of bias across studies	22	Present results of any assessment of risk of bias across studies (see Item 15).	10
Additional analysis	23	Give results of additional analyses, if done (e.g., sensitivity or subgroup analyses, meta-regression [see Item 16]).	12
**DISCUSSION**			
Summary of evidence	24	Summarize the main findings including the strength of evidence for each main outcome; consider their relevance to key groups (e.g., healthcare providers, users, and policy makers).	12
Limitations	25	Discuss limitations at study and outcome level (e.g., risk of bias), and at review-level (e.g., incomplete retrieval of identified research, reporting bias).	13
Conclusions	26	Provide a general interpretation of the results in the context of other evidence, and implications for future research.	13
**FUNDING**			
Funding	27	Describe sources of funding for the systematic review and other support (e.g., supply of data); role of funders for the systematic review.	14

Abbreviations: NA = Not applicable.

For peer review only

BMJ Open

SYSTEMATIC REVIEW OF THE HEALTHCARE COST OF BRONCHOPULMONARY DYSPLASIA

Journal:	*BMJ Open*
Manuscript ID	bmjopen-2020-045729.R1
Article Type:	Original research
Date Submitted by the Author:	22-Feb-2021
Complete List of Authors:	Humayun, Jhangir; University of Gothenburg, Institute of Health and Care Sciences; University of Gothenburg Centre for Person-Centred Care<br>Löfqvist, Chatarina; University of Gothenburg Sahlgrenska Academy, Institute of Health and Care Sciences; University of Gothenburg Institute of Neuroscience and Physiology, Department of Clinical Neuroscience<br>Ley, David; Lund University, Department of Pediatrics, Institute of Clinical Sciences and Skåne University Hospital<br>Hellström, Ann; University of Gothenburg Institute of Neuroscience and Physiology, Department of Clinical Neuroscience<br>Gyllensten, Hanna; University of Gothenburg, Institute of Health and Care Sciences; University of Gothenburg Centre for Person-Centred Care,
**Primary Subject Heading**:	Health economics
Secondary Subject Heading:	Paediatrics, Intensive care
Keywords:	HEALTH ECONOMICS, PAEDIATRICS, RESPIRATORY MEDICINE (see Thoracic Medicine), Chronic airways disease < THORACIC MEDICINE, Paediatric thoracic medicine < THORACIC MEDICINE

SCHOLARONE™
Manuscripts

I, the Submitting Author has the right to grant and does grant on behalf of all authors of the Work (as defined in the below author licence), an exclusive licence and/or a non-exclusive licence for contributions from authors who are: i) UK Crown employees; ii) where BMJ has agreed a CC-BY licence shall apply, and/or iii) in accordance with the terms applicable for US Federal Government officers or employees acting as part of their official duties; on a worldwide, perpetual, irrevocable, royalty-free basis to BMJ Publishing Group Ltd ("BMJ") its licensees and where the relevant Journal is co-owned by BMJ to the co-owners of the Journal, to publish the Work in this journal and any other BMJ products and to exploit all rights, as set out in our licence.

The Submitting Author accepts and understands that any supply made under these terms is made by BMJ to the Submitting Author unless you are acting as an employee on behalf of your employer or a postgraduate student of an affiliated institution which is paying any applicable article publishing charge ("APC") for Open Access articles. Where the Submitting Author wishes to make the Work available on an Open Access basis (and intends to pay the relevant APC), the terms of reuse of such Open Access shall be governed by a Creative Commons licence – details of these licences and which Creative Commons licence will apply to this Work are set out in our licence referred to above.

Other than as permitted in any relevant BMJ Author's Self Archiving Policies, I confirm this Work has not been accepted for publication elsewhere, is not being considered for publication elsewhere and does not duplicate material already published. I confirm all authors consent to publication of this Work and authorise the granting of this licence.

For peer review only

SYSTEMATIC REVIEW OF THE HEALTHCARE COST OF BRONCHOPULMONARY DYSPLASIA

AUTHORS

Jhangir Humayun[1,2], gushumjh@student.gu.se

Chatarina Löfqvist[1,2,3] chatarina.lofqvist@gu.se

David Ley[4], david.ley@med.lu.se

Ann Hellström[3], ann.hellstrom@medfak.gu.se

Hanna Gyllensten[1,2] hanna.gyllensten@gu.se

Author affiliations

[1] Institute of Health and Care Sciences, Sahlgrenska Academy, University of Gothenburg, Box 457, SE-405 30 Gothenburg, Sweden.

[2] Centre for Person-Centred Care (GPCC), University of Gothenburg, Box 457, SE-405 30 Gothenburg, Sweden.

[3] Department of Clinical Neuroscience, Institute of Neuroscience and Physiology, Sahlgrenska Academy, University of Gothenburg, Gothenburg, Sweden.

[4] Department of Pediatrics, Institute of Clinical Sciences Lund, Lund University and Skåne University Hospital, Lund, Sweden.

Contact, corresponding author

Hanna Gyllensten, Institute of Health and Care Sciences, Sahlgrenska Academy, University of Gothenburg, Postal Address: Box 457, SE-405 30 Gothenburg, Sweden, E-mail: hanna.gyllensten@gu.se. Phone: +46-(0)70-748 24 12

Word count (excluding title page, abstract, references, figures and tables): 4375

For peer review only

ABSTRACT

Objectives

To determine the costs directly or indirectly related to bronchopulmonary dysplasia (BPD) in preterm infants. The secondary objective was to stratify the costs, based on gestational age and/or birth weight.

Design

Systematic literature review

Setting

PubMed and Scopus were searched on 3 Feb 2020. Studies were selected based on eligibility criteria by two independent reviewers. Included studies were further searched to identify eligible references and citations.

Data were extracted by two independent reviewers with a pre-specified data extraction sheet, including items from a published checklist for quality assessment. The costs in the included studies are reported descriptively.

Primary outcome measure

Costs of BPD.

Results

The 13 included studies reported the total costs or marginal costs of BPD. A majority of studies reported costs during birth hospitalization (cost range: Int$21,392–1,094,509 per child, equivalent to €19,103–977,397, in 2019) and/or during the first year of life. One study reported costs during the first two years; two other studies reported costs later, during the pre-school period; and one study included a long-term follow up. The highest mean costs were associated with infants born at extremely low gestational ages. Quality assessment indicated low risk of bias in the reported findings of included studies.

Conclusions

This study was the first systematic review of costs associated with BPD. We confirmed previous reports of high costs and described the long-term follow-up necessary for preterm infants with BPD, particularly infants of very low gestational age. Moreover, we identified a need for studies that estimate costs that occur outside of hospitals and after the first year of life.

Registration

The protocol is registered in PROSPERO (registration number CRD42020173234).

For peer review only - http://bmjopen.bmj.com/site/about/guidelines.xhtml

ARTICLE SUMMARY

Strengths and limitations of this study

- This is the first systematic review specifically focused on costs resulting from bronchopulmonary dysplasia after preterm birth.
- Authors searched systematically through PubMed and Scopus, which are appropriate databases for the investigation topic, and also reviewed reference lists and citations of the relevant papers to identify any additional studies.
- The main limitation was the lack of search for unpublished and/or grey literature.

For peer review only

For peer review only - http://bmjopen.bmj.com/site/about/guidelines.xhtml

INTRODUCTION

Bronchopulmonary dysplasia (BPD) is a serious lung condition that can affect new-borns. It is particularly common in premature infants, particularly infants that require oxygen therapy. Infants born more than 10 weeks preterm are more susceptible to BPD, because they often require a long treatment period with different types of breathing support, such as nasal continuous positive airway pressure, ventilators, different bronchodilators, and steroids.[1]

The definition of BPD has varied considerably over time. The condition was first described by Northway et al., in 1967, as a radiographic pattern of lung injury observed in prematurely born infants after the use of extensive mechanical ventilation.[2] Considerable strides in care have been made for these infants over the past decades, including, but not limited to, the use of antenatal steroids, surfactant therapy, vitamin A supplementation, and caffeine treatment.[3-5] The administration of antenatal steroids and exogenous surfactant has greatly reduced the severity of respiratory distress syndrome, and consequently, increased the survival of preterm infants.[6] Prior to the widespread use of antenatal steroids and surfactant, BPD in preterm infants was mainly attributed to lung injury caused by barotrauma and oxygen toxicity.[6] The pathogenesis of the BPD observed currently, in the post surfactant era, is multifactorial; various causes have been found, including extreme prematurity, infection, nutritional deprivation, and the need for prolonged mechanical ventilation.[7 8] Short-term management thus includes, preferably non-invasive, respiratory support titrated to certain oxygen saturation ranges, exogenous surfactant therapy, respiratory stimulants such as caffeine, corticosteroids to reduce inflammation, and inhaled nitric oxide against pulmonary hypertension, while long-term management includes a larger range of products to handle persistent and severe chronic respiratory disease and related comorbidities.[9] It is imperative to understand this new form of BPD and its effects during infancy and beyond, because the survival of preterm infants is increasing.[10]

Currently, BPD is commonly defined as a condition that requires oxygen administration beyond 36 weeks postmenstrual age (PMA). Depending on the required respiratory support and the concentration of inhaled oxygen, BPD severity is classified as light, moderate, or severe. Co-morbidities and maternal infections increase the risk of developing serious BPD after a preterm birth. Disease severity varies considerably; for example, in mild cases, infants might only present with an accelerated respiratory rate; but in severe cases, mechanical ventilation may be required for a prolonged period of time.[1] Indeed, serious conditions often require continuous oxygen therapy for several months.

The incidence of BPD seems to be inversely related to the gestational age (GA, reported in weeks) at birth. Studies that included infants born at low GAs have reported higher incidences of BPD.[11] The National Institute for Child Health and Human Development neonatal network periodically reports the incidence of BPD. They define BPD as a condition that requires oxygen administration at 36 weeks PMA. They reported BPD incidences of 52% in infants with birth weights of 501 - 750 g, 34% in infants with birth weights of 751-1000 g, and 15% in infants with birth weights of 1001-1200 g. Among infants that weighed 1201-1500 g, the incidence was as low as 7%.[12]

Foetal growth restriction is commonly defined as a low birth weight, corrected for GA. Foetal growth restriction has a clear impact on the incidence and severity of BPD. Studies have shown that, among infants born at a similar GA, infants that were small for their GA (SGA) had a higher BPD incidence than infants that had normal birth weights for their GA.[11] Globally, the survival rates of extremely preterm infants (GA <28 weeks) have varied considerably, according to the economic setting. Highly

For peer review only - http://bmjopen.bmj.com/site/about/guidelines.xhtml

developed countries have reported extremely preterm infant survival rates as high as ~90%, but in low-income settings, it can be as low as ~10%.[13] Similarly, the incidence of BPD in preterm infants varies considerably with the degree of economic development in the country of birth.

The effects of BPD on infant health extend beyond the initial hospitalization. Infants with BPD are hospitalized to a much greater extent than infants without BPD.[14] Lung function abnormalities, such as small airways, often persist for a long period of time. Long-term effects, such as reduced lung function and obstructive airway disease, are more prevalent among individuals that were born prematurely than among their peers that were born full-term. Among preterm infants, those with BPD exhibit a greater need for respiratory medications, compared to those without BPD.[14] Neurodevelopment can be severely affected by BPD. Infants with BPD have an increased rate of abnormal neurodevelopment, including cognitive impairments and cerebral palsy. In severe cases of BPD, movement morbidities can occur, like chorea and akathisia.[15]

An economic evaluation is important for aiding decision-making bodies to prioritize healthcare funding effectively and understand the associated long-term costs. A review [15] was previously published on the healthcare and societal cost of BPD, but the search was not systematic; all the articles were handpicked by the authors. To our knowledge, no systematic review has been published that discussed the healthcare costs of BPD. Thus, there was a need to compile and present the available data on this subject in the form of a systematic review.

The present review aimed to present all available data that pertained to the costs related to BPD, directly or indirectly, in preterm infants. The secondary objective was to stratify those costs, based on the GA or birth weight of the infant.

METHODS

This study was conducted according to the Preferred Reporting Items for Systematic Review and Meta-Analysis (PRISMA) recommendations.[5] The protocol is registered in PROSPERO (registration number CRD42020173234).

Search strategy

In collaboration with personnel of the Medicine Library at the University of Gothenburg, the authors determined that the PubMed and Scopus databases were the best choice for this review. The search was conducted on 3 Feb 2020 in both databases. Search terms (Appendix, Table S1) were drafted and discussed individually for both databases. The search was performed, and articles were included according to the criteria listed below. No restrictions were applied for publication date, country of origin, or language. Included studies were further searched to identify eligible references and citations.

Study selection

We included observational and interventional studies that investigated the costs associated with BPD. The study population comprised preterm human infants with BPD. Two reviewers (JH and HG) independently screened the titles and abstracts of identified studies to assess their eligibility for inclusion. After reviewing the titles and abstracts, duplicates were removed. Then, the reviewers obtained full texts of potentially eligible articles and assessed them independently to determine

For peer review only - http://bmjopen.bmj.com/site/about/guidelines.xhtml

which articles met the predetermined inclusion criteria. When the reviewers were uncertain of the eligibility of a study, a third reviewer (CL) participated in the decision.

Data extraction and quality assessment

To ensure important data were extracted consistently from relevant articles, we created an extraction table. Data from eligible studies were independently extracted by two reviewers (JH and HG). Any differences were resolved by discussion and consultation with a third reviewer (CL). The predesigned data extraction table included, but was not limited to the following data: the name of first author, year of publication, study design, location, sample size, healthcare costs, and description of the costs included. When available, healthcare costs were stratified according to the GA at birth or birth weight. The two authors independently assessed the quality of the selected studies, based on predetermined quality assessment criteria for health economic studies (the CHEC-list) described previously by Evers et al.[16] Although designed for economic evaluations, this tool has previously been used in similar studies.[17 18]

Analyses

Costs were translated to USD, based on the purchasing power parities of each year.[19] Then, the costs were inflated to 2019 values with the Organisation for Economic Co-operation and Development (OECD) inflation factor.[20] Costs were analysed, based on the age of the participants included in each study and by their GA or birth weight.

Patient and public involvement

This project did not include patient or public involvement in developing the research questions, design, conduct, choice of outcome measures or recruitment.

RESULTS

The initial search results yielded 274 articles. After removing duplicates (Figure 1), 262 unique articles were screened, and 195 were excluded based on the abstract or title. Among the 66 full articles screened, 13 [21-33] unique studies met the inclusion criteria. The hand search of references and citations of included studies did not identify any additional eligible studies. The included studies were conducted in 1981-2015 (Table 1), in the US (7 studies), UK (2 studies), Spain, Canada, Turkey, and France.

Six studies defined BPD as oxygen dependence beyond 28 days after birth (table 1), and some of these studies also classified BPD as mild/moderate/severe, based on whether oxygen was administered at 36 weeks PMA, which, according to Jobe and Bancalari,[34] indicated moderate/severe BPD. Three studies used only the 36 weeks PMA oxygen use definition, while three additional studies defined BPD based on the ICD.9 (770.7) code from a registry, without reference to GA or time since birth at diagnosis. In the oldest included study,[21] the definition was based on hospital data, but they did not state whether they used ICD codes or what time-period they used for the oxygen requirement. However, their definition was consistent with the classical definition of BPD,[2] which was based on clinical signs. In two studies, the definition of BPD required a chest x-ray consistent with BPD, in addition to the oxygen dependence beyond 28 days after birth requirement.[22 23]

For peer review only - http://bmjopen.bmj.com/site/about/guidelines.xhtml

Table 1: Overview of studies included in this review

#	First author (year)	Country (study period) Setting	Study design	BPD definition	Sample size	Time horizon: age of children	Healthcare cost per child with BPD	Cost perspective: cost inclusion
1	Lapcharoensap (2020)[33]	US (2008-2011) California Perinatal Quality Care Collaborative	Retrospective cohort	Oxygen requirement >36 weeks PMA	BPD: 2,696 Controls: 5,302	1 year: from birth, including index hospitalization	$442,468	Hospital: Direct hospital costs calculated from charges in discharge records using cost-to-charge ratio
2	Mowitz (2019)[32]	US (2009-2015) Premier Perspective Database	Retrospective cohort	ICD code	BPD: 4,904 Controls: 7,113	Short-term: Index hospitalization	$225,204	Hospital: Direct hospital costs, from billed services
3	Álvarez-Fuente (2017)[29]	Spain (2013) SEN1500 database	Retrospective cohort: Model base case for minimum cost of BPD using weighted costs	Oxygen dependence >28 days after birth (also >36 weeks for severity assessment)	BPD: 2,628	2 years: from birth, including index hospitalization	€45,049-118,760	Health system: Hospital costs and follow-up cost; inpatient costs were estimated using diagnosis related group weights, price lists were used for outpatient visits and immunization
4	Patel (2017)[30]	US (2009-2012) Rush University Medical Center NICU, Chicago/Illinois	Prospective cohort (compare own mothers milk v. formula)	Oxygen requirement >36 weeks PMA	BPD: 77 Controls: 177	Short-term: Index hospitalization	$269,004 (Median)	Hospital: Hospital costs, overhead, and physician payments from a financial data repository with costs for chargeable items
5	Stevens (2017)[31]	US (2008-2011) The California Office of Statewide Health Planning and Development (OSHPD) database	Retrospective cohort: Regression model to identify the cost incurred by infants with specific conditions	ICD code	Total: 2,021,013 BPD: 2189	1 year: from birth, including index hospitalization	$133,140 Cost increase = 22.4%	Hospital: Commercial claims data from the region corrected to national costs using register data from the Healthcare Cost and Utilisation Project data set
6	Johnson (2013)[28]	US (2005-2009) Rush University Medical Center NICU, Chicago/Illinois	Retrospective cohort	Oxygen dependence >28 days after birth (also >36 weeks for severity assessment)	BPD: 230 Controls: 195	Short-term: Index hospitalization	$103,151	Hospital: Hospital costs excluding physician fees from a financial data repository with costs for chargeable items

For peer review only

For peer review only - http://bmjopen.bmj.com/site/about/guidelines.xhtml

7	Landry (2012)[27]	Canada (1983-2008) MED-ECHO and RAMQ databases, Quebec	Retrospective cohort	ICD code	BPD: 773 Controls: 2669	16 - 25 years: from birth, after the index hospitalization	C$13,472	Health system: Hospitalization follow-up cost and prescription drugs; inpatient costs were estimated using a case mix approach, billed fees were used for outpatient visits and drugs
8	Greenough (2011)[26]	UK (2002-2005) Previously presented cohort, hospitals not stated[35]	Retrospective cohort	Oxygen dependence >28 days after birth	BPD: 160	2 years: 5-7 years of age	£523-705 (Median)	Health system: Hospital admission costs**, follow-up cost, and prescription drugs; price lists were used for admissions and drugs, unit costs based on wages and time used by clinicians for outpatient visits
9	Greenough (2006)[25]	UK (1999-2002) Previously presented cohort, hospitals not stated[35]	Retrospective cohort	Oxygen dependence >28 days after birth	BPD: 190	2 years: 2-4 years of age	£950-3,054 (Median)	Health system: Hospital admission costs**, follow-up cost, and prescription drugs; price lists were used for admissions and drugs, unit costs based on wages and time used by clinicians for outpatient visits
10	Akman (2002)[24]	Turkey (1999-2000) Marmara university hospital, Istanbul	Retrospective cohort	Oxygen requirement >36 weeks PMA	BPD: 72	Short-term: Index hospitalization	$14,810 (Marginal cost)	Hospital: Hospital charges collected from the hospital Accounting Department, including physician and nursing services, laboratory and radiology tests and medical supplies
11	Miller (1998)[23]	US (1990) Tulane University Medical Centre, Louisiana	Retrospective cohort	Oxygen dependence >28 days after birth and chest x-ray	BPD:89	4.5 years: from birth, after the index hospitalization	$19,034*	Payer (third party and parents): Direct health costs (registered charges, or costs translated to charges using cost-to-charge ratios), non-health costs (expenses), and indirect cost (lost income) to families
12	McAleese (1993)[22]	US (1981-1989) Dartmouth-Hitchcock Medical Centre, New Hampshire	Retrospective cohort	Oxygen dependence >28 days after birth and chest x-ray	BPD: 59	2 months-2.7 years: from birth, including index hospitalization	Initial hospitalization = $197,668 Home care = $4,262-68,136 (Medians) Indirect costs =	Payer (third party and parents): Hospital charges for hospitalization, charges for follow-up cost and home care equipment. expenses and travel costs, and indirect cost (lost income) to families
13	Monset-Couchard (1984)[21]	France (1981) Port-Royal Intensive Care Unit, Paris	Retrospective cohort	Hospital data	Not stated	Short-term: Index hospitalization	₣336,545	Hospital: Unit costs for hospitalizations, travel charges (including ambulance and air travel)

For peer review only - http://bmjopen.bmj.com/site/about/guidelines.xhtml

For peer review only

Unless otherwise stated, the costs are the mean costs reported in the original paper. Abbreviations: C= Controls; C$= Canadian dollar; ₣= French franc; ICD= international classifications of diseases; PMA= post-menstrual age.

*Mean cost was computed based on the data available in the article

**** Does not include index hospitalization since the year of birth is not included in the time period of each study.**

For peer review only

Risk of bias in included studies

Potential sources of bias included the lack of participant characteristics in some of the included studies. Additionally, some studies lacked information about potential conflicts of interests for the participating researchers (Supplemental table S2). Only one study conducted a sensitivity analysis of their results. None of the included papers clearly stated the research question, but it could be gleaned from the objective of each study. Few studies included comparisons between groups or long-term outcomes, and no study included an evaluation of health outcomes. Thus, some of the CHEC-list items were not suitable for evaluating these studies. Of the 13 included studies, the checklist items judged relevant for each study method were to a large extent also covered in the paper. Only two papers had more than 3 items missing, and the items not covered throughout the papers were mostly related to lack of a well-defined research question and lack of sensitivity analyses. Overall, the assessment suggests a low risk of bias in the included papers.

Total costs for BPD

A majority of studies (Table 2) reported costs during the birth hospitalization and/or the first year of life (8 studies). One study reported follow up costs during the first two years of life including birth hospitalisation. Two other studies reported two-year follow-up costs for age groups 2-4 and 5-7 years, respectively. Two studies included a long-term follow up, with an average follow-up of 4.5 years and 19.3 years, respectively (Figure 2).

Table 2: Costs for infants with BPD

**Reference** *subgroups reported in the paper*	**Total cost (Int$)**	**Cost of index Hospitalization (Int$)**	**Cost of other care (Int$)**	**Cost increase over controls due to BPD, Int$ (%)**
**1. Lapcharoensap (2020)[33]**	477,901	466,216	Rehospitalization: 46,948	246,284 (106%)
≤ 24 weeks	*507,638*			*118,808 (35%)*
25- 26 weeks	*453,633*			*129,609 (40%)*
27-28 weeks	*351,026*			*124,209 (55%)*
> 28 weeks	*313,223*			*151,211 (93%)*
**2. Mowitz (2019)[32]**	see index hospitalization	242,995		58,381 (31%)
≤ 24 weeks	see index hospitalization	*326,962*		*26,975 (9%)*
25- 26 weeks	see index hospitalization	*247,901*		*32,370 (15%)*
27-28 weeks	see index hospitalization	*190,983*		*36,686 (24%)*
> 28 weeks	-	-		-
**3. Álvarez-Fuente (2017)[29]**	74,618 - 196,713		2-year follow-up:	-

For peer review only - http://bmjopen.bmj.com/site/about/guidelines.xhtml

<750g	*196,713*	*182,627*	*14,084*	-
750- 999g	*149,071*	*134,986*	*14,084*	-
1000-1249g	*73,237*	*60,534*	*14,084*	-
> 1249 g	-	-	-	-
**4. Patel (2017)[30]**	see index hospitalization	290,546*<br>Marginal cost:<br>45,287*		164,092*
**5. Stevens (2017)[31]**	148,251	-	-	(22.4%)
**6. Johnson (2013)[28]**	see index hospitalization	122,983		69,969 (132%)
<750g				*51,639*
750- 999g				*41,196*
1000-1249g				*29,317*
> 1249 g				*23,810*
**7. Landry (2012)[27]**	12,973/year		16-25 year follow-up:<br>12,973/year	2,651/year (26%)
**8. Greenough (2011)[26]**<br>(2 years at 5-7 years of age)	Home oxygen:<br>1,135*<br>No oxygen:<br>842*		Home oxygen:<br>1,135*<br>No oxygen:<br>842*	
**9. Greenough (2006)[25]**<br>(2 years at 2-4 years of age)	Home oxygen:<br>5,556*<br>No oxygen:<br>1,728*		Home oxygen:<br>5,556*<br>No oxygen:<br>1,728*	
**10. Akman (2002)[24]**	see index hospitalization	21,392	-	15,116 (241%)
**11. Miller (1998)[23]**	34,692		4.5-year follow-up<br>Inpatient services:<br>25,268<br>Outpatient services:<br>6,625<br>Indirect costs:<br>2800	
**12. McAleese (1993)[22]**	see index hospitalization	387,085	Home care, nurse:<br>133,427*<br>Home care, no nurse:<br>8,346*<br>(including indirect costs)	

For peer review only

**13. Monset-Couchard (1984)[21]**	see index hospitalization	1,094,509

Values are the mean costs, unless otherwise stated. *Median cost; Int$ = The cost after converting to purchasing power parities, then inflating to the 2019 value

Index hospitalization

The mean costs during the index hospitalization ranged from Int$21,392 to Int$1,094,509 (Figure 3). For five studies conducted in the USA, the range of index hospitalisation costs was Int$122,983-466,216. If only including those four US studies limiting inclusion to severe BPD, the range is Int$242,995-466,216.[22 30 32 33]

Hospital readmissions in the first two years of life

One study analysed rehospitalizations among infants with BPD. They showed that rehospitalizations among infants with BPD occurred more than twice at frequently and incurred higher costs than rehospitalizations among their peers without BPD.[33] Rehospitalizations were required in 25.1% of infants with BPD, and the mean cost was estimated to be Int$46,948 during first year of life.[33] One study estimated that the cost associated with BPD for a mean follow-up of the first 2 years of life was Int$14,084.[29]

Follow-up costs and home care

Two studies reported costs during a specific age, thus not including the costs occurring at birth and during the first years of life: Among children with BPD, the median costs during 2 years of follow-up for ages 2-4 were estimated at Int$5,556 for those that required home oxygen after the initial discharge, compared to Int$1,728 for those that did not require home oxygen after the initial discharge from the hospital.[25] Furthermore, the median costs during 2 years of follow-up for ages 5-7 were Int$1,135 for those that required home oxygen, compared to Int$842 for those that did not require home oxygen after the initial discharge.[26] Children initially requiring oxygen during a longer period also required more resources at older ages.

Other studies reported costs from birth and onwards, but excluding the index hospitalisation: One study reported that the median follow-up cost of healthcare for children with BPD 4.5 years after initial hospitalisation was Int$25,268 for inpatient care and Int$6,625 for outpatient services.[23] One study analysed the cost of healthcare for children with BPD during a follow up for ages 16-25 years; they reported that the annual cost was Int$12,973 per person.[27]

Moreover, one study reported that the median cost of home healthcare (including indirect costs for lost wages) was Int$133,427 with a nurse and Int$8,346 without a nurse.[22] Thus, costs for home healthcare were largely dependent on the possibilities to employ a nurse, based on private insurance payments and other funds.

Indirect costs

One study reported indirect cost of Int$2,800 during 4.5 years after the initial hospitalisation.[23] One study reported indirect costs as part of the home care costs, and for the 30 families reporting loss of wages this corresponded to a range of Int$516-44,105 per family (with 9-577 days of lost income) in 2019 value.[22]

Increase in costs due to BPD

In four of the included studies, BPD incurred, on average, 24 – 71% extra expenses in healthcare. One study from Canada reported that the mean follow-up cost increased by Int$2,651/year, which was a 24% increase over the cost for preterm peers without BPD.[27]

For peer review only - http://bmjopen.bmj.com/site/about/guidelines.xhtml

In four studies that reported costs according to GA categories, the cost ranges were: Int$196,713 – 507,638 for those born at or before GA 24 weeks/<750 g; Int$149,071 – 453,633 for those born at GA 25-26 weeks/750-999 g; and Int$73,237 – 351,027 for those born at GA 27-28 weeks/1000-1249 g. One study reported a cost of Int$313,223 for those born at GA 29 weeks or later/>1249 g (Figure 3). Higher cost increases were reported for infants with BPD born at higher GAs compared to those born at lower GAs. For example, studies reported that the mean costs for infants with BPD increased by 9 – 35% for those born at or before GA 24 weeks; by 15 – 35% for those born at GA 25-26 weeks; and by 19 – 35% for those born at GA 27-28 weeks. One study reported a 93% cost increase for infants born at GA 29 weeks or later. The mean total cost increase for infants with BPD, stratified by GA, are shown in Figure 4.

DISCUSSION

The included studies reported high costs for preterm infants with BPD during the initial hospitalization after birth (range: Int$21,392–1,094,509 per child, equivalent to €19,103–977,397, in 2019). However, looking at only four studies conducted in one country and with similar inclusion criteria, the range was Int$242,995-466,216. Moreover, the studies reported a high use of resources associated with BPD during the following years, up to school age. The highest mean costs were associated with infants born at extremely low GAs.

Several prior reviews and modelling studies in this field [15 36] have reported costs, but they based study inclusions on expert opinions and targeted searches. Therefore, the present study provided an overview of all the evidence available on costs for BPD, with an expected low risk of bias in included studies based on the quality assessment tool. The main limitation of this study was, however, the lack of searches for identifying grey literature and not performing analysis of publication bias. However, we did perform an initial search based on references identified in, e.g., modelling studies and reviews, when we designed the search strategy, to ensure that we had identified all the relevant publications with our search strategy. We found several studies that addressed costs of interventions, such as immunization or infection treatment for infants with BPD, but in those cases, the costs were not included for BPD *per se*. Our search was tailored specifically to identify articles on BPD; thus, we excluded articles that used an alternate term, like chronic lung disease (CLD). We identified very few articles that were relevant, considering the great need of quality long-term healthcare for preterm infants with BPD. Moreover, we initially intended to conduct a meta-analysis of results, but we found that, in the separate papers, the costs reported differed too extensively in content and time-horizons to support that type of analysis. For the same reason, we could not conduct a formalized synthesis of the results. Moreover, the recently published guidelines for a data synthesis without a meta-analysis [37] were not suitable for this study; instead they were more suited to the synthesis of effect estimates for intervention studies. Consequently, the analysis herein was descriptive.

Another factor making comparisons between results in included studies difficult was that, in the BPD literature, the operational definition of BPD has varied since the condition was first discovered.[9] Thus, the extent of variability we encountered in the included papers (detailed in the Results section) might have affected the selection of infants, which might have influenced the healthcare costs. It is also important to note that the costs of BPD reported in the selected studies was based on the costs incurred by oxygen administration. However, the full economic impact of BPD should also include the costs associated with preventing BPD development in infants at risk. This same limitation applies to all retrospective studies that based the definition of BPD on ICD codes and clinical assessments, because infants that successfully avoided BPD development would not be classified as BPD, either with the codes or in clinical assessments.

Regarding the cost estimations, the majority of the included articles focused on the initial hospitalization. However, after the initial hospitalization, a considerable number of infants with BPD require rehospitalization during the first years of life, due to respiratory illnesses.[33] Infants born prematurely often display symptoms and signs of obstructive airway disease that persist for prolonged time periods; thus, it is important to implement standardized, regular follow-up schedules.[38]

Our findings showed how the cost estimates for BPD varied across different countries and during different time periods. Table 2 shows a large cost discrepancy among different countries, e.g., Turkey and the USA, which might be explained by several factors. First, BPD treatment programs might differ with different healthcare providers, during

For peer review only - http://bmjopen.bmj.com/site/about/guidelines.xhtml

different years, and in different countries.[39] For example, articles that investigated the costs of BPD in the USA reported much higher costs than those reported in other countries; this discrepancy might partly be explained by higher cost of healthcare, in general, in the USA.[40] Among studies conducted in the USA, the study by Johnson and colleagues[28] stand out in comparison with lower costs for the initial hospitalisation, possibly due to the exclusion of physician fees or from the BPD definition being inclusive also of milder cases. Second, the included studies spanned a long time period, where healthcare will have changed dramatically due to technological development. It is clear from the data provided that care of infants with BPD has been resource intense throughout the period, and the inflated estimates indicate the current value of the resources used at that time, but it is difficult to compare costs between a study from the early 1980s to those conducted in 2010s. The patient group will look very different, with more infants born very early surviving to develop BPD.In addition to the potential effects of different BPD definitions, infants with BPD frequently have other comorbidities, such as brain injury and retinopathy of prematurity (ROP), which can lead to functional impairments that clearly impact the cost of healthcare.[41] The cost of BPD without a comorbidity can be difficult to differentiate from the total cost including comorbidities. For example, the presence of comorbidities, like pulmonary hypertension (PH), in infants with BPD can significantly increase the healthcare costs, due to longer hospitalizations and a higher likelihood of requiring home respiratory support.[42] Third, the quality of healthcare can differ between different healthcare providers, which can also result in cost differences. Fourth, some of the included articles omitted data for some costs; e.g., the cost of physicians.[28] Finally, as previously discussed, an SGA condition can exacerbate the severity of BPD; this condition was not taken into account in the cost calculations, because the included articles did not report the combined effects of GA and BW.

Two studies compared healthcare costs between preterm infants with BPD and their preterm peers without BPD. Those data enabled a calculation of the cost increase due to BPD. The results clearly indicated that the cost differences were higher among infants born at lower GAs. This finding was consistent with cost estimates for other diagnoses in the same patient groups, such as infants born to mothers with preeclampsia.[31] Among our included studies, the study by Johnson et al [28] estimated marginal costs in infants with BPD compared to infants of the same weight without any of four major comorbidities (sepsis, brain injury, necrotizing enterocolitis [NEC], or BPD). They found that the cost increase was largest among the smallest infants (<750 g). Lapcharoensap et al [33] compared infants with BPD to all other infants of the same age (no limitation on comorbidities). They reported the opposite relationship, where larger cost differences were observed among those born at higher GAs. This relationship might be expected, because, in the reference groups, infants born at higher GAs can be expected to have fewer of the major comorbidities than infants born at lower GAs; thus, the high GA group would show a larger cost difference between infants with and without BPD. This effect was also reported by Mowitz et al.,[32] although the detailed cost data were not available. They reported that, among all children (regardless of comorbidity), the cost increase due to BPD was largest for infants born at a higher GA; however, when infants with BPD were compared to those without BPD, ROP, or intraventricular haemorrhage (IVH), the cost increase was similar independent of GA. In contrast, the study by Johnson et al. showed a clear trend towards a higher cost difference due to BPD among the smallest infants. The differences between the two latter studies might be explained by the fact that the comorbidities excluded in the study by Johnson et al [28] (the comparison group had no sepsis, brain injury, NEC, or BPD) were different from those excluded in the study by Mowitz et al [32] (the comparison group had no ROP, IVH, or BPD).

It is clear from the included studies that infants and children with BPD often required long-term follow-ups. This requirement affects the life of these infants in various direct and indirect ways, which have not been properly investigated. A recent modelling study,[36] based on a targeted literature search, estimated the life-time burden of BPD for infants born ≤28 weeks GA. That study explored how BPD severity and major complications affected other aspects of life, like clinic visits for wheezing episodes, asthma exacerbations, psychiatric illnesses, et cetera. As expected, the model found that BPD had a large impact on healthcare use, quality-of-life, and survival, but it also showed that the degree of impact was largely dependent on BPD severity. Accordingly, improvements in BPD therapy might substantially reduce the impact of BPD.[36] However, as those authors acknowledged, the model could only use the available data, and thus, it was limited by, e.g., data on each complication separately (not accounting for concomitant diagnoses in one patient potentially increasing costs more than expected from single diagnosis studies). That study also reported that the healthcare cost of BPD for the first year of life was C$197,100, which was consistent with the estimates of more recent studies included in this review.

For peer review only - http://bmjopen.bmj.com/site/about/guidelines.xhtml

The findings from both our systematic review and the previous modelling study [36] suggested that there is a need for more research on the long-term economic impact of BPD. Preferably, those studies should also include costs that occur outside the health system, such as costs due to changes in parent productivity and costs for schooling and day care. In countries like the Nordics, where much information about resource use is available in national and/or regional registers collected for administrative purposes and available for research, many of the relevant resources can in be studied in retrospective register studies.[43 44] However, some types of information is less likely to be included in registers, such as informal care provided by parents and other relatives. Thus, studies using prospective and retrospective data collection methods should both add important knowledge on the long-term costs associated with BPD. Moreover, studies should consider the costs related to the combined impact of complications. It has been argued that studies with a societal perspective can better identify potential inequities and inefficient use of society's resources.[45] One can speculate that one reason for the paucity of cost studies on BPD is that new drug developments have slowed after the success of surfactant therapy, which was introduced in the early 1990s.[46] Although several advances in the care of preterm infants and BPD have improved survival and symptom reduction,[47] these care applications and treatments have not, to the same extent, been subjected to economic and cost assessments.[48] To minimize the growing burden of BPD, it is vital to prevent prematurity, in the first place, and in the second place, to develop new treatments for prematurity-related diseases.[49] It is possible that the ongoing research on administration of stem cells, anti-inflammatory agents and IGF-I/IGFBP-3 among extremely pre-term infants[9] will spark a renewed interest in how resources are best used for this patient group.

This systematic review confirmed previous reports that BPD in preterm infants was associated with high costs and required long-term follow-ups. Our major finding was that the highest costs occurred among infants born at lower GAs, and that this relationship was difficult to detect, unless an appropriate control group was used. Moreover, we identified a shortage of studies that estimated, in particular, costs that occurred outside hospitals and after the first year of life. Further research on BPD is required to gain a proper understanding of the long-term consequences regarding healthcare costs and the social aspects of BPD.

For peer review only

For peer review only - http://bmjopen.bmj.com/site/about/guidelines.xhtml

Funding statement

JH was financed by the University of Gothenburg Centre for Person-Centred Care (GPCC) teaching assistant program, and the Mary von Sydow Foundation. CL was financed by the University of Gothenburg Centre for Person-Centred Care (GPCC). DL was financed by the Swedish Research Council (#2018-00770). AH was supported by the Swedish Research Council (#2016-01131), the Gothenburg Medical Society, De Blindas Vänner, and Government grants, under the ALF agreements (ALFGBG-717971 and ALFGBG-812951), and The Wallenberg Clinical Scholars. HG was financed by the Swedish Research Council (#2016-01131) and the Swedish Research Council (#2018-00770). The funders played no role in the design of the study or writing the protocol.

Competing interests statement

JH report no competing interests. CL hold stocks in Premalux AB. DL and AH hold stock/stock options in Premalux AB, and have received consulting fees from Takeda Inc. HG is employed part-time by Statfinn and EPID Research (part of IQVIA), which is a contract research organisation that performs commissioned pharmacoepidemiological studies and thus its employees have been and currently are working in collaboration with several pharmaceutical companies.

Contributions

All authors contributed to the design of the study. JH, CL, and HG designed the database search and data extraction methods. JH and HG undertook the literature search, assessed studies for eligibility and extracted data. In case of disagreement assessments were made in discussion with CL. DL and AH contributed clinical expertise on preterm infants and morbidity. JH, CL and HG discussed the data and interpreted the results. JH and HG drafted the manuscript. All authors critically reviewed and approved the final manuscript.

Acknowledgements

This systematic review was part of the Less is More project (Swedish Research Council grant number #2018-00770), a longitudinal study of clinical and economic outcomes in children born at very low gestational age. This study thus contributes with an understanding of the current knowledge and important gaps to the Less is More study design for long-term follow-up and economic evaluation.

Data Availability Statement

All data relevant to the study are included in the article. Original data is available in the reviewed publications which are all cited. Additional data from the data extraction performed are available upon reasonable request from the corresponding author.

For peer review only

References

1. Hwang JS, Rehan VK. Recent Advances in Bronchopulmonary Dysplasia: Pathophysiology, Prevention, and Treatment. *Lung* 2018;**196**(2):129-38.
2. Northway WH, Jr., Rosan RC, Porter DY. Pulmonary disease following respirator therapy of hyaline-membrane disease. Bronchopulmonary dysplasia. *N Engl J Med* 1967;**276**(7):357-68.
3. Yeh TF, Lin HC, Chang CH, et al. Early intratracheal instillation of budesonide using surfactant as a vehicle to prevent chronic lung disease in preterm infants: a pilot study. *Pediatrics* 2008;**121**(5):e1310-8.
4. Tyson JE, Wright LL, Oh W, et al. Vitamin A supplementation for extremely-low-birth-weight infants. National Institute of Child Health and Human Development Neonatal Research Network. *N Engl J Med* 1999;**340**(25):1962-8.
5. Schmidt B, Roberts RS, Davis P, et al. Caffeine therapy for apnea of prematurity. *N Engl J Med* 2006;**354**(20):2112-21.
6. Bancalari E, del Moral T. Bronchopulmonary dysplasia and surfactant. *Biol Neonate* 2001;**80 Suppl 1**:7-13.
7. Rojas MA, Gonzalez A, Bancalari E, et al. Changing trends in the epidemiology and pathogenesis of neonatal chronic lung disease. *J Pediatr* 1995;**126**(4):605-10.
8. Coalson JJ. Pathology of new bronchopulmonary dysplasia. *Semin Neonatol* 2003;**8**(1):73-81.
9. Thebaud B, Goss KN, Laughon M, et al. Bronchopulmonary dysplasia. *Nat Rev Dis Primers* 2019;**5**(1):78.
10. Norman M, Hallberg B, Abrahamsson T, et al. Association Between Year of Birth and 1-Year Survival Among Extremely Preterm Infants in Sweden During 2004-2007 and 2014-2016. *JAMA* 2019;**321**(12):1188-99.
11. Qiu X, Lodha A, Shah PS, et al. Neonatal outcomes of small for gestational age preterm infants in Canada. *Am J Perinatol* 2012;**29**(2):87-94.
12. Ehrenkranz RA, Walsh MC, Vohr BR, et al. Validation of the National Institutes of Health consensus definition of bronchopulmonary dysplasia. *Pediatrics* 2005;**116**(6):1353-60.
13. Blencowe H, Cousens S, Oestergaard MZ, et al. National, regional, and worldwide estimates of preterm birth rates in the year 2010 with time trends since 1990 for selected countries: a systematic analysis and implications. *Lancet* 2012;**379**(9832):2162-72.
14. Bhandari A, McGrath-Morrow S. Long-term pulmonary outcomes of patients with bronchopulmonary dysplasia. *Semin Perinatol* 2013;**37**(2):132-7.
15. Lapcharoensap W, Lee H, Nyberg A, et al. Health Care and Societal Costs of Bronchopulmonary Dysplasia. *NeoReviews* 2018;**19**:e211-e23.
16. Evers S, Goossens M, de Vet H, et al. Criteria list for assessment of methodological quality of economic evaluations: Consensus on Health Economic Criteria. *Int J Technol Assess Health Care* 2005;**21**(2):240-5.
17. von der Warth R, Dams J, Grochtdreis T, et al. Economic evaluations and cost analyses in posttraumatic stress disorder: a systematic review. *Eur J Psychotraumatol* 2020;**11**(1):1753940.
18. Dangouloff T, Botty C, Beaudart C, et al. Systematic literature review of the economic burden of spinal muscular atrophy and economic evaluations of treatments. *Orphanet J Rare Dis* 2021;**16**(1):47.
19. OECD. Purchasing power parities (PPP) (indicator). Secondary Purchasing power parities (PPP) (indicator) 2020. https://data.oecd.org/conversion/purchasing-power-parities-ppp.htm.
20. OECD. Inflation (CPI) (Indicator). Secondary Inflation (CPI) (Indicator) 2020. https://data.oecd.org/price/inflation-cpi.htm.
21. Monset-Couchard M, Jaspar ML, de Bethmann O, et al. [Costs of the initial care of children with birth weight less than or equal to 1500 g in 1981]. *Arch Fr Pediatr* 1984;**41**(8):579-85.
22. McAleese KA, Knapp MA, Rhodes TT. Financial and emotional cost of bronchopulmonary dysplasia. *Clin Pediatr (Phila)* 1993;**32**(7):393-400.
23. Miller VL, Rice JC, DeVoe M, et al. An analysis of program and family costs of case managed care for technology-dependent infants with bronchopulmonary dysplasia. *J Pediatr Nurs* 1998;**13**(4):244-51.
24. Akman Y, Cebecý D, Özek E, et al. Effects of perinatal factors on the duration and cost of hospitalisation for preterm infants in a neonatal intensive care unit in Istanbul. *Turkish Journal of Medical Sciences* 2002;**32**:159-63.
25. Greenough A, Alexander J, Burgess S, et al. Preschool healthcare utilisation related to home oxygen status. *Arch Dis Child Fetal Neonatal Ed* 2006;**91**(5):F337-41.
26. Greenough A, Alexander J, Boorman J, et al. Respiratory morbidity, healthcare utilisation and cost of care at school age related to home oxygen status. *Eur J Pediatr* 2011;**170**(8):969-75.

For peer review only - http://bmjopen.bmj.com/site/about/guidelines.xhtml

27. Landry JS, Croitoru D, Jin Y, et al. Health care utilization by preterm infants with respiratory complications in Quebec. *Can Respir J* 2012;**19**(4):255-60.
28. Johnson TJ, Patel AL, Jegier BJ, et al. Cost of morbidities in very low birth weight infants. *J Pediatr* 2013;**162**(2):243-49 e1.
29. Alvarez-Fuente M, Arruza L, Muro M, et al. The economic impact of prematurity and bronchopulmonary dysplasia. *Eur J Pediatr* 2017;**176**(12):1587-93.
30. Patel AL, Johnson TJ, Robin B, et al. Influence of own mother's milk on bronchopulmonary dysplasia and costs. *Arch Dis Child Fetal Neonatal Ed* 2017;**102**(3):F256-F61.
31. Stevens W, Shih T, Incerti D, et al. Short-term costs of preeclampsia to the United States health care system. *Am J Obstet Gynecol* 2017;**217**(3):237-48 e16.
32. Mowitz ME, Ayyagari R, Gao W, et al. Health Care Burden of Bronchopulmonary Dysplasia Among Extremely Preterm Infants. *Front Pediatr* 2019;**7**:510.
33. Lapcharoensap W, Bennett MV, Xu X, et al. Hospitalization costs associated with bronchopulmonary dysplasia in the first year of life. *J Perinatol* 2020;**40**(1):130-37.
34. Jobe AH, Bancalari E. Bronchopulmonary dysplasia. *Am J Respir Crit Care Med* 2001;**163**(7):1723-9.
35. Greenough A, Cox S, Alexander J, et al. Health care utilisation of infants with chronic lung disease, related to hospitalisation for RSV infection. *Arch Dis Child* 2001;**85**(6):463-8.
36. van Katwyk S, Augustine S, Thebaud B, et al. Lifetime patient outcomes and healthcare utilization for Bronchopulmonary dysplasia (BPD) and extreme preterm infants: a microsimulation study. *BMC Pediatr* 2020;**20**(1):136.
37. Campbell M, McKenzie JE, Sowden A, et al. Synthesis without meta-analysis (SWiM) in systematic reviews: reporting guideline. *BMJ* 2020;**368**:l6890.
38. Landry JS, Chan T, Lands L, et al. Long-term impact of bronchopulmonary dysplasia on pulmonary function. *Can Respir J* 2011;**18**(5):265-70.
39. Williams I, Brown H, Healy P. Contextual Factors Influencing Cost and Quality Decisions in Health and Care: A Structured Evidence Review and Narrative Synthesis. *Int J Health Policy Manag* 2018;**7**(8):683-95.
40. Ridic G, Gleason S, Ridic O. Comparisons of health care systems in the United States, Germany and Canada. *Mater Sociomed* 2012;**24**(2):112-20.
41. Schmidt B, Roberts RS, Davis PG, et al. Prediction of Late Death or Disability at Age 5 Years Using a Count of 3 Neonatal Morbidities in Very Low Birth Weight Infants. *J Pediatr* 2015;**167**(5):982-6 e2.
42. Stuart BD, Sekar P, Coulson JD, et al. Health-care utilization and respiratory morbidities in preterm infants with pulmonary hypertension. *J Perinatol* 2013;**33**(7):543-7.
43. Riley GF. Administrative and claims records as sources of health care cost data. *Med Care* 2009;**47**(7 Suppl 1):S51-5.
44. Alriksson-Schmidt AI, Jeglinsky-Kankainen IFD, Jahnsen RB, et al. Flaunting our assets. Making the most of the Nordic registry goldmine: Cerebral palsy as an example. *Scand J Public Health* 2020;**48**(1):113-18.
45. Johannesson M, Jonsson, B., Jonsson, L., Kobelt, G., & Zethraeus, N. Why Should Economic Evaluations of Medical Innovations Have a Societal Perspective? *Office of health economics (OHE)* 2009(51, October 2009).
46. Curstedt T, Halliday HL, Speer CP. A unique story in neonatal research: the development of a porcine surfactant. *Neonatology* 2015;**107**(4):321-9.
47. Principi N, Di Pietro GM, Esposito S. Bronchopulmonary dysplasia: clinical aspects and preventive and therapeutic strategies. *J Transl Med* 2018;**16**(1):36.
48. Zupancic JAF. A Systematic Review of Costs Associated with Preterm Birth. In: and CoUPB, Outcomes AH, eds. Preterm Birth: Causes, Consequences, and

Prevention National Academies Press (US), 2007:688 - 724.
49. Davidson LM, Berkelhamer SK. Bronchopulmonary Dysplasia: Chronic Lung Disease of Infancy and Long-Term Pulmonary Outcomes. *J Clin Med* 2017;**6**(1).

For peer review only - http://bmjopen.bmj.com/site/about/guidelines.xhtml

FIGURE LEGENDS

Figure 1: Flow diagram shows the study selection process, in accordance with the PRISMA guidelines. Abbreviations: BPD = Bronchopulmonary Dysplasia

Figure 2: Overview of the costs of BPD and the study period included in each article. The numbers on the Y-axis refer to the numbers assigned to each study (Table 1).

Figure 3: Costs of the index hospitalizations for preterm infants with BPD in studies conducted between 1981 and 2015. All costs were converted to Int$, based on purchasing power parities, then the values were inflated to 2019 values. Abbreviations: BPD = Bronchopulmonary Dysplasia; control infants = preterm infants without BPD; mInt$ = millions of Int$.

Figure 4: Mean cost increase for infants with BPD, stratified by gestational age (GA) at birth. The numbers on the x-axis refer to the numbers assigned to studies in Table 1. Abbreviations: BPD = Bronchopulmonary dysplasia; Int$ = The cost after converting to purchasing power parities, then inflating to the 2019 value; mInt$ = millions of Int$, w = weeks

For peer review only

For peer review only - http://bmjopen.bmj.com/site/about/guidelines.xhtml

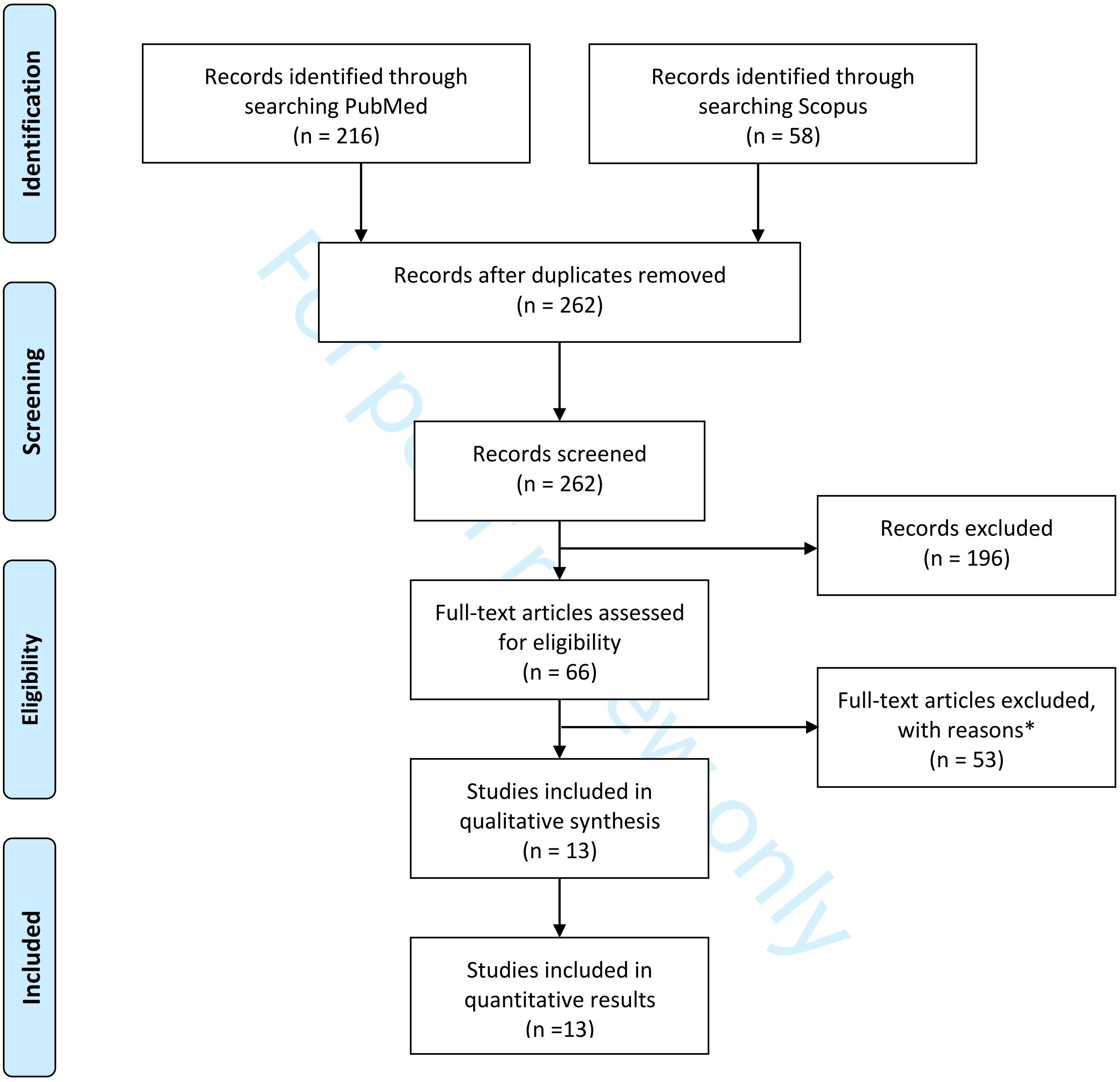

* Reasons for exclusion after full text assessment:
- Cost data not specific to BPD (n = 26)
- Only billed charges, no detailed cost estimates (n = 2)
- No cost analysis with original data (n = 25)

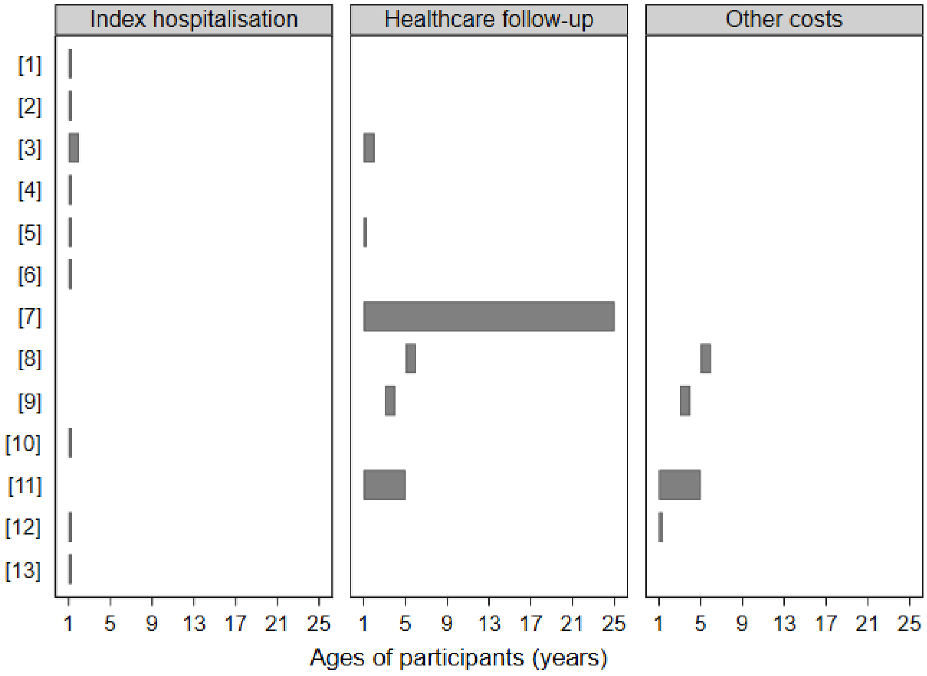

Figure 2Overview of the costs of BPD and the study period included in each article. The numbers on the Y-axis refer to the numbers assigned to each study (Table 1).

244x177mm (72 x 72 DPI)

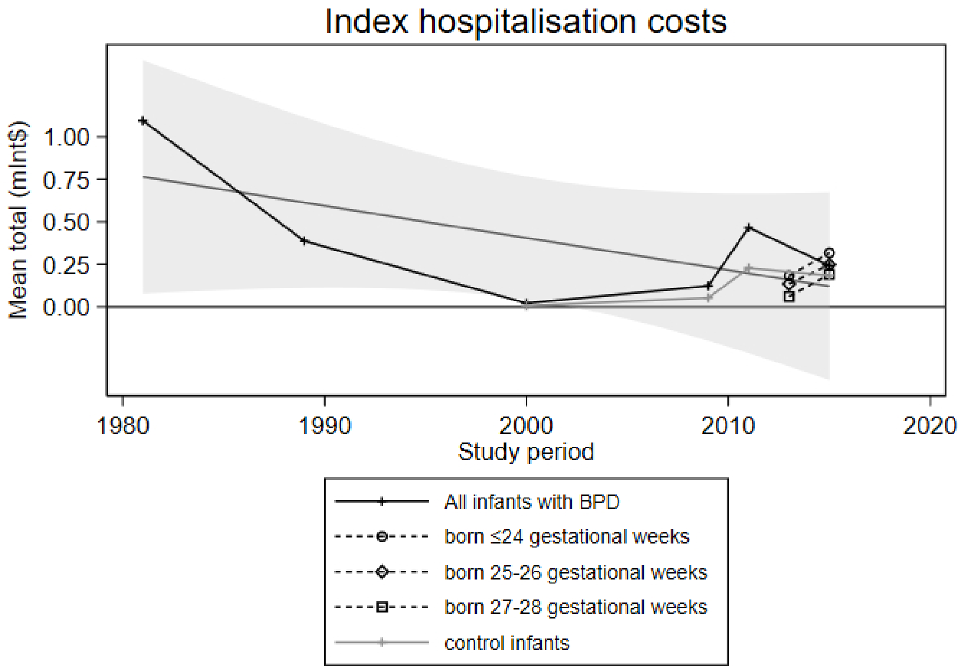

Figure 3Costs of the index hospitalizations for preterm infants with BPD in studies conducted between 1981 and 2015. All costs were converted to Int$, based on purchasing power parities, then the values were inflated to 2019 values. Abbreviations: BPD = Bronchopulmonary Dysplasia; control infants = preterm infants without BPD; mInt$ = millions of Int$.

244x177mm (72 x 72 DPI)

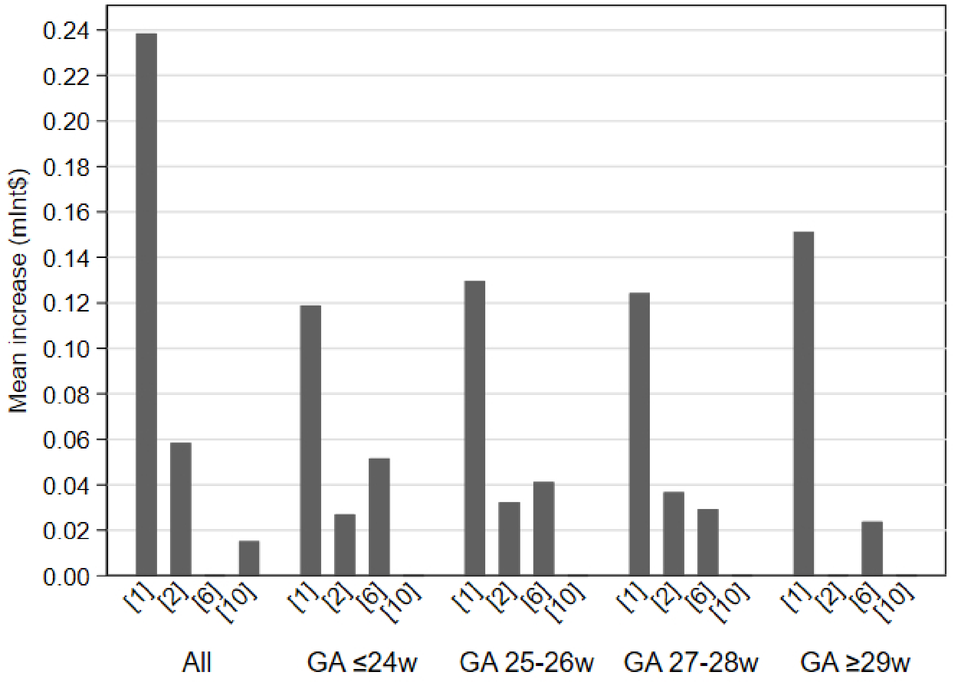

Figure 4
Mean cost increase for infants with BPD, stratified by gestational age (GA) at birth. The numbers on the x-axis refer to the numbers assigned to studies in Table 1. Abbreviations: BPD = Bronchopulmonary dysplasia; Int$ = The cost after converting to purchasing power parities, then inflating to the 2019 value; mInt$ = millions of Int$, w = weeks

244x177mm (72 x 72 DPI)

SUPPLEMENTAL MATERIAL

SYSTEMATIC REVIEW OF THE HEALTHCARE COST OF BRONCHOPULMONARY DYSPLASIA

AUTHORS

Jhangir Humayun[1,2], gushumjh@student.gu.se

Chatarina Löfqvist[1,2,3] chatarina.lofqvist@gu.se

David Ley[4], david.ley@med.lu.se

Ann Hellström[3], ann.hellstrom@medfak.gu.se

Hanna Gyllensten[1,2] hanna.gyllensten@gu.se

Author affiliations

[1] Institute of Health and Care Sciences, Sahlgrenska Academy, University of Gothenburg, Box 457, SE-405 30 Gothenburg, Sweden.

[2] Centre for Person-Centred Care (GPCC), University of Gothenburg, Box 457, SE-405 30 Gothenburg, Sweden.

[3] Department of Clinical Neuroscience, Institute of Neuroscience and Physiology, Sahlgrenska Academy, University of Gothenburg, Gothenburg, Sweden;

[4] Department of Pediatrics, Institute of Clinical Sciences Lund, Lund University and Skåne University Hospital, Lund, Sweden

Table S1: Search terms

Database	Search string
PubMed	((("Bronchopulmonary Dysplasia"[Title/Abstract]) OR "Bronchopulmonary Dysplasia"[Mesh])) AND (("Economics"[Mesh]) OR ((economic*[Title/Abstract] OR cost[Title/Abstract] OR costs[Title/Abstract] OR costly[Title/Abstract] OR costing[Title/Abstract] OR price[Title/Abstract] OR prices[Title/Abstract] OR pricing[Title/Abstract] OR pharmacoeconomic*[Title/Abstract])))
Scopus	(TITLE-ABS-KEY ("Bronchopulmonary Dysplasia")) AND (TITLE-ABS-KEY (economic* OR cost OR costs OR costly OR costing OR price OR prices OR pricing OR pharmacoeconomic*))

For peer review only

Table S2: Checklist for the quality appraisal of included papers (from [15])

Checklist items	21	30	29	26	28	27	25	24	23	22	20	19	18
Is the study population clearly described?	-	+	+	-	+	+	+	+	+	+	-	+	-
Are competing alternatives clearly described?	NA	NA	NA	NA	NA	+	NA	NA	+	+	NA	NA	NA
Is a well-defined research question posed in answerable form?	-	-	-	-	-	-	-	-	-	-	-	-	-
Is the economic study design appropriate to the stated objective?	+	+	+	+	+	+	+	+	+	+	+	+	+
Is the chosen time horizon appropriate in order to include relevant costs and consequences?	NA	NA	NA	NA	NA	NA	NA	NA	NA	NA	NA	NA	NA
Is the actual perspective chosen appropriate?	+	+	+	+	+	+	+	+	+	+	+	+	+
Are all important and relevant costs for each alternative identified?	+	+	+	+	+	+	-	+	+	+	+	+	+
Are all costs measured appropriately in physical units?	+	+	+	+	+	+	+	-	+	+	+	+	+
Are costs valued appropriately?	+	+	+	+	+	+	+	+	+	+	+	+	+
Are all important and relevant outcomes for each alternative identified?	NA	NA	NA	NA	NA	NA	NA	NA	NA	NA	NA	NA	NA
Are all outcomes measured appropriately?	NA	NA	NA	A	NA	NA	NA	NA	NA	NA	NA	NA	NA
Are outcomes valued appropriately?	NA	NA	NA	NA	NA	NA	NA	NA	NA	NA	NA	NA	NA
Is an incremental analysis of costs and outcomes of alternatives performed?	NA	NA	NA	NA	NA	NA	NA	NA	NA	NA	NA	NA	NA
Are all future costs and outcomes discounted appropriately?	NA	NA	NA	NA	+	NA	+	+	+	+	-	+	+
Are all important variables, whose values are uncertain, appropriately subjected to sensitivity analysis?	NA	-	-	-	+	-	-	-	-	-	-	-	-
Do the conclusions follow from the data reported?	-	+	+	+	+	+	+	+	+	+	+	+	+
Does the study discuss the generalizability of the results to other settings and patient/client groups?	-	+	+	+	+	+	+	+	+	+	+	+	+
Does the article indicate that there is no potential conflict of interest of study researcher(s) and funder(s)?	-	+	+	+	+	+	+	+	+	+	-	-	-
Are ethical and distributional issues discussed appropriately?	+	+	+	+	+	+	+	+	+	+	+	+	+
Number of checklist items fulfilled by each individual study	6	10	10	9	12	11	10	10	12	12	8	10	9

+ indicates a fulfilled checklist item, - indicates that the item was either not stated or not fulfilled, NA indicates that the checklist item was not relevant for that study

* Number of studies that fulfilled each checklist item (not considering the applicability of the checklist item to the individual studies

For peer review only

Section/topic	#	PRISMA checklist item	Reported on page #
**TITLE**			
Title	1	Identify the report as a systematic review, meta-analysis, or both.	1
**ABSTRACT**			
Structured summary	2	Provide a structured summary including, as applicable: background; objectives; data sources; study eligibility criteria, participants, and interventions; study appraisal and synthesis methods; results; limitations; conclusions and implications of key findings; systematic review registration number.	2
**INTRODUCTION**			
Rationale	3	Describe the rationale for the review in the context of what is already known.	4
Objectives	4	Provide an explicit statement of questions being addressed with reference to participants, interventions, comparisons, outcomes, and study design (PICOS).	5
**METHODS**			
Protocol and registration	5	Indicate if a review protocol exists, if and where it can be accessed (e.g., Web address), and, if available, provide registration information including registration number.	6
Eligibility criteria	6	Specify study characteristics (e.g., PICOS, length of follow-up) and report characteristics (e.g., years considered, language, publication status) used as criteria for eligibility, giving rationale.	6
Information sources	7	Describe all information sources (e.g., databases with dates of coverage, contact with study authors to identify additional studies) in the search and date last searched.	6
Search	8	Present full electronic search strategy for at least one database, including any limits used, such that it could be repeated.	Table S1
Study selection	9	State the process for selecting studies (i.e., screening, eligibility, included in systematic review, and, if applicable, included in the meta-analysis).	6
Data collection process	10	Describe method of data extraction from reports (e.g., piloted forms, independently, in duplicate) and any processes for obtaining and confirming data from investigators.	6-7
Data items	11	List and define all variables for which data were sought (e.g., PICOS, funding sources) and any assumptions and simplifications made.	7
Risk of bias in individual studies	12	Describe methods used for assessing risk of bias of individual studies (including specification of whether this was done at the study or outcome level), and how this information is to be used in any data synthesis.	7
Summary measures	13	State the principal summary measures (e.g., risk ratio, difference in means).	7
Synthesis of results	14	Describe the methods of handling data and combining results of studies, if done, including measures of consistency (e.g., I^2) for each meta-analysis.	7

For peer review only

Section/topic	#	PRISMA checklist item	Reported on page #
Risk of bias across studies	15	Specify any assessment of risk of bias that may affect the cumulative evidence (e.g., publication bias, selective reporting within studies).	NA
Additional analyses	16	Describe methods of additional analyses (e.g., sensitivity or subgroup analyses, meta-regression), if done, indicating which were pre-specified.	7
**RESULTS**			
Study selection	17	Give numbers of studies screened, assessed for eligibility, and included in the review, with reasons for exclusions at each stage, ideally with a flow diagram.	7<br>Figure 1
Study characteristics	18	For each study, present characteristics for which data were extracted (e.g., study size, PICOS, follow-up period) and provide the citations.	Table 1
Risk of bias within studies	19	Present data on risk of bias of each study and, if available, any outcome level assessment (see item 12).	10<br>Table S2
Results of individual studies	20	For all outcomes considered (benefits or harms), present, for each study: (a) simple summary data for each intervention group (b) effect estimates and confidence intervals, ideally with a forest plot.	Table 2
Synthesis of results	21	Present results of each meta-analysis done, including confidence intervals and measures of consistency.	NA
Risk of bias across studies	22	Present results of any assessment of risk of bias across studies (see Item 15).	10
Additional analysis	23	Give results of additional analyses, if done (e.g., sensitivity or subgroup analyses, meta-regression [see Item 16]).	12
**DISCUSSION**			
Summary of evidence	24	Summarize the main findings including the strength of evidence for each main outcome; consider their relevance to key groups (e.g., healthcare providers, users, and policy makers).	12
Limitations	25	Discuss limitations at study and outcome level (e.g., risk of bias), and at review-level (e.g., incomplete retrieval of identified research, reporting bias).	13
Conclusions	26	Provide a general interpretation of the results in the context of other evidence, and implications for future research.	13
**FUNDING**			
Funding	27	Describe sources of funding for the systematic review and other support (e.g., supply of data); role of funders for the systematic review.	14

Abbreviations: NA = Not applicable.

For peer review only

BMJ Open

SYSTEMATIC REVIEW OF THE HEALTHCARE COST OF BRONCHOPULMONARY DYSPLASIA

Journal:	*BMJ Open*
Manuscript ID	bmjopen-2020-045729.R2
Article Type:	Original research
Date Submitted by the Author:	20-Jul-2021
Complete List of Authors:	Humayun, Jhangir; University of Gothenburg, Institute of Health and Care Sciences; University of Gothenburg Centre for Person-Centred Care<br>Löfqvist, Chatarina; University of Gothenburg Sahlgrenska Academy, Institute of Health and Care Sciences; University of Gothenburg Institute of Neuroscience and Physiology, Department of Clinical Neuroscience<br>Ley, David; Lund University, Department of Pediatrics, Institute of Clinical Sciences and Skåne University Hospital<br>Hellström, Ann; University of Gothenburg Institute of Neuroscience and Physiology, Department of Clinical Neuroscience<br>Gyllensten, Hanna; University of Gothenburg, Institute of Health and Care Sciences; University of Gothenburg Centre for Person-Centred Care,
**Primary Subject Heading**:	Health economics
Secondary Subject Heading:	Paediatrics, Intensive care
Keywords:	HEALTH ECONOMICS, PAEDIATRICS, RESPIRATORY MEDICINE (see Thoracic Medicine), Chronic airways disease < THORACIC MEDICINE, Paediatric thoracic medicine < THORACIC MEDICINE

SCHOLARONE™
Manuscripts

I, the Submitting Author has the right to grant and does grant on behalf of all authors of the Work (as defined in the below author licence), an exclusive licence and/or a non-exclusive licence for contributions from authors who are: i) UK Crown employees; ii) where BMJ has agreed a CC-BY licence shall apply, and/or iii) in accordance with the terms applicable for US Federal Government officers or employees acting as part of their official duties; on a worldwide, perpetual, irrevocable, royalty-free basis to BMJ Publishing Group Ltd ("BMJ") its licensees and where the relevant Journal is co-owned by BMJ to the co-owners of the Journal, to publish the Work in this journal and any other BMJ products and to exploit all rights, as set out in our licence.

The Submitting Author accepts and understands that any supply made under these terms is made by BMJ to the Submitting Author unless you are acting as an employee on behalf of your employer or a postgraduate student of an affiliated institution which is paying any applicable article publishing charge ("APC") for Open Access articles. Where the Submitting Author wishes to make the Work available on an Open Access basis (and intends to pay the relevant APC), the terms of reuse of such Open Access shall be governed by a Creative Commons licence – details of these licences and which Creative Commons licence will apply to this Work are set out in our licence referred to above.

Other than as permitted in any relevant BMJ Author's Self Archiving Policies, I confirm this Work has not been accepted for publication elsewhere, is not being considered for publication elsewhere and does not duplicate material already published. I confirm all authors consent to publication of this Work and authorise the granting of this licence.

For peer review only

SYSTEMATIC REVIEW OF THE HEALTHCARE COST OF BRONCHOPULMONARY DYSPLASIA

AUTHORS

Jhangir Humayun[1,2], gushumjh@student.gu.se

Chatarina Löfqvist[1,2,3] chatarina.lofqvist@gu.se

David Ley[4], david.ley@med.lu.se

Ann Hellström[3], ann.hellstrom@medfak.gu.se

Hanna Gyllensten[1,2] hanna.gyllensten@gu.se

Author affiliations

[1] Institute of Health and Care Sciences, Sahlgrenska Academy, University of Gothenburg, Box 457, SE-405 30 Gothenburg, Sweden.

[2] Centre for Person-Centred Care (GPCC), University of Gothenburg, Box 457, SE-405 30 Gothenburg, Sweden.

[3] Department of Clinical Neuroscience, Institute of Neuroscience and Physiology, Sahlgrenska Academy, University of Gothenburg, Gothenburg, Sweden.

[4] Department of Pediatrics, Institute of Clinical Sciences Lund, Lund University and Skåne University Hospital, Lund, Sweden.

Contact, corresponding author

Hanna Gyllensten, Institute of Health and Care Sciences, Sahlgrenska Academy, University of Gothenburg, Postal Address: Box 457, SE-405 30 Gothenburg, Sweden, E-mail: hanna.gyllensten@gu.se. Phone: +46-(0)70-748 24 12

Word count (excluding title page, abstract, references, figures and tables): 4329

For peer review only

Abstract

Objectives

To determine the costs directly or indirectly related to bronchopulmonary dysplasia (BPD) in preterm infants. The secondary objective was to stratify the costs based on gestational age and/or birth weight.

Design

Systematic literature review

Setting

PubMed and Scopus were searched on 3 Feb 2020. Studies were selected based on eligibility criteria by two independent reviewers. Included studies were further searched to identify eligible references and citations.

Two independent reviewers extracted data with a pre-specified data extraction sheet, including items from a published checklist for quality assessment. The costs in the included studies are reported descriptively.

Primary outcome measure

Costs of BPD.

Results

The 13 included studies reported the total costs or marginal costs of BPD. Most studies reported costs during birth hospitalization (cost range: Int$21,392–1,094,509 per child, equivalent to €19,103–977,397, in 2019) and/or during the first year of life. One study reported costs during the first two years; two other studies reported costs later, during the pre-school period; and one study included a long-term follow-up. The highest mean costs were associated with infants born at extremely low gestational ages. The quality assessment indicated a low risk of bias in the reported findings of included studies.

Conclusions

This study was the first systematic review of costs associated with BPD. We confirmed previous reports of high costs and described the long-term follow-up necessary for preterm infants with BPD, particularly infants of very low gestational age. Moreover, we identified a need for studies that estimate costs outside hospitals and after the first year of life.

Registration

The protocol is registered in PROSPERO (registration number CRD42020173234).

For peer review only - http://bmjopen.bmj.com/site/about/guidelines.xhtml

ARTICLE SUMMARY

Strengths and limitations of this study

- This is the first systematic review specifically focused on costs resulting from bronchopulmonary dysplasia after preterm birth.
- Authors searched systematically through PubMed and Scopus, which are appropriate databases for the investigation topic, and also reviewed reference lists and citations of the relevant papers to identify any additional studies.
- The main limitation was the lack of search for unpublished and/or grey literature.

For peer review only

For peer review only - http://bmjopen.bmj.com/site/about/guidelines.xhtml

INTRODUCTION

Bronchopulmonary dysplasia (BPD) is a severe lung condition that can affect new-borns. It is prevalent in premature infants, particularly infants that require oxygen therapy. Infants born more than ten weeks preterm are more susceptible to BPD because they often require a long treatment period with different types of breathing support, such as nasal continuous positive airway pressure, ventilators, different bronchodilators, and steroids.[1]

The definition of BPD has varied considerably over time. The condition was first described by Northway et al., in 1967, as a radiographic pattern of lung injury observed in prematurely born infants after the use of extensive mechanical ventilation.[2] Considerable strides in care have been made for these infants over the past decades, including, but not limited to, the use of antenatal steroids, surfactant therapy, vitamin A supplementation, and caffeine treatment.[3-5] The administration of antenatal steroids and exogenous surfactant has dramatically reduced the severity of respiratory distress syndrome, and consequently, increased the survival of preterm infants.[6] Before the widespread use of antenatal steroids and surfactant, BPD in preterm infants was mainly attributed to lung injury caused by barotrauma and oxygen toxicity.[6] The pathogenesis of the BPD observed currently, in the post surfactant era, is multifactorial; various causes have been found, including extreme prematurity, infection, nutritional deprivation, and the need for prolonged mechanical ventilation.[7 8] Short-term management thus includes, preferably non-invasive, respiratory support titrated to specific oxygen saturation ranges, exogenous surfactant therapy, respiratory stimulants such as caffeine, corticosteroids to reduce inflammation, and inhaled nitric oxide against pulmonary hypertension. In contrast, long-term management includes a larger range of products to handle persistent and severe chronic respiratory disease and related comorbidities.[9] It is imperative to understand this new form of BPD and its effects during infancy and beyond because the survival of preterm infants is increasing.[10]

Currently, BPD is commonly defined as a condition that requires oxygen administration beyond 36 weeks postmenstrual age (PMA). BPD severity is classified as light, moderate, or severe depending on the required respiratory support and inhaled oxygen concentration. Co-morbidities and maternal infections increase the risk of developing severe BPD after preterm birth. Disease severity varies considerably; for example, in mild cases, infants might only present with an accelerated respiratory rate; but mechanical ventilation may be required for a prolonged period in severe cases.[1] Indeed, serious conditions often require continuous oxygen therapy for several months.

The incidence of BPD seems to be inversely related to the gestational age (GA, reported in weeks) at birth. Studies that included infants born at low GAs have reported higher incidences of BPD.[11] The National Institute for Child Health and Human Development neonatal network periodically reports the incidence of BPD. They define BPD as a condition that requires oxygen administration at 36 weeks PMA. They reported BPD incidences of 52% in infants with birth weights of 501 - 750 g, 34% in infants with birth weights of 751-1000 g, and 15% in infants with birth weights of 1001-1200 g. Among infants that weighed 1201-1500 g, the incidence was as low as 7%.[12]

Foetal growth restriction is commonly defined as low birth weight, corrected for GA. Foetal growth restriction has a clear impact on the incidence and severity of BPD. Studies have shown that among infants born at a similar GA, infants that were small for their GA (SGA) had a higher BPD incidence than infants that had normal birth weights for their GA.[11] Globally, the survival rates of extremely preterm infants (GA <28 weeks) have varied considerably, according to the economic setting. Highly developed countries have reported extremely preterm infant survival rates as high as ~90%, but it

For peer review only - http://bmjopen.bmj.com/site/about/guidelines.xhtml

can be as low as ~10% in low-income settings.[13] Similarly, the incidence of BPD in preterm infants varies considerably with the degree of economic development in the country of birth.

The effects of BPD on infant health extend beyond the initial hospitalization. Infants with BPD are hospitalized to a much greater extent than infants without BPD.[14] Lung function abnormalities, such as small airways, often persist for an extended period. Long-term effects, such as reduced lung function and obstructive airway disease, are more prevalent among individuals born prematurely than their born full-term peers. Among preterm infants, those with BPD exhibit a greater need for respiratory medications than those without BPD.[14] Neurodevelopment can be severely affected by BPD. Infants with BPD have an increased rate of abnormal neurodevelopment, including cognitive impairments and cerebral palsy. In severe cases of BPD, movement morbidities can occur, like chorea and akathisia.[15]

An economic evaluation is essential for aiding decision-making bodies in prioritizing healthcare funding effectively and understanding the associated long-term costs. A review [15] was previously published on the healthcare and societal cost of BPD, but the search was not systematic; the authors handpicked all the articles. To our knowledge, no systematic review has been published that discussed the healthcare costs of BPD. Thus, there was a need to compile and present the available data on this subject in a systematic review.

The present review aimed to present all available data that pertained to the costs related to BPD, directly or indirectly, in preterm infants. The secondary objective was to stratify those costs based on the GA or birth weight of the infant.

METHODS

This study was conducted according to the Preferred Reporting Items for Systematic Review and Meta-Analysis (PRISMA) recommendations.[5] The protocol is registered in PROSPERO (registration number CRD42020173234).

Search strategy

In collaboration with personnel of the Medicine Library at the University of Gothenburg, the authors determined that the PubMed and Scopus databases were the best choice for this review. The search was conducted on 3 Feb 2020 in both databases. Search terms (Appendix, Table S1) were drafted and discussed individually for both databases. The search was performed, and articles were included according to the criteria listed below. No restrictions were applied for publication date, country of origin, or language. Included studies were further searched to identify eligible references and citations.

Study selection

We included observational and interventional studies that investigated the costs associated with BPD. The study population comprised preterm human infants with BPD. Two reviewers (JH and HG) independently screened the titles and abstracts of identified studies to assess their eligibility for inclusion. After reviewing the titles and abstracts, duplicates were removed. Then, the reviewers obtained full texts of potentially eligible articles and assessed them independently to determine which articles met the predetermined inclusion criteria. When the reviewers were uncertain of the eligibility of a study, a third reviewer (CL) participated in the decision.

For peer review only - http://bmjopen.bmj.com/site/about/guidelines.xhtml

Data extraction and quality assessment

To ensure essential data were extracted consistently from relevant articles, we created an extraction table. Data from eligible studies were independently extracted by two reviewers (JH and HG). Any differences were resolved by discussion and consultation with a third reviewer (CL). The predesigned data extraction table included but was not limited to the following data: the name of the first author, year of publication, study design, location, sample size, healthcare costs, and description of the costs included. When available, healthcare costs were stratified according to the GA at birth or birth weight. The two authors independently assessed the quality of the selected studies, based on predetermined quality assessment criteria for health economic studies (the CHEC-list) described previously by Evers et al.[16] Although designed for economic evaluations, and this CHEC-list has previously been used in similar studies.[17 18]

Analyses

Costs were converted to USD, based on the purchasing power parities of each year.[19] Then, the costs were inflated to 2019 values with the Organisation for Economic Co-operation and Development (OECD) inflation factor.[20] Costs were analysed, based on the chronological age of the participants included in each study and by their GA or birth weight.

Patient and public involvement

This project did not include patient or public involvement in developing the research questions, design, conduct, choice of outcome measures, or recruitment.

RESULTS

The initial search results yielded 274 articles. After removing duplicates (Figure 1), 262 unique articles were screened, and 195 were excluded based on the abstract or title. Among the 66 full articles screened, 13 [21-33] unique studies met the inclusion criteria. The hand search of references and citations of included studies did not identify any additional eligible studies. The included studies were conducted in 1981-2015 (Table 1), in the US (7 studies)[22 23 28 30-33], UK (2 studies)[25 26], Spain[29], Canada[27], Turkey[24], and France[21].

Six studies[22 23 25 26 28 29] defined BPD as oxygen dependence beyond 28 days after birth (table 1), and two of these studies[28 29] also classified BPD as mild/moderate/severe, based on whether oxygen was administered at 36 weeks PMA, which, according to Jobe and Bancalari,[34] indicated moderate/severe BPD. Three studies[24 30 33] used only the 36 weeks PMA oxygen use definition, while three additional studies[27 31 32] defined BPD based on the ICD.9 (770.7) code from a registry, without reference to GA or time since birth at diagnosis. The oldest included study[21], was based on hospital data, not stating whether they used ICD codes or what time period they used for the oxygen requirement. However, their definition was consistent with the classical definition of BPD,[2], which was based on clinical signs. In two studies[22 23] the definition of BPD required a chest x-ray consistent with BPD, in addition to the oxygen dependence beyond 28 days after birth requirement.

For peer review only - http://bmjopen.bmj.com/site/about/guidelines.xhtml

Table 1: Overview of studies included in this review

#	First author (year)	Country (study period) Setting	Study design	BPD definition	Sample size	Time horizon: age of children	Healthcare cost per child with BPD	Cost perspective: cost inclusion
1	Lapcharoensap (2020)[33]	US (2008-2011) California Perinatal Quality Care Collaborative	Retrospective cohort	Oxygen requirement >36 weeks PMA	BPD: 2,696 Controls: 5,302	1 year: from birth, including index hospitalization	$442,468	Hospital: Direct hospital costs calculated from charges in discharge records using cost-to-charge ratio
2	Mowitz (2019)[32]	US (2009-2015) Premier Perspective Database	Retrospective cohort	ICD code	BPD: 4,904 Controls: 7,113	Short-term: Index hospitalization	$225,204	Hospital: Direct hospital costs, from billed services
3	Álvarez-Fuente (2017)[29]	Spain (2013) SEN1500 database	Retrospective cohort: Model base case for minimum cost of BPD using weighted costs	Oxygen dependence >28 days after birth (also >36 weeks for severity assessment)	BPD: 2,628	2 years: from birth, including index hospitalization	€45,049-118,760	Health system: Hospital costs and follow-up cost; inpatient costs were estimated using diagnosis related group weights, price lists were used for outpatient visits and immunization
4	Patel (2017)[30]	US (2009-2012) Rush University Medical Center NICU, Chicago/Illinois	Prospective cohort (compare own mothers milk v. formula)	Oxygen requirement >36 weeks PMA	BPD: 77 Controls: 177	Short-term: Index hospitalization	$269,004 (Median)	Hospital: Hospital costs, overhead, and physician payments from a financial data repository with costs for chargeable items
5	Stevens (2017)[31]	US (2008-2011) The California Office of Statewide Health Planning and Development (OSHPD) database	Retrospective cohort: Regression model to identify the cost incurred by infants with specific conditions	ICD code	Total: 2,021,013 BPD: 2189	1 year: from birth, including index hospitalization	$133,140 Cost increase = 22.4%	Hospital: Commercial claims data from the region corrected to national costs using register data from the Healthcare Cost and Utilisation Project data set
6	Johnson (2013)[28]	US (2005-2009) Rush University Medical Center NICU, Chicago/Illinois	Retrospective cohort	Oxygen dependence >28 days after birth (also >36 weeks for severity assessment)	BPD: 230 Controls: 195	Short-term: Index hospitalization	$103,151	Hospital: Hospital costs excluding physician fees from a financial data repository with costs for chargeable items

For peer review only

For peer review only - http://bmjopen.bmj.com/site/about/guidelines.xhtml

7	Landry (2012)[27]	Canada (1983-2008) MED-ECHO and RAMQ databases, Quebec	Retrospective cohort	ICD code	BPD: 773 Controls: 2669	16 - 25 years: from birth, after the index hospitalization	C$13,472	Health system: Hospitalization follow-up cost and prescription drugs; inpatient costs were estimated using a case mix approach, billed fees were used for outpatient visits and drugs
8	Greenough (2011)[26]	UK (2002-2005) Previously presented cohort, hospitals not stated[35]	Retrospective cohort	Oxygen dependence >28 days after birth	BPD: 160	2 years: 5-7 years of age	£523-705 (Median)	Health system: Hospital admission costs**, follow-up cost, and prescription drugs; price lists were used for admissions and drugs, unit costs based on wages and time used by clinicians for outpatient visits
9	Greenough (2006)[25]	UK (1999-2002) Previously presented cohort, hospitals not stated[35]	Retrospective cohort	Oxygen dependence >28 days after birth	BPD: 190	2 years: 2-4 years of age	£950-3,054 (Median)	Health system: Hospital admission costs**, follow-up cost, and prescription drugs; price lists were used for admissions and drugs, unit costs based on wages and time used by clinicians for outpatient visits
10	Akman (2002)[24]	Turkey (1999-2000) Marmara university hospital, Istanbul	Retrospective cohort	Oxygen requirement >36 weeks PMA	BPD: 72	Short-term: Index hospitalization	$14,810 (Marginal cost)	Hospital: Hospital charges collected from the hospital Accounting Department, including physician and nursing services, laboratory and radiology tests and medical supplies
11	Miller (1998)[23]	US (1990) Tulane University Medical Centre, Louisiana	Retrospective cohort	Oxygen dependence >28 days after birth and chest x-ray	BPD:89	4.5 years: from birth, after the index hospitalization	$19,034*	Payer (third party and parents): Direct health costs (registered charges, or costs translated to charges using cost-to-charge ratios), non-health costs (expenses), and indirect cost (lost income) to families
12	McAleese (1993)[22]	US (1981-1989) Dartmouth-Hitchcock Medical Centre, New Hampshire	Retrospective cohort	Oxygen dependence >28 days after birth and chest x-ray	BPD: 59	2 months-2.7 years: from birth, including index hospitalization	Initial hospitalization = $197,668 Home care = $4,262-68,136 (Medians) Indirect costs =	Payer (third party and parents): Hospital charges for hospitalization, charges for follow-up cost and home care equipment. expenses and travel costs, and indirect cost (lost income) to families
13	Monset-Couchard (1984)[21]	France (1981) Port-Royal Intensive Care Unit, Paris	Retrospective cohort	Hospital data	Not stated	Short-term: Index hospitalization	₣336,545	Hospital: Unit costs for hospitalizations, travel charges (including ambulance and air travel)

For peer review only

Unless otherwise stated, the costs are the mean costs reported in the original paper. Abbreviations: C= Controls; C$= Canadian dollar; ₣= French franc; ICD= international classifications of diseases; PMA= post-menstrual age.

*Mean cost was computed based on the data available in the article

**** Does not include index hospitalization since the year of birth is not included in the time period of each study.**

For peer review only

For peer review only - http://bmjopen.bmj.com/site/about/guidelines.xhtml

Risk of bias in included studies

Potential sources of bias included the lack of participant characteristics in some of the included studies. Additionally, four studies[21-24] lacked information about potential conflicts of interest for the participating researchers (Supplemental table S2). Only one study[31] conducted a sensitivity analysis of their results. None of the included papers clearly stated the research question, but it could be gleaned from the objective of each study. Three studies[25 26 30] included comparisons between groups or long-term outcomes, and no study included an evaluation of health outcomes. Thus, some of the CHEC-list items were not suitable for evaluating these studies. Checklist items judged relevant were largely covered by the included papers. Only three papers[21 23 24] had more than three items missing, and the items not covered throughout the papers mainly were related to lack of a well-defined research question and lack of sensitivity analyses. Overall, the assessment suggests a low risk of bias in the included papers.

Total costs for BPD

A majority of studies[21 22 24 28-30 32 33] (Table 2) reported costs during the birth hospitalization and/or the first year of life (8 studies). One study[29] reported follow up costs during the first two years of life, including birth hospitalisation. Two other studies[25 26] reported two-year follow-up costs for age groups 2-4 and 5-7 years, respectively. Two studies[23 27] included a long-term follow up, with an average follow-up of 4.5 years and 19.3 years, respectively (Figure 2).

Table 2: Costs for infants with BPD

**Reference** *subgroups reported in the paper*	**Total cost (Int$)**	**Cost of index Hospitalization (Int$)**	**Cost of other care (Int$)**	**Cost increase over controls due to BPD, Int$ (%)**
**1. Lapcharoensap (2020)[33]**	477,901	466,216	Rehospitalization: 46,948	246,284 (106%)
≤ 24 weeks	*507,638*			*118,808 (35%)*
25- 26 weeks	*453,633*			*129,609 (40%)*
27-28 weeks	*351,026*			*124,209 (55%)*
> 28 weeks	*313,223*			*151,211 (93%)*
**2. Mowitz (2019)[32]**	see index hospitalization	242,995		58,381 (31%)
≤ 24 weeks	see index hospitalization	*326,962*		*26,975 (9%)*
25- 26 weeks	see index hospitalization	*247,901*		*32,370 (15%)*
27-28 weeks	see index hospitalization	*190,983*		*36,686 (24%)*
> 28 weeks	-	-		-
**3. Álvarez-Fuente (2017)[29]**	74,618 - 196,713		2-year follow-up:	-
<750g	*196,713*	*182,627*	*14,084*	-

For peer review only - http://bmjopen.bmj.com/site/about/guidelines.xhtml

750- 999g	*149,071*	*134,986*	*14,084*	-
1000-1249g	*73,237*	*60,534*	*14,084*	-
> 1249 g	-	-	-	-
**4. Patel (2017)**[30]	see index hospitalization	290,546*<br>Marginal cost:<br>45,287*		164,092*
**5. Stevens (2017)**[31]	148,251	-	-	(22.4%)
**6. Johnson (2013)**[28]	see index hospitalization	122,983		69,969 (132%)
<750g				*51,639*
750- 999g				*41,196*
1000-1249g				*29,317*
> 1249 g				*23,810*
**7. Landry (2012)**[27]	12,973/year		16-25 year follow-up:<br>12,973/year	2,651/year (26%)
**8. Greenough (2011)**[26]<br>(2 years at 5-7 years of age)	Home oxygen:<br>1,135*<br>No oxygen:<br>842*		Home oxygen:<br>1,135*<br>No oxygen:<br>842*	
**9. Greenough (2006)**[25]<br>(2 years at 2-4 years of age)	Home oxygen:<br>5,556*<br>No oxygen:<br>1,728*		Home oxygen:<br>5,556*<br>No oxygen:<br>1,728*	
**10. Akman (2002)**[24]	see index hospitalization	21,392	-	15,116 (241%)
**11. Miller (1998)**[23]	34,692		4.5-year follow-up<br>Inpatient services:<br>25,268<br>Outpatient services:<br>6,625<br>Indirect costs:<br>2800	
**12. McAleese (1993)**[22]	see index hospitalization	387,085	Home care, nurse:<br>133,427*<br>Home care, no nurse:<br>8,346*<br>(including indirect costs)	
**13. Monset-Couchard (1984)**[21]	see index hospitalization	1,094,509		

For peer review only

Values are the mean costs unless otherwise stated. *Median cost; Int$ = The cost after converting to purchasing power parities, then inflating to the 2019 value

Index hospitalization

The mean costs during the index hospitalization ranged from Int$21,392 to Int$1,094,509 (Figure 3). For five studies[22 28 30 32 33] conducted in the USA, the range of index hospitalisation costs was Int$122,983-466,216. If only including those four US studies limiting inclusion to severe BPD, the range is Int$242,995-466,216.[22 30 32 33]

Hospital readmissions in the first two years of life

One study[33] analysed rehospitalizations among infants with BPD. They showed that rehospitalizations among infants with BPD occurred more than twice at frequently and incurred higher costs than rehospitalizations among their peers without BPD. Rehospitalizations were required in 25.1% of infants with BPD, and the mean cost was estimated to be Int$46,948 during the first year of life.[33] One study[29] estimated that the cost associated with BPD for a mean follow-up of the first two years of life was Int$14,084.

Follow-up costs and home care

Two studies[25 26] reported costs during a specific age, thus not including the costs occurring at birth and during the first years of life: Among children with BPD, the median costs during two years of follow-up for ages 2-4 were estimated at Int$5,556 for those that required home oxygen after the initial discharge, compared to Int$1,728 for those that did not require home oxygen after the initial discharge from the hospital.[25] Furthermore, the median costs during two years of follow-up for ages 5-7 were Int$1,135 for those that required home oxygen, compared to Int$842 for those that did not require home oxygen after the initial discharge.[26] Children initially requiring oxygen during a longer period also required more resources at older ages.

Other studies reported costs from birth and onwards, but excluding the index hospitalisation: One study[23] reported that the median follow-up cost of healthcare for children with BPD 4.5 years after initial hospitalisation was Int$25,268 for inpatient care and Int$6,625 for outpatient services. One study[27] analysed the cost of healthcare for children with BPD during a follow up for ages 16-25 years; they reported that the annual cost was Int$12,973 per person.

Moreover, one study[22] reported that the median cost of home healthcare (including indirect costs for lost wages) was Int$133,427 with a nurse and Int$8,346 without a nurse. Thus, costs for home healthcare were largely dependent on the possibilities to employ a nurse based on private insurance payments and other funds.

Indirect costs

One study[23] reported an indirect cost of Int$2,800 during 4.5 years after the initial hospitalisation. One study[22] reported indirect costs as part of the home care costs, and for the 30 families reporting the loss of wages, this corresponded to a range of Int$516-44,105 per family (with 9-577 days of lost income) in 2019 value.

Increase in costs due to BPD

In four of the included studies[24 28 32 33], BPD incurred, on average, 31 – 241% extra expenses in healthcare. One study[27] from Canada reported that the mean follow-up cost increased by Int$2,651/year, a 26% increase over the cost for preterm peers without BPD.

In four studies[28 29 32 33] that reported costs according to GA categories, the cost ranges were: Int$196,713 – 507,638 for those born at or before GA 24 weeks/<750 g; Int$149,071 – 453,633 for those born at GA 25-26 weeks/750-999

For peer review only

g; and Int$73,237 – 351,027 for those born at GA 27-28 weeks/1000-1249 g. One study[33] reported a cost of Int$313,223 for those born at GA 29 weeks or later/>1249 g (Figure 3). Higher cost increases were reported for infants with BPD born at higher GAs than those born at lower GAs. For example, studies reported that the mean costs for infants with BPD increased by 9 – 35% for those born at or before GA 24 weeks; by 15 – 35% for those born at GA 25-26 weeks; and by 19 – 35% for those born at GA 27-28 weeks. One study[33] reported a 93% cost increase for infants born at GA 29 weeks or later. The mean total cost increase for infants with BPD, stratified by GA, is shown in Figure 4.

DISCUSSION

The included studies reported high costs for preterm infants with BPD during the initial hospitalization after birth (range: Int$21,392–1,094,509 per child, equivalent to €19,103–977,397, in 2019). However, looking at only four studies conducted in one country and similar inclusion criteria, the range was Int$242,995-466,216. Moreover, the studies reported high use of resources associated with BPD during the following years, up to school age. The highest mean costs were associated with infants born at extremely low GAs.

Several prior reviews and modelling studies in this field [15, 36] have reported costs, but they based study inclusions on expert opinions and targeted searches. Therefore, the present study provided an overview of all the evidence available on costs for BPD, with expected low risk of bias in included studies based on the quality assessment tool. However, the main limitation of this study was the lack of searches for identifying grey literature and not performing analysis of publication bias. However, we did perform an initial search based on references identified in, e.g., modelling studies and reviews, when we designed the search strategy to identify all the relevant publications with our search strategy. We found several studies that addressed costs of interventions, such as immunization or infection treatment for infants with BPD, but in those cases, the costs were not included for BPD *per se*. Our search was explicitly tailored to identify articles on BPD; thus, we excluded articles that used an alternate term, like chronic lung disease (CLD). We identified very relevant articles that were relevant, considering the great need for quality long-term healthcare for preterm infants with BPD.

Moreover, we initially intended to conduct a meta-analysis of results, but we found that, in the separate papers, the costs reported differed too extensively in content and time-horizons to support that type of analysis. For the same reason, we could not conduct a formalized synthesis of the results. Moreover, the recently published guidelines for a data synthesis without a meta-analysis [37] were unsuitable for this study; instead, they were more suited to the synthesis of effect estimates for intervention studies. Consequently, the analysis herein was descriptive.

Another factor making comparisons between results in included studies was that, in the BPD literature, the operational definition of BPD has varied since the condition was first discovered.[9] Thus, the extent of variability we encountered in the included papers (detailed in the Results section) might have affected the selection of infants, which might have influenced the healthcare costs. It is also important to note that the costs of BPD reported in the selected studies was based on the costs incurred by oxygen administration. However, the full economic impact of BPD should also include the costs associated with preventing BPD development in infants at risk. This same limitation applies to all retrospective studies that based the definition of BPD on ICD codes and clinical assessments because infants who successfully avoided BPD development would not be classified as BPD, either with the codes or in clinical assessments.

Regarding the cost estimations, the majority of the included articles focused on the initial hospitalization. However, after the initial hospitalization, many infants with BPD require rehospitalization during the first years of life due to respiratory illnesses.[33] Infants born prematurely often display symptoms and signs of obstructive airway disease that persist for prolonged periods; thus, it is crucial to implement standardized, regular follow-up schedules.[38]

Our findings showed how the cost estimates for BPD varied across different countries and during different periods. Table 2 shows a large cost discrepancy among different countries, e.g., Turkey and the USA, which several factors might explain. First, BPD treatment programs might differ with different healthcare providers, during different years, and in different countries.[39] For example, articles that investigated the costs of BPD in the USA reported much higher

For peer review only - http://bmjopen.bmj.com/site/about/guidelines.xhtml

costs than those reported in other countries; this discrepancy might partly be explained by the higher cost of healthcare, in general, in the USA.[40] Among studies conducted in the USA, the study by Johnson and colleagues[28] stands out compared to lower costs for the initial hospitalisation, possibly due to the exclusion of physician fees or from the BPD definition being inclusive also of milder cases. Second, the included studies spanned a long time period, where healthcare will have changed dramatically due to technological development. It is clear from the data provided that care of infants with BPD has been resource intense throughout the period, and the inflated estimates indicate the current value of the resources used at that time, but it is difficult to compare costs between a study from the early 1980s to those conducted in 2010s. The patient group will look very different, with more infants born very early surviving to develop BPD. In addition to the potential effects of different BPD definitions, infants with BPD frequently have other comorbidities, such as brain injury and retinopathy of prematurity (ROP), which can lead to functional impairments that impact the cost of healthcare.[41] The cost of BPD without any comorbidity can be difficult to differentiate from the total cost, including comorbidities. For example, comorbidities like pulmonary hypertension (PH) in infants with BPD can significantly increase healthcare costs due to , more extended hospitalizations and a higher likelihood of requiring home respiratory support.[42] Third, healthcare quality can differ between different healthcare providers, resulting in cost differences. Fourth, some of the included articles omitted data for some costs, e.g., the cost of physicians.[28] Finally, as previously discussed, an SGA condition can exacerbate the severity of BPD; this condition was not considered in the cost calculations because the included articles did not report the combined effects of GA and BW.

Two studies compared healthcare costs between preterm infants with BPD and their preterm peers without BPD. Those data enabled a calculation of the cost increase due to BPD. The results indicated that the cost differences were higher among infants born at lower GAs. This finding was consistent with cost estimates for other diagnoses in the same patient groups, such as infants born to mothers with preeclampsia.[31] Among our included studies, the study by Johnson et al.,[28] estimated marginal costs in infants with BPD compared to infants of the same weight without any of four major comorbidities (sepsis, brain injury, necrotizing enterocolitis [NEC], or BPD). They found that the cost increase was more prominent among the smallest infants (<750 g). Lapcharoensap et al.,[33] compared infants with BPD to all other infants of the same age (no limitation on comorbidities). They reported the opposite relationship, where,more considerable cost differences were observed among those born at higher GAs. This relationship might be expected because, in the reference groups, infants born at higher GAs can be expected to have fewer of the major comorbidities than infants born at lower GAs; thus, the high GA group would show a larger cost difference between infants with and without BPD. This effect was also reported by Mowitz et al.,[32], although the detailed cost data were not available. They reported that among all children (regardless of comorbidity), the cost increase due to BPD was largest for infants born at a higher GA; however, when infants with BPD were compared to those without BPD, ROP, or intraventricular haemorrhage (IVH), the cost increase was similar independent of GA. In contrast, the study by Johnson et al. showed a clear trend towards a higher cost difference due to BPD among the smallest infants. The differences between the two latter studies might be explained by the fact that the comorbidities excluded in the study by Johnson et al.[28] (the comparison group had no sepsis, brain injury, NEC, or BPD) were different from those excluded in the study by Mowitz et al.[32] (the comparison group had no ROP, IVH, or BPD).

It is clear from the included studies that infants and children with BPD often required long-term follow-ups. This requirement affects the lives of these infants in various direct and indirect ways, which have not been adequately investigated. A recent modelling study,[36] based on a targeted literature search, estimated the life-time burden of BPD for infants born ≤28 weeks GA. That study explored how BPD severity and major complications affected other aspects of life, like clinic visits for wheezing episodes, asthma exacerbations, psychiatric illnesses, et cetera. As expected, the model found that BPD had a large impact on healthcare use, quality-of-life, and survival, but it also showed that the degree of impact was largely dependent on BPD severity. Accordingly, improvements in BPD therapy might substantially reduce the impact of BPD.[36] However, as those authors acknowledged, the model could only use the available data. Thus, it was limited by, e.g., data on each complication separately (not accounting for concomitant diagnoses in one patient, potentially increasing costs more than expected from single diagnosis studies). That study also reported that the healthcare cost of BPD for the first year of life was C$197,100, which was consistent with the estimates of more recent studies included in this review.

For peer review only - http://bmjopen.bmj.com/site/about/guidelines.xhtml

The findings from both our systematic review and the previous modelling study [36] suggested a need for more research on the long-term economic impact of BPD. Preferably, those studies should also include costs outside the health system, such as costs due to changes in parent productivity and costs for schooling and daycare. In countries like the Nordics, where much information about resource use is available in national and/or regional registers collected for administrative purposes and available for research, many relevant resources can be studied in retrospective register studies.[43 44] However, some types of information are less likely to be included in registers, such as informal care provided by parents and other relatives. Thus, studies using prospective and retrospective data collection methods should add important knowledge on the long-term costs associated with BPD.

Moreover, studies should consider the costs related to the combined impact of complications. It has been argued that studies with a societal perspective can better identify potential inequities and inefficient use of society's resources.[45] One can speculate that one reason for the paucity of cost studies on BPD is that new drug developments have slowed after the success of surfactant therapy, which was introduced in the early 1990s.[46] Although several advances in the care of preterm infants and BPD have improved survival and symptom reduction,[47] these care applications and treatments have not, to the same extent, been subjected to economic and cost assessments.[48] To minimize the growing burden of BPD, it is vital to prevent prematurity in the first place, and in the second place, to develop new treatments for prematurity-related diseases.[49] The ongoing research on the administration of stem cells, anti-inflammatory agents, and IGF-I/IGFBP-3 among extremely pre-term infants[9] may spark a renewed interest in how resources are best used for this patient group.

This systematic review confirmed previous reports that BPD in preterm infants was associated with high costs and required long-term follow-ups. Our primary finding was that the highest costs occurred among infants born at lower GAs and that this relationship was difficult to detect unless an appropriate control group was used. Moreover, we identified a shortage of studies that estimated, in particular, costs that occurred outside hospitals and after the first year of life. Further research on BPD is required to understand the long-term consequences regarding healthcare costs and the social aspects of BPD.

For peer review only

For peer review only - http://bmjopen.bmj.com/site/about/guidelines.xhtml

Funding statement

JH was financed by the University of Gothenburg Centre for Person-Centred Care (GPCC) teaching assistant program and the Mary von Sydow Foundation. CL was financed by the University of Gothenburg Centre for Person-Centred Care (GPCC). DL was financed by the Swedish Research Council (#2018-00770). AH was supported by the Swedish Research Council (#2016-01131), the Gothenburg Medical Society, De Blindas Vänner, and Government grants under the ALF agreements (ALFGBG-717971 and ALFGBG-812951), and The Wallenberg Clinical Scholars. HG was financed by the Swedish Research Council (#2016-01131) and the Swedish Research Council (#2018-00770). The funders played no role in the design of the study or writing the protocol.

Competing interests statement

JH report no competing interests. CL holds stocks in Premalux AB. DL and AH hold stock/stock options in Premalux AB, and have received consulting fees from Takeda Inc. HG is employed part-time by Statfinn and EPID Research (part of IQVIA), which is a contract research organisation that performs commissioned pharmacoepidemiological studies, and thus its employees have been and currently are working in collaboration with several pharmaceutical companies.

For peer review only

Contributions

All authors contributed to the design of the study. JH, CL, and HG designed the database search and data extraction methods. JH and HG undertook the literature search, assessed studies for eligibility, and extracted data. In case of disagreement, assessments were made in discussion with CL. DL and AH contributed clinical expertise on preterm infants and morbidity. JH, CL and HG discussed the data and interpreted the results. JH and HG drafted the manuscript. All authors critically reviewed and approved the final manuscript.

Acknowledgements

This systematic review was part of the Less is More project (Swedish Research Council grant number #2018-00770), a longitudinal study of clinical and economic outcomes in children born at very low gestational age. This study thus contributes with an understanding of the current knowledge and important gaps to the Less is More study design for long-term follow-up and economic evaluation.

Data Availability Statement

All data relevant to the study are included in the article. Original data is available in the reviewed publications which are all cited. Additional data from the data extraction performed are available upon reasonable request from the corresponding author.

Ethical Approval Statement

Not applicable.

For peer review only - http://bmjopen.bmj.com/site/about/guidelines.xhtml

References

1. Hwang JS, Rehan VK. Recent Advances in Bronchopulmonary Dysplasia: Pathophysiology, Prevention, and Treatment. *Lung* 2018;**196**(2):129-38.
2. Northway WH, Jr., Rosan RC, Porter DY. Pulmonary disease following respirator therapy of hyaline-membrane disease. Bronchopulmonary dysplasia. *N Engl J Med* 1967;**276**(7):357-68.
3. Yeh TF, Lin HC, Chang CH, et al. Early intratracheal instillation of budesonide using surfactant as a vehicle to prevent chronic lung disease in preterm infants: a pilot study. *Pediatrics* 2008;**121**(5):e1310-8.
4. Tyson JE, Wright LL, Oh W, et al. Vitamin A supplementation for extremely-low-birth-weight infants. National Institute of Child Health and Human Development Neonatal Research Network. *N Engl J Med* 1999;**340**(25):1962-8.
5. Schmidt B, Roberts RS, Davis P, et al. Caffeine therapy for apnea of prematurity. *N Engl J Med* 2006;**354**(20):2112-21.
6. Bancalari E, del Moral T. Bronchopulmonary dysplasia and surfactant. *Biol Neonate* 2001;**80 Suppl 1**:7-13.
7. Rojas MA, Gonzalez A, Bancalari E, et al. Changing trends in the epidemiology and pathogenesis of neonatal chronic lung disease. *J Pediatr* 1995;**126**(4):605-10.
8. Coalson JJ. Pathology of new bronchopulmonary dysplasia. *Semin Neonatol* 2003;**8**(1):73-81.
9. Thebaud B, Goss KN, Laughon M, et al. Bronchopulmonary dysplasia. *Nat Rev Dis Primers* 2019;**5**(1):78.
10. Norman M, Hallberg B, Abrahamsson T, et al. Association Between Year of Birth and 1-Year Survival Among Extremely Preterm Infants in Sweden During 2004-2007 and 2014-2016. *JAMA* 2019;**321**(12):1188-99.
11. Qiu X, Lodha A, Shah PS, et al. Neonatal outcomes of small for gestational age preterm infants in Canada. *Am J Perinatol* 2012;**29**(2):87-94.
12. Ehrenkranz RA, Walsh MC, Vohr BR, et al. Validation of the National Institutes of Health consensus definition of bronchopulmonary dysplasia. *Pediatrics* 2005;**116**(6):1353-60.
13. Blencowe H, Cousens S, Oestergaard MZ, et al. National, regional, and worldwide estimates of preterm birth rates in the year 2010 with time trends since 1990 for selected countries: a systematic analysis and implications. *Lancet* 2012;**379**(9832):2162-72.
14. Bhandari A, McGrath-Morrow S. Long-term pulmonary outcomes of patients with bronchopulmonary dysplasia. *Semin Perinatol* 2013;**37**(2):132-7.
15. Lapcharoensap W, Lee H, Nyberg A, et al. Health Care and Societal Costs of Bronchopulmonary Dysplasia. *NeoReviews* 2018;**19**:e211-e23.
16. Evers S, Goossens M, de Vet H, et al. Criteria list for assessment of methodological quality of economic evaluations: Consensus on Health Economic Criteria. *Int J Technol Assess Health Care* 2005;**21**(2):240-5.
17. von der Warth R, Dams J, Grochtdreis T, et al. Economic evaluations and cost analyses in posttraumatic stress disorder: a systematic review. *Eur J Psychotraumatol* 2020;**11**(1):1753940.
18. Dangouloff T, Botty C, Beaudart C, et al. Systematic literature review of the economic burden of spinal muscular atrophy and economic evaluations of treatments. *Orphanet J Rare Dis* 2021;**16**(1):47.
19. OECD. Purchasing power parities (PPP) (indicator). Secondary Purchasing power parities (PPP) (indicator) 2020. https://data.oecd.org/conversion/purchasing-power-parities-ppp.htm.
20. OECD. Inflation (CPI) (Indicator). Secondary Inflation (CPI) (Indicator) 2020. https://data.oecd.org/price/inflation-cpi.htm.
21. Monset-Couchard M, Jaspar ML, de Bethmann O, et al. [Costs of the initial care of children with birth weight less than or equal to 1500 g in 1981]. *Arch Fr Pediatr* 1984;**41**(8):579-85.
22. McAleese KA, Knapp MA, Rhodes TT. Financial and emotional cost of bronchopulmonary dysplasia. *Clin Pediatr (Phila)* 1993;**32**(7):393-400.
23. Miller VL, Rice JC, DeVoe M, et al. An analysis of program and family costs of case managed care for technology-dependent infants with bronchopulmonary dysplasia. *J Pediatr Nurs* 1998;**13**(4):244-51.
24. Akman Y, Cebecý D, Özek E, et al. Effects of perinatal factors on the duration and cost of hospitalisation for preterm infants in a neonatal intensive care unit in Istanbul. *Turkish Journal of Medical Sciences* 2002;**32**:159-63.
25. Greenough A, Alexander J, Burgess S, et al. Preschool healthcare utilisation related to home oxygen status. *Arch Dis Child Fetal Neonatal Ed* 2006;**91**(5):F337-41.
26. Greenough A, Alexander J, Boorman J, et al. Respiratory morbidity, healthcare utilisation and cost of care at school age related to home oxygen status. *Eur J Pediatr* 2011;**170**(8):969-75.

For peer review only - http://bmjopen.bmj.com/site/about/guidelines.xhtml

27. Landry JS, Croitoru D, Jin Y, et al. Health care utilization by preterm infants with respiratory complications in Quebec. *Can Respir J* 2012;**19**(4):255-60.
28. Johnson TJ, Patel AL, Jegier BJ, et al. Cost of morbidities in very low birth weight infants. *J Pediatr* 2013;**162**(2):243-49 e1.
29. Alvarez-Fuente M, Arruza L, Muro M, et al. The economic impact of prematurity and bronchopulmonary dysplasia. *Eur J Pediatr* 2017;**176**(12):1587-93.
30. Patel AL, Johnson TJ, Robin B, et al. Influence of own mother's milk on bronchopulmonary dysplasia and costs. *Arch Dis Child Fetal Neonatal Ed* 2017;**102**(3):F256-F61.
31. Stevens W, Shih T, Incerti D, et al. Short-term costs of preeclampsia to the United States health care system. *Am J Obstet Gynecol* 2017;**217**(3):237-48 e16.
32. Mowitz ME, Ayyagari R, Gao W, et al. Health Care Burden of Bronchopulmonary Dysplasia Among Extremely Preterm Infants. *Front Pediatr* 2019;**7**:510.
33. Lapcharoensap W, Bennett MV, Xu X, et al. Hospitalization costs associated with bronchopulmonary dysplasia in the first year of life. *J Perinatol* 2020;**40**(1):130-37.
34. Jobe AH, Bancalari E. Bronchopulmonary dysplasia. *Am J Respir Crit Care Med* 2001;**163**(7):1723-9.
35. Greenough A, Cox S, Alexander J, et al. Health care utilisation of infants with chronic lung disease, related to hospitalisation for RSV infection. *Arch Dis Child* 2001;**85**(6):463-8.
36. van Katwyk S, Augustine S, Thebaud B, et al. Lifetime patient outcomes and healthcare utilization for Bronchopulmonary dysplasia (BPD) and extreme preterm infants: a microsimulation study. *BMC Pediatr* 2020;**20**(1):136.
37. Campbell M, McKenzie JE, Sowden A, et al. Synthesis without meta-analysis (SWiM) in systematic reviews: reporting guideline. *BMJ* 2020;**368**:l6890.
38. Landry JS, Chan T, Lands L, et al. Long-term impact of bronchopulmonary dysplasia on pulmonary function. *Can Respir J* 2011;**18**(5):265-70.
39. Williams I, Brown H, Healy P. Contextual Factors Influencing Cost and Quality Decisions in Health and Care: A Structured Evidence Review and Narrative Synthesis. *Int J Health Policy Manag* 2018;**7**(8):683-95.
40. Ridic G, Gleason S, Ridic O. Comparisons of health care systems in the United States, Germany and Canada. *Mater Sociomed* 2012;**24**(2):112-20.
41. Schmidt B, Roberts RS, Davis PG, et al. Prediction of Late Death or Disability at Age 5 Years Using a Count of 3 Neonatal Morbidities in Very Low Birth Weight Infants. *J Pediatr* 2015;**167**(5):982-6 e2.
42. Stuart BD, Sekar P, Coulson JD, et al. Health-care utilization and respiratory morbidities in preterm infants with pulmonary hypertension. *J Perinatol* 2013;**33**(7):543-7.
43. Riley GF. Administrative and claims records as sources of health care cost data. *Med Care* 2009;**47**(7 Suppl 1):S51-5.
44. Alriksson-Schmidt AI, Jeglinsky-Kankainen IFD, Jahnsen RB, et al. Flaunting our assets. Making the most of the Nordic registry goldmine: Cerebral palsy as an example. *Scand J Public Health* 2020;**48**(1):113-18.
45. Johannesson M, Jonsson, B., Jonsson, L., Kobelt, G., & Zethraeus, N. Why Should Economic Evaluations of Medical Innovations Have a Societal Perspective? *Office of health economics (OHE)* 2009(51, October 2009).
46. Curstedt T, Halliday HL, Speer CP. A unique story in neonatal research: the development of a porcine surfactant. *Neonatology* 2015;**107**(4):321-9.
47. Principi N, Di Pietro GM, Esposito S. Bronchopulmonary dysplasia: clinical aspects and preventive and therapeutic strategies. *J Transl Med* 2018;**16**(1):36.
48. Zupancic JAF. A Systematic Review of Costs Associated with Preterm Birth. In: and CoUPB, Outcomes AH, eds. Preterm Birth: Causes, Consequences, and

Prevention National Academies Press (US), 2007:688 - 724.
49. Davidson LM, Berkelhamer SK. Bronchopulmonary Dysplasia: Chronic Lung Disease of Infancy and Long-Term Pulmonary Outcomes. *J Clin Med* 2017;**6**(1).

For peer review only - http://bmjopen.bmj.com/site/about/guidelines.xhtml

Figure legends

Figure 1: Flow diagram shows the study selection process, following the PRISMA guidelines. Abbreviations: BPD = Bronchopulmonary Dysplasia

Figure 2: Overview of the costs of BPD and the study period included in each article. The numbers on the Y-axis refer to the numbers assigned to each study (Table 1).

Figure 3: Costs of the index hospitalizations for preterm infants with BPD in studies conducted between 1981 and 2015. All costs were converted to Int$, based on purchasing power parities, then the values were inflated to 2019 values. Abbreviations: BPD = Bronchopulmonary Dysplasia; control infants = preterm infants without BPD; mInt$ = millions of Int$.

Figure 4: Mean cost increase for infants with BPD, stratified by gestational age (GA) at birth. The numbers on the x-axis refer to the numbers assigned to studies in Table 1. Abbreviations: BPD = Bronchopulmonary dysplasia; Int$ = The cost after converting to purchasing power parities, then inflating to the 2019 value; mInt$ = millions of Int$, w = weeks

For peer review only

For peer review only - http://bmjopen.bmj.com/site/about/guidelines.xhtml

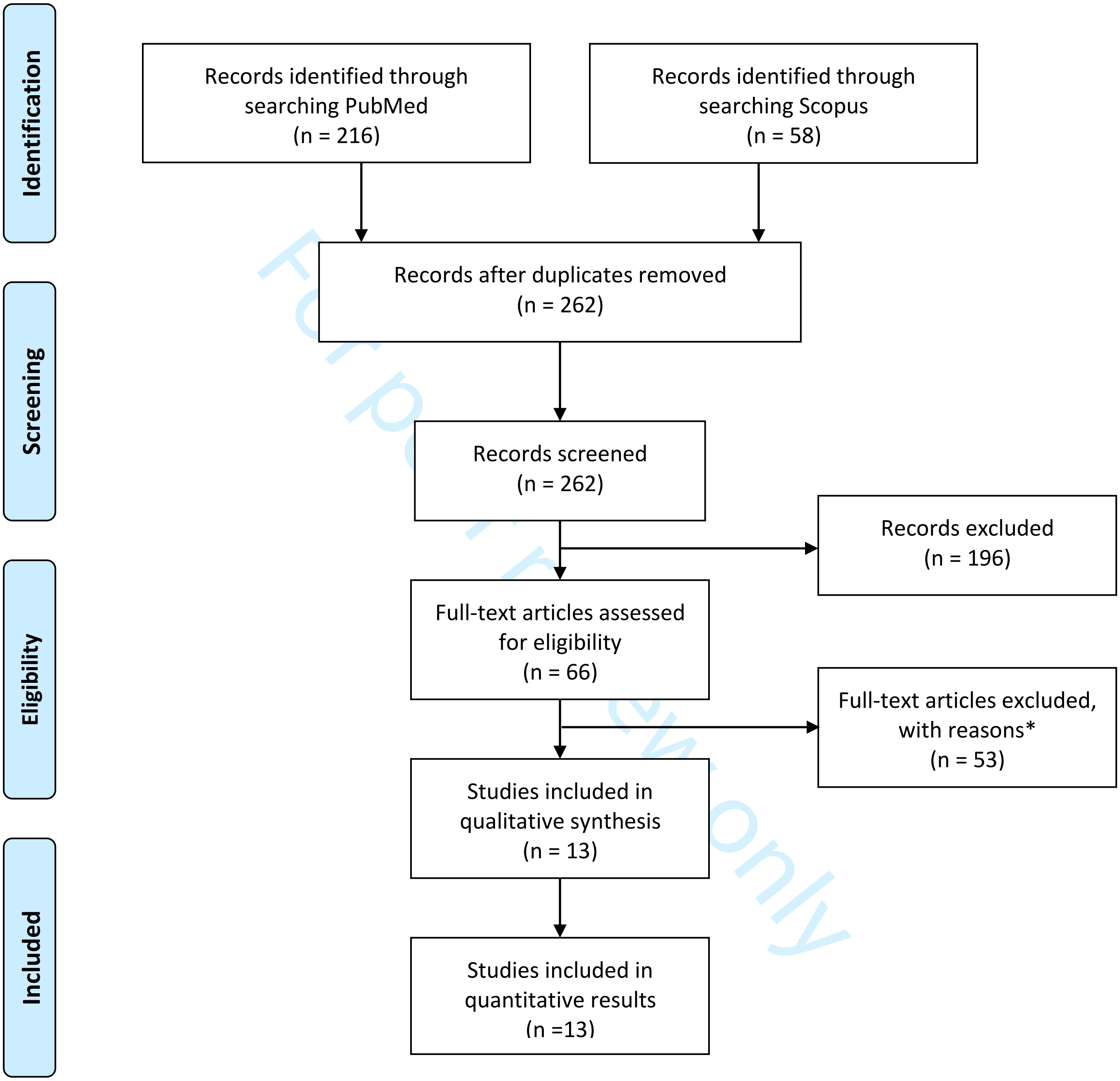

* Reasons for exclusion after full text assessment:
- Cost data not specific to BPD (n = 26)
- Only billed charges, no detailed cost estimates (n = 2)
- No cost analysis with original data (n = 25)

For peer review only

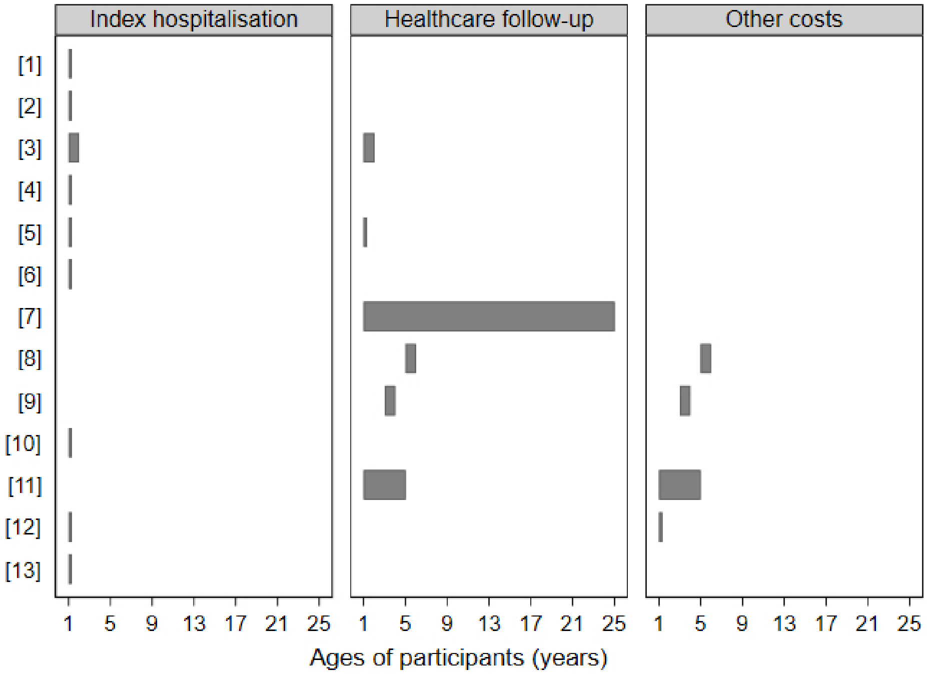

Figure 2Overview of the costs of BPD and the study period included in each article. The numbers on the Y-axis refer to the numbers assigned to each study (Table 1).

244x177mm (72 x 72 DPI)

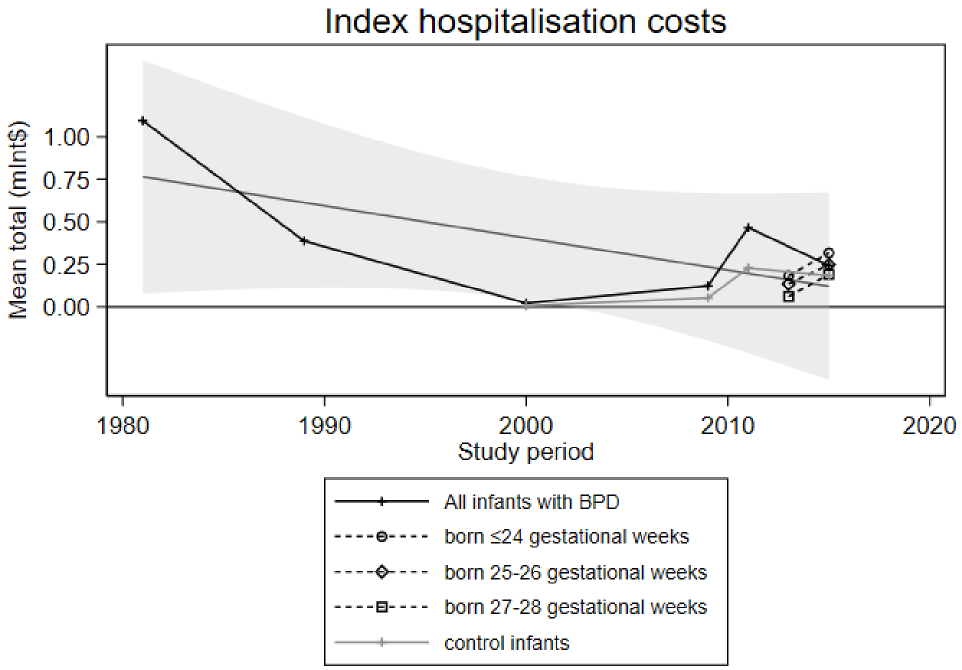

Figure 3Costs of the index hospitalizations for preterm infants with BPD in studies conducted between 1981 and 2015. All costs were converted to Int$, based on purchasing power parities, then the values were inflated to 2019 values. Abbreviations: BPD = Bronchopulmonary Dysplasia; control infants = preterm infants without BPD; mInt$ = millions of Int$.

244x177mm (72 x 72 DPI)

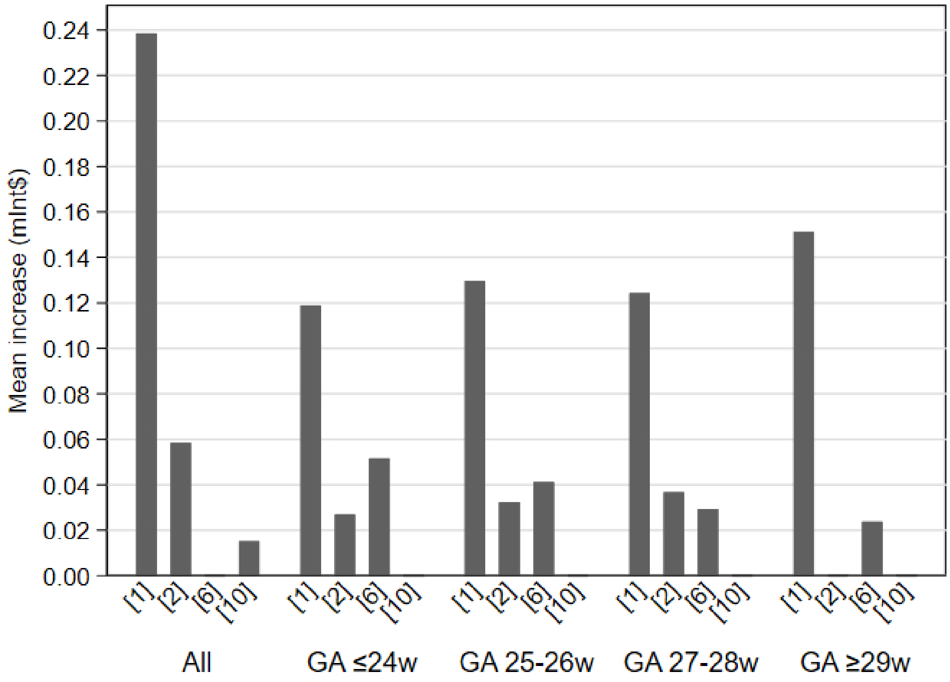

Figure 4
Mean cost increase for infants with BPD, stratified by gestational age (GA) at birth. The numbers on the x-axis refer to the numbers assigned to studies in Table 1. Abbreviations: BPD = Bronchopulmonary dysplasia; Int$ = The cost after converting to purchasing power parities, then inflating to the 2019 value; mInt$ = millions of Int$, w = weeks

244x177mm (72 x 72 DPI)

SUPPLEMENTAL MATERIAL

SYSTEMATIC REVIEW OF THE HEALTHCARE COST OF BRONCHOPULMONARY DYSPLASIA

AUTHORS

Jhangir Humayun[1,2], gushumjh@student.gu.se

Chatarina Löfqvist[1,2,3] chatarina.lofqvist@gu.se

David Ley[4], david.ley@med.lu.se

Ann Hellström[3], ann.hellstrom@medfak.gu.se

Hanna Gyllensten[1,2] hanna.gyllensten@gu.se

Author affiliations

[1] Institute of Health and Care Sciences, Sahlgrenska Academy, University of Gothenburg, Box 457, SE-405 30 Gothenburg, Sweden.

[2] Centre for Person-Centred Care (GPCC), University of Gothenburg, Box 457, SE-405 30 Gothenburg, Sweden.

[3] Department of Clinical Neuroscience, Institute of Neuroscience and Physiology, Sahlgrenska Academy, University of Gothenburg, Gothenburg, Sweden;

[4] Department of Pediatrics, Institute of Clinical Sciences Lund, Lund University and Skåne University Hospital, Lund, Sweden

Table S1: Search terms

Database	Search string
PubMed	((("Bronchopulmonary Dysplasia"[Title/Abstract]) OR "Bronchopulmonary Dysplasia"[Mesh])) AND (("Economics"[Mesh]) OR ((economic*[Title/Abstract] OR cost[Title/Abstract] OR costs[Title/Abstract] OR costly[Title/Abstract] OR costing[Title/Abstract] OR price[Title/Abstract] OR prices[Title/Abstract] OR pricing[Title/Abstract] OR pharmacoeconomic*[Title/Abstract])))
Scopus	(TITLE-ABS-KEY ("Bronchopulmonary Dysplasia")) AND (TITLE-ABS-KEY (economic* OR cost OR costs OR costly OR costing OR price OR prices OR pricing OR pharmacoeconomic*))

For peer review only

Table S2: Checklist for the quality appraisal of included papers (from [15])

Checklist items	24	33	32	29	31	30	28	27	26	25	23	22	21	Total*
Is the study population clearly described?	-	+	+	-	+	+	+	+	+	+	-	+	-	9
Are competing alternatives clearly described?	NA	NA	NA	NA	NA	+	NA	NA	+	+	NA	NA	NA	3
Is a well-defined research question posed in answerable form?	-	-	-	-	-	-	-	-	-	-	-	-	-	0
Is the economic study design appropriate to the stated objective?	+	+	+	+	+	+	+	+	+	+	+	+	+	13
Is the chosen time horizon appropriate in order to include relevant costs and consequences?	NA	NA	NA	NA	NA	NA	NA	NA	NA	NA	NA	NA	NA	0
Is the actual perspective chosen appropriate?	+	+	+	+	+	+	+	+	+	+	+	+	+	13
Are all important and relevant costs for each alternative identified?	+	+	+	+	+	+	-	+	+	+	+	+	+	12
Are all costs measured appropriately in physical units?	+	+	+	+	+	+	+	-	+	+	+	+	+	12
Are costs valued appropriately?	+	+	+	+	+	+	+	+	+	+	+	+	+	13
Are all important and relevant outcomes for each alternative identified?	NA	NA	NA	NA	NA	NA	NA	NA	NA	NA	NA	NA	NA	0
Are all outcomes measured appropriately?	NA	NA	NA	A	NA	NA	NA	NA	NA	NA	NA	NA	NA	0
Are outcomes valued appropriately?	NA	NA	NA	NA	NA	NA	NA	NA	NA	NA	NA	NA	NA	0
Is an incremental analysis of costs and outcomes of alternatives performed?	NA	NA	NA	NA	NA	NA	NA	NA	NA	NA	NA	NA	NA	0
Are all future costs and outcomes discounted appropriately?	NA	NA	NA	NA	+	NA	+	+	+	+	-	+	+	7
Are all important variables, whose values are uncertain, appropriately subjected to sensitivity analysis?	NA	-	-	-	+	-	-	-	-	-	-	-	-	1
Do the conclusions follow from the data reported?	-	+	+	+	+	+	+	+	+	+	+	+	+	12
Does the study discuss the generalizability of the results to other settings and patient/client groups?	-	+	+	+	+	+	+	+	+	+	+	+	+	12
Does the article indicate that there is no potential conflict of interest of study researcher(s) and funder(s)?	-	+	+	+	+	+	+	+	+	+	-	-	-	9
Are ethical and distributional issues discussed appropriately?	+	+	+	+	+	+	+	+	+	+	+	+	+	13
Number of checklist items fulfilled by each individual study	6	10	10	9	12	11	10	10	12	12	8	10	9	

+ indicates a fulfilled checklist item, - indicates that the item was either not stated or not fulfilled, NA indicates that the checklist item was not relevant for that study

* Number of studies that fulfilled each checklist item (not considering the applicability of the checklist item to the individual studies

For peer review only

Section/topic	#	PRISMA checklist item	Reported on page #
**TITLE**			
Title	1	Identify the report as a systematic review, meta-analysis, or both.	1
**ABSTRACT**			
Structured summary	2	Provide a structured summary including, as applicable: background; objectives; data sources; study eligibility criteria, participants, and interventions; study appraisal and synthesis methods; results; limitations; conclusions and implications of key findings; systematic review registration number.	2
**INTRODUCTION**			
Rationale	3	Describe the rationale for the review in the context of what is already known.	4
Objectives	4	Provide an explicit statement of questions being addressed with reference to participants, interventions, comparisons, outcomes, and study design (PICOS).	5
**METHODS**			
Protocol and registration	5	Indicate if a review protocol exists, if and where it can be accessed (e.g., Web address), and, if available, provide registration information including registration number.	6
Eligibility criteria	6	Specify study characteristics (e.g., PICOS, length of follow-up) and report characteristics (e.g., years considered, language, publication status) used as criteria for eligibility, giving rationale.	6
Information sources	7	Describe all information sources (e.g., databases with dates of coverage, contact with study authors to identify additional studies) in the search and date last searched.	6
Search	8	Present full electronic search strategy for at least one database, including any limits used, such that it could be repeated.	Table S1
Study selection	9	State the process for selecting studies (i.e., screening, eligibility, included in systematic review, and, if applicable, included in the meta-analysis).	6
Data collection process	10	Describe method of data extraction from reports (e.g., piloted forms, independently, in duplicate) and any processes for obtaining and confirming data from investigators.	6-7
Data items	11	List and define all variables for which data were sought (e.g., PICOS, funding sources) and any assumptions and simplifications made.	7
Risk of bias in individual studies	12	Describe methods used for assessing risk of bias of individual studies (including specification of whether this was done at the study or outcome level), and how this information is to be used in any data synthesis.	7
Summary measures	13	State the principal summary measures (e.g., risk ratio, difference in means).	7
Synthesis of results	14	Describe the methods of handling data and combining results of studies, if done, including measures of consistency (e.g., I^2) for each meta-analysis.	7

For peer review only

Section/topic	#	PRISMA checklist item	Reported on page #
Risk of bias across studies	15	Specify any assessment of risk of bias that may affect the cumulative evidence (e.g., publication bias, selective reporting within studies).	NA
Additional analyses	16	Describe methods of additional analyses (e.g., sensitivity or subgroup analyses, meta-regression), if done, indicating which were pre-specified.	7
**RESULTS**			
Study selection	17	Give numbers of studies screened, assessed for eligibility, and included in the review, with reasons for exclusions at each stage, ideally with a flow diagram.	7<br>Figure 1
Study characteristics	18	For each study, present characteristics for which data were extracted (e.g., study size, PICOS, follow-up period) and provide the citations.	Table 1
Risk of bias within studies	19	Present data on risk of bias of each study and, if available, any outcome level assessment (see item 12).	10<br>Table S2
Results of individual studies	20	For all outcomes considered (benefits or harms), present, for each study: (a) simple summary data for each intervention group (b) effect estimates and confidence intervals, ideally with a forest plot.	Table 2
Synthesis of results	21	Present results of each meta-analysis done, including confidence intervals and measures of consistency.	NA
Risk of bias across studies	22	Present results of any assessment of risk of bias across studies (see Item 15).	10
Additional analysis	23	Give results of additional analyses, if done (e.g., sensitivity or subgroup analyses, meta-regression [see Item 16]).	12
**DISCUSSION**			
Summary of evidence	24	Summarize the main findings including the strength of evidence for each main outcome; consider their relevance to key groups (e.g., healthcare providers, users, and policy makers).	12
Limitations	25	Discuss limitations at study and outcome level (e.g., risk of bias), and at review-level (e.g., incomplete retrieval of identified research, reporting bias).	13
Conclusions	26	Provide a general interpretation of the results in the context of other evidence, and implications for future research.	13
**FUNDING**			
Funding	27	Describe sources of funding for the systematic review and other support (e.g., supply of data); role of funders for the systematic review.	14

Abbreviations: NA = Not applicable.

For peer review only
